# Recurrence rate and risk factors of recurrence after antiseizure medications withdrawal in children with epilepsy: a meta-analysis

**DOI:** 10.3389/fped.2026.1827056

**Published:** 2026-07-27

**Authors:** Hechengyue Lv, Rui Wang, Yueyue He, Nan Wen, Weixiu Ouyang, Jianyu Peng, Qian Jiang, Junli Zhang, Ling Feng

**Affiliations:** 1 Department of Neurology, West China Hospital, Sichuan University/West China School of Nursing, Sichuan University, Chengdu, Sichuan, China; 2 Department of Neurology, West China Tianfu Hospital, Sichuan University, Chengdu, Sichuan, China; 3 Department of Emergency, West China Tianfu Hospital, Sichuan University, Chengdu, Sichuan, China; 4 Department of Gastroenterology, West China Hospital, Sichuan University/West China School of Nursing, Sichuan University, Chengdu, Sichuan, China

**Keywords:** children, epilepsy, meta-analysis, recurrence, risk factors

## Abstract

**Objective:**

This study aims to explore the recurrence rate and risk factors for recurrence after antiseizure medications withdrawal in children with epilepsy through a meta-analysis.

**Methods:**

Databases were searched up to May 8, 2025. Two reviewers independently screened the literature, extracted data, and assessed study quality. A meta-analysis was performed using Stata 15.0.

**Results:**

Nineteen studies involving 3,226 children were included. The pooled recurrence rate was 28% (95% CI: 23%–33%). Risk factors included female gender, focal seizure, history of febrile seizures, delayed development/intellectual disability, symptomatic etiology, abnormal electroencephalogram (EEG) after withdrawal.

**Conclusion:**

Multiple factors increase recurrence risk after antiseizure medications withdrawal in children, supporting individualized clinical decision-making.

## 1 Introduction

Epilepsy is a brain disorder characterized by a persistent predisposition to generate epileptic seizures (1). As one of the most prevalent severe chronic neurological disorders worldwide, epilepsy affects individuals across all age groups, from infancy to old age. The condition may result in motor impairments and intellectual disabilities of varying severity. In pediatric patients, epilepsy can additionally exert adverse effects on physical growth and neurodevelopment (2–4). Moreover, the health-related and social burdens associated with epilepsy are substantial. According to global estimates, the health burden attributable to epilepsy ranked seventh among neurological diseases worldwide in 2021 (5). Between 1990 and 2021, the global prevalence of epilepsy increased by 57.4%, with the number of affected individuals reaching 24.4 million and epilepsy-related deaths rising to 0.14 million (5). Additionally, among the 5–19-year-old age group, epilepsy is the third leading cause of loss of healthy life years, highlighting its ongoing impact on population health (5). This burden is disproportionately higher in low- and middle-income countries, where epilepsy-related premature mortality remains a major public health concern (6). Therefore, in-depth research on epilepsy is essential for reducing its global disease burden and improving patients' quality of life.

Antiseizure medications (ASMs) are the major treatment intervention used to control seizures in epileptic patients. Nevertheless, long-term ASMs use can be linked to unfavorable effects across several systems, most notably the nervous system (7). Gradual reduction of doses and eventual withdrawal of ASMs is usually considered in patients who are seizure-free for a long period. However, the most appropriate timing or methods of ASMs withdrawal, especially in children, have not yet been agreed upon. Recent guidelines developed by the International League Against Epilepsy (ILAE) have tried to summarize existing evidence and offer systematic advice about withdrawal decisions, although individual risk assessment (particularly in children) is necessary (8). Additionally, the available literature suggests that the recurrence of seizures can be substantial among patients even following ASMs withdrawal. Recurrence rates reported after ASMs withdrawal in children across studies differ widely, ranging between 10% and 52% (9–26). This variability can be caused not just by differences in sample characteristics and follow-up periods but also by different definitions of remission and withdrawal eligibility across studies and geographical regions (27). These inconsistencies are probably due to synergistic effects of clinical heterogeneity, methodological variability, and disease severity across studies. The recurrence of epilepsy has a negative impact on the physical and psychological health of children and gives rise to enormous expenses for families and society. Thus, effectively evaluating recurrence risk in children without seizures are important to clinicians, families, and healthcare systems.

Many studies have been conducted to examine the recurrence of seizures after ASMs withdrawal in children with epilepsy. Risk factors proposed include clinical factors, etiological factors, electrophysiological findings, and variables associated with treatment. Nonetheless, there is considerable heterogeneity in the literature in terms of sample size, research design, and variables measured, leading to mixed conclusions and constraining the precise estimation of the risk of recurrence and its predictors. The current research integrated the existing information with the help of meta-analysis, involving quantitative pooling and analysis of the available data. The method allows combining the results of various studies, minimizing the effects of bias from individual studies, and increasing the credibility and applicability of the conclusions. Thus, the proposed study will help to better understand the frequency and risk factors of seizure recurrence following ASMs withdrawal in children with epilepsy, thereby providing a more solid evidence base upon which individual clinical decisions and withdrawal plans may be tailored to decrease the risk of seizure recurrence and enhance prognosis.

## 2 Materials and methods

This study was conducted following the guidelines outlined in the Preferred Reporting Items for Systematic Review and Meta-Analysis Protocols (PRISMA-P). The review was performed in line with the PRISMA criteria (28). The registration number is CRD420251080587.

### 2.1 Literature search

PubMed, Embase, Web of Science, and the Cochrane Library were systematically searched from database inception to May 8, 2025. The subject terms used were: Epilepsy; Child; Recurrence; Risk Factors. Relevant free terms were also added for the search. Details of the search approach can be found in Supplementary Table S1.

### 2.2 Inclusion and exclusion criteria

#### 2.2.1 Inclusion criteria

1. The study type was a case-control study, cohort study, or cross-sectional study;
2. The study population consisted of children (0–18 years old) who, after being treated with ASMs, were assessed by physicians as eligible for ASMs withdrawal;
3. The outcome indicator was the recurrence of epilepsy after ASMs withdrawal;
4. Complete data could be extracted from the articles;
5. The literature was published in English.

#### 2.2.2 Exclusion criteria

1. Systematic reviews, case reports, conference abstracts, and articles on animal experiments;
2. Studies including surgical treatment or non-ASM therapy administered prior to ASMs withdrawal;
3. Duplicate publications;
4. Studies from which the complete text could not be accessed, or for which statistical indicators could not be extracted or calculated;
5. Non-English literature.

### 2.3 Data extraction

Two reviewers independently screened titles and abstracts for eligibility, followed by full-text assessment for inclusion. Any disagreements were resolved through discussion or consultation with a third reviewer. Extracted data included the first author, publication year, country, study design, sample size, recurrence outcomes, patient age and gender, and the multivariate analysis regression model used in the analysis.

### 2.4 Quality assessment

All studies included in this paper were either case-control studies or cohort studies. The quality of the included studies was jointly assessed by two researchers using the Newcastle-Ottawa Scale (NOS) (29), which encompasses three components: the study population (4 points), group comparability (2 points), and the evaluation of exposure variables or outcomes (3 points). The scale has an overall score of 9 points, where scores of ≤4, 5–6, and ≥7 signify low, moderate, and high quality, respectively. If the two researchers held differing views while conducting the assessment, they engaged in discussion to reach a decision or consulted a third party for advice.

### 2.5 Statistical analysis

The data underwent statistical analysis with the use of Stata 15.0. The risk indicators of each study are presented in the form of their respective hazard ratio (HR) and odds ratio (OR) values that can be extracted, accompanied by the computation of the corresponding 95% confidence intervals (CIs). For the meta-analysis of recurrence rate, we extracted the total sample size and number of recurrence cases from all included cohort studies to calculate the pooled recurrence rate and its standard error of proportion, and the results were visualized via forest plots. Meta-regression analysis was also performed to explore the influence of common covariates. Heterogeneity was assessed using the *Q* test and *I*^2^ statistic to select the suitable model for pooling effect sizes. In the case of *I*^2^ > 50%, indicating significant heterogeneity, a random-effects model was employed; a fixed-effects model was used otherwise. In cases of significant heterogeneity (*I*^2^ > 50%), sensitivity analysis was conducted to investigate the causes of heterogeneity and ensure the robustness of the results. Moreover, funnel plots were used to visually screen for publication bias, and the Egger test was used as a statistical test to evaluate publication bias. In case of evidence of publication bias, the trim-and-fill technique would be used to determine the stability of the results, and a significance level of 0.05 would be used.

## 3 Results

### 3.1 Literature search results

**Figure 1:**
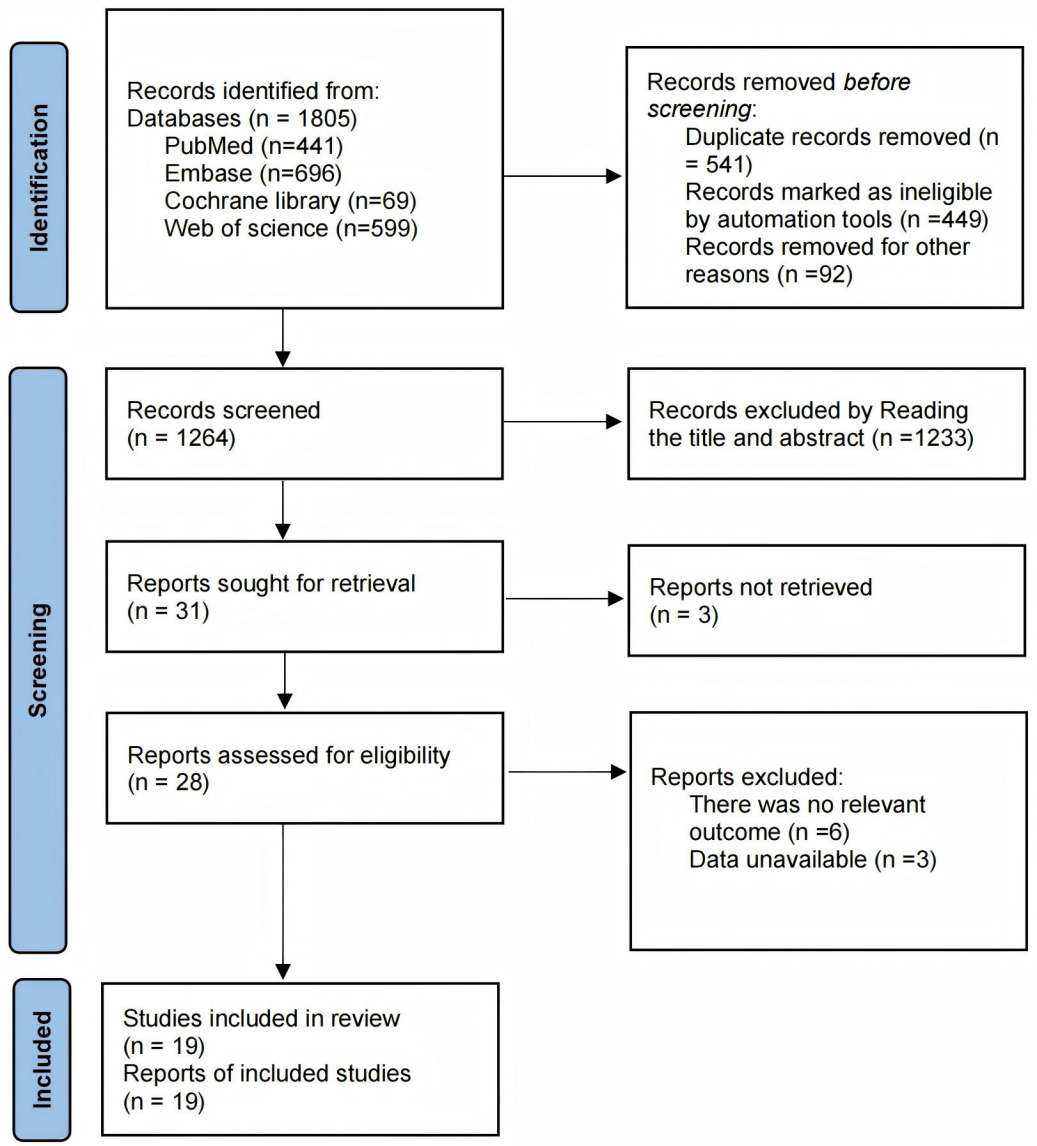
Flow chart of the literature search.

The databases initially yielded 1,805 records. Following the elimination of the duplicate records, 1,264 articles remained. After the screening of titles and abstracts, 31 studies were found to be potentially eligible. Among them, three articles were ruled out due to the inability to access the full text. Following full-text evaluation, 19 studies were found to satisfy the inclusion criteria and were finally incorporated into the meta-analysis (See Figure 1).

### 3.2 Basic characteristics of the included studies

Among the included studies, 18 (9–26) were cohort studies and one (30) was a case–control study. In total, 3,226 children with epilepsy were included, of whom 865 experienced seizure recurrence after ASMs withdrawal and 2,361 remained seizure-free. Methodological quality assessment using the Newcastle–Ottawa Scale revealed scores ranging from 7 to 9 points, indicating that all included studies were of high methodological quality. Detailed study characteristics are presented in Table 1, and individual NOS scores are shown in Supplementary Tables S2, S3.

**Table 1:** Table of literature characteristics.

Study	Year	Country	Study design	Sample size		Age (years)		Gender (M/F)	Types of epilepsy included	Follow-up period (years)	Regression model	NOS scores
				R	NR	R	NR					
Shinnar et al. (21)	1994	USA	Cohort	95	169	<18		136/128	F&G&F + G	4.83	MC	8
Dooley et al. (11)	1996	England	Cohort	37	60	<18		50/47	F&G	2.7 ± 1.09	MC	8
Caviedes and Herranz (10)	1998	Spain	Cohort	55	171	<15		116/110	F&G	5.85 ± 3.87	MC	8
Altunbaşak et al. (9)	1999	Turkey	Cohort	20	77	12.5 ± 3.6		58/39	F&G	2–4	Mlr&MC	8
Gebremariam et al. (12)	1999	Ethiopia	Cohort	26	54	<18		55/25	F&G&F + G	2.59	MC	7
Ohta et al. (16)	2004	Japan	Cohort	8	74	<18		47/35	FEUC	4.58	MC	8
Olmez et al. (17)	2009	Turkey	Cohort	54	146	1–15		118/82	F&G&F + G	3.33 ± 3.18	MC	8
Ramos-Lizana et al. (20)	2010	Spain	Cohort	56	160	<14		120/96	F&G&F + G	4.92	MC	8
Vurucu et al. (24)	2010	Turkey	Cohort	51	215	<18		155/111	F&G	≥2	Mlr	8
Pavlović et al. (18)	2011	Serbia	Cohort	23	21	<18		15/29	GGE	2–10	MC	8
Pavlović et al. (19)	2012	Serbia	Cohort	19	33	<18		36/16	FEUC	2–13	MC	8
Verrotti et al. (23)	2012	Italy	Cohort	48	120	<18		74/94	F&G&F + G	11.54	MC	8
Srivastava et al. (22)	2017	India	Cohort	47	101	<18		96/52	F&G&F + G	2.06	MC	8
Karalok et al. (14)	2020	Turkey	Cohort	51	233	<18		147/137	F&G&F + G	8.3	MC	8
Koken et al. (15)	2021	Turkey	Cohort	115	135	12.98 ± 4.73		129/121	GGE	8.17 ± 3.08	Mlr	8
Yildirim et al. (25)	2022	Turkey	Cohort	90	179	<18		157/112	F&G&F + G	4.02 ± 1.94	Mlr	8
Kanmaz et al. (13)	2023	Turkey	Cohort	51	352	<18		222/181	F&G&F + G	3.17 ± 2.53	MC	8
Zhao et al. (26)	2023	China	Cohort	19	61	<18		45/35	F&G&F + G	2–7	Mblr	9
Triono et al. (30)	2024	Indonesia	Case-control	63	63	12.71 ± 0.67	9.83 ± 0.54	NM	F&G&F + G	≥2	Mlr	8

R, recurrence; NR, no recurrence; NM, not mentioned; F&G, focal onset seizures & generalized onset seizures; F&G&F + G, focal onset seizures & generalized onset seizures & focal and generalized onset seizures combined; FEUC, focal epilepsy of unknown cause; GGE, genetic generalized epilepsy; Mlr, multivariate logistic regression; MC, multivariate Cox proportional hazards model; Mblr, multivariate binary logistic regression.

### 3.3 Recurrence rate

**Figure 2:**
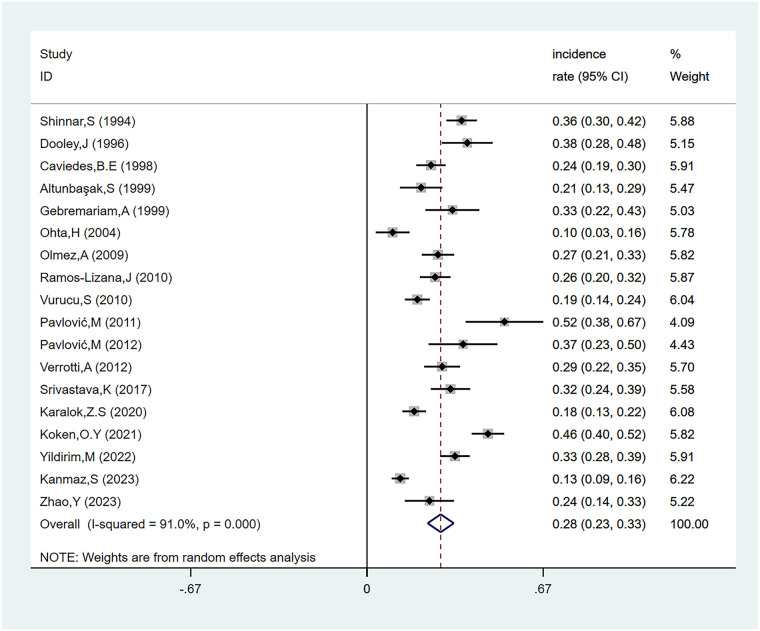
Forest plot of recurrence rate.

Eighteen studies reported recurrence rates after ASMs withdrawal in children with epilepsy. A random-effects model was applied due to substantial heterogeneity among studies (*I*^2^ = 91.0%, *p* < 0.001). The pooled recurrence rate was 28% (95% CI: 23%–33%). Sensitivity analysis demonstrated that the pooled estimate was stable. Funnel plot asymmetry and Egger's test (*p* = 0.007) suggested potential publication bias; however, the trim-and-fill analysis showed no change in the pooled estimate, indicating that the results were robust (see Figure 2, Supplementary Figures S1, S2).

#### 3.3.1 Subgroup analysis and meta-regression

**Figure 3:**
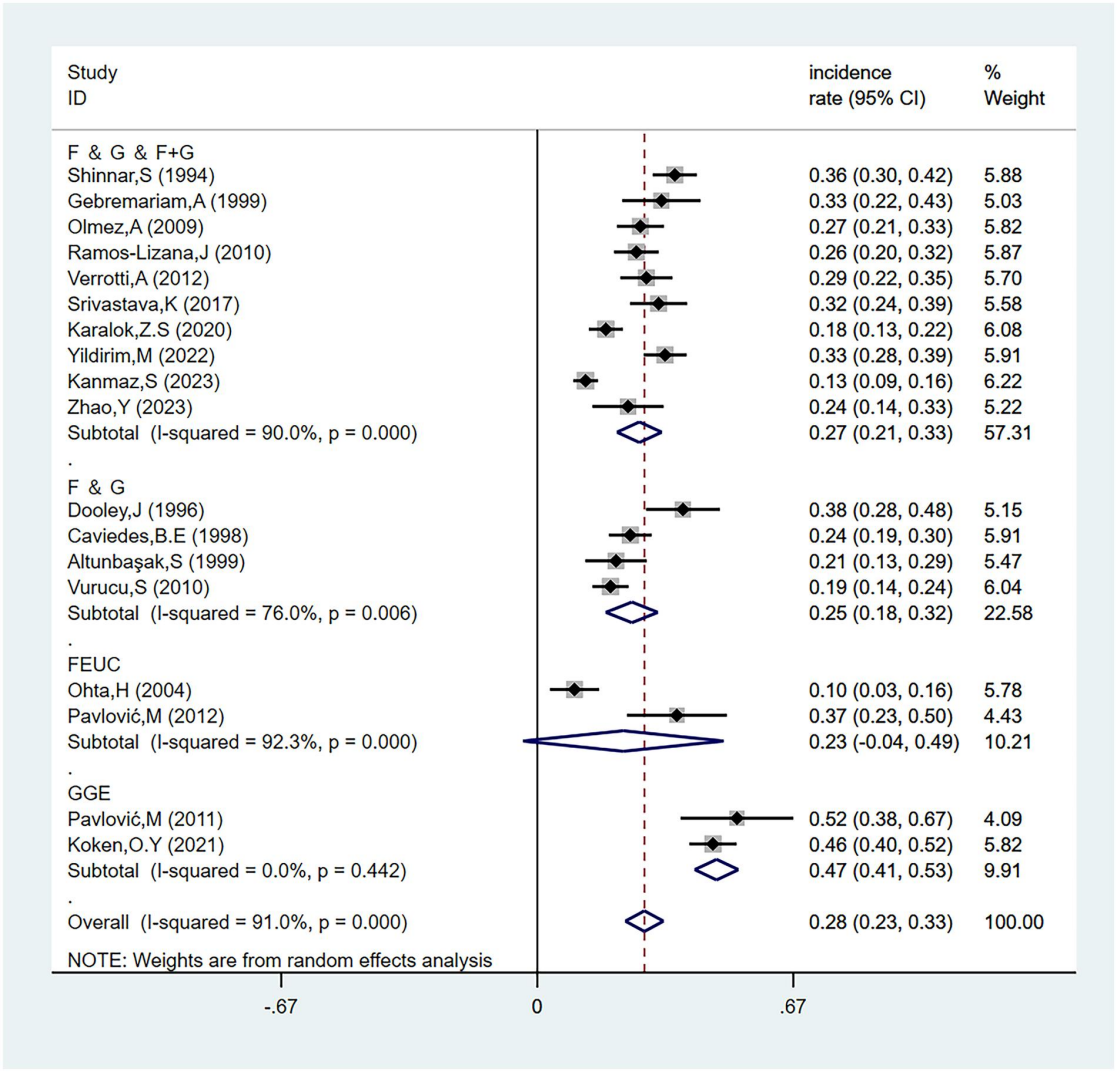
Forest plot of recurrence rates for subgroups of epilepsy types.

To explore the substantial heterogeneity observed in the overall analysis (*I*^2^ = 91.0%), subgroup analyses were conducted based on composition of seizure types included in the studies. As illustrated in Figure 3. The pooled recurrence rates varied notably across these subgroups. Studies that included a broad spectrum of seizure types—specifically focal onset, generalized onset, and combined focal and generalized seizures—reported a pooled recurrence rate of 27% (95% CI: 21%–33%; *I*^2^ = 90.0%). Similarly, studies focusing on focal and generalized onset seizures showed a comparable recurrence rate of 25% (95% CI: 18%–32%; *I*^2^ = 76.0%).

In contrast, studies with more specific population definitions yielded different estimates. The subgroup consisting exclusively of Genetic Generalized Epilepsy (GGE) demonstrated the highest pooled recurrence rate at 47% (95% CI: 41%–53%), with no significant heterogeneity detected within this group (*I*^2^ = 0.0%, *p* = 0.442). Conversely, the subgroup for Focal Epilepsy of Unknown Cause (FEUC) reported a lower pooled estimate of 23%, although the confidence interval was wide due to high heterogeneity (*I*^2^ = 92.3%).

Furthermore, univariate meta-regression analysis was performed to identify potential sources of heterogeneity (see Table 2). Among the covariates examined—including research year, follow-up duration, sample size, continent, and seizure type composition—only the inclusion of GGE patients (GGE vs. F&G&F + G) was found to be a statistically significant predictor of recurrence rates (*p* = 0.011). This variable explained approximately 34.46% of the between-study heterogeneity (Adjusted *R*^2^ = 34.46%). Other factors such as sample size, follow-up duration, and geographic location did not show significant associations with the recurrence rate (*p* > 0.05).

**Table 2:** Univariate meta-regression analysis of covariates for recurrence rate.

Covariates	Regression coefficient (Coeff.)	Standard error	95% CI	*p*	Adjusted *R*^2^ (proportion of heterogeneity explained, %)
Research year	−0.002	0.005	(−0.013, 0.009)	0.702	−6.32
Follow-up duration (years)	−0.010	0.017	(−0.046, 0.027)	0.589	−6.47
Sample size	−0.000	0.001	(−0.002, 0.000)	0.185	6.59
Continent (Europe vs. Asia)	0.141	0.102	(−0.076, 0.358)	0.188	2.21
Continent (North America vs. Asia)	0.162	0.205	(−0.273, 0.596)	0.442	−2.48
Continent (Africa vs. Asia)	0.105	0.228	(−0.379, 0.589)	0.651	−5.21
Seizure type (F&G vs. F&G&F + G)	−0.062	0.119	(−0.314, 0.189)	0.607	−5.69
Seizure type (FEUC vs. F&G&F + G)	−0.151	0.168	(−0.506, 0.203)	0.380	−0.28
Seizure type (GGE vs. F&G&F + G)	0.387	0.135	(0.100, 0.674)	0.011	34.46

F&G, focal onset seizures & generalized onset seizures; F&G&F + G, focal onset seizures & generalized onset seizures & focal and generalized onset seizures combined; FEUC, focal epilepsy of unknown cause; GGE, genetic generalized epilepsy.

### 3.4 Univariate meta-analysis

#### 3.4.1 Female gender

**Figure 4:**
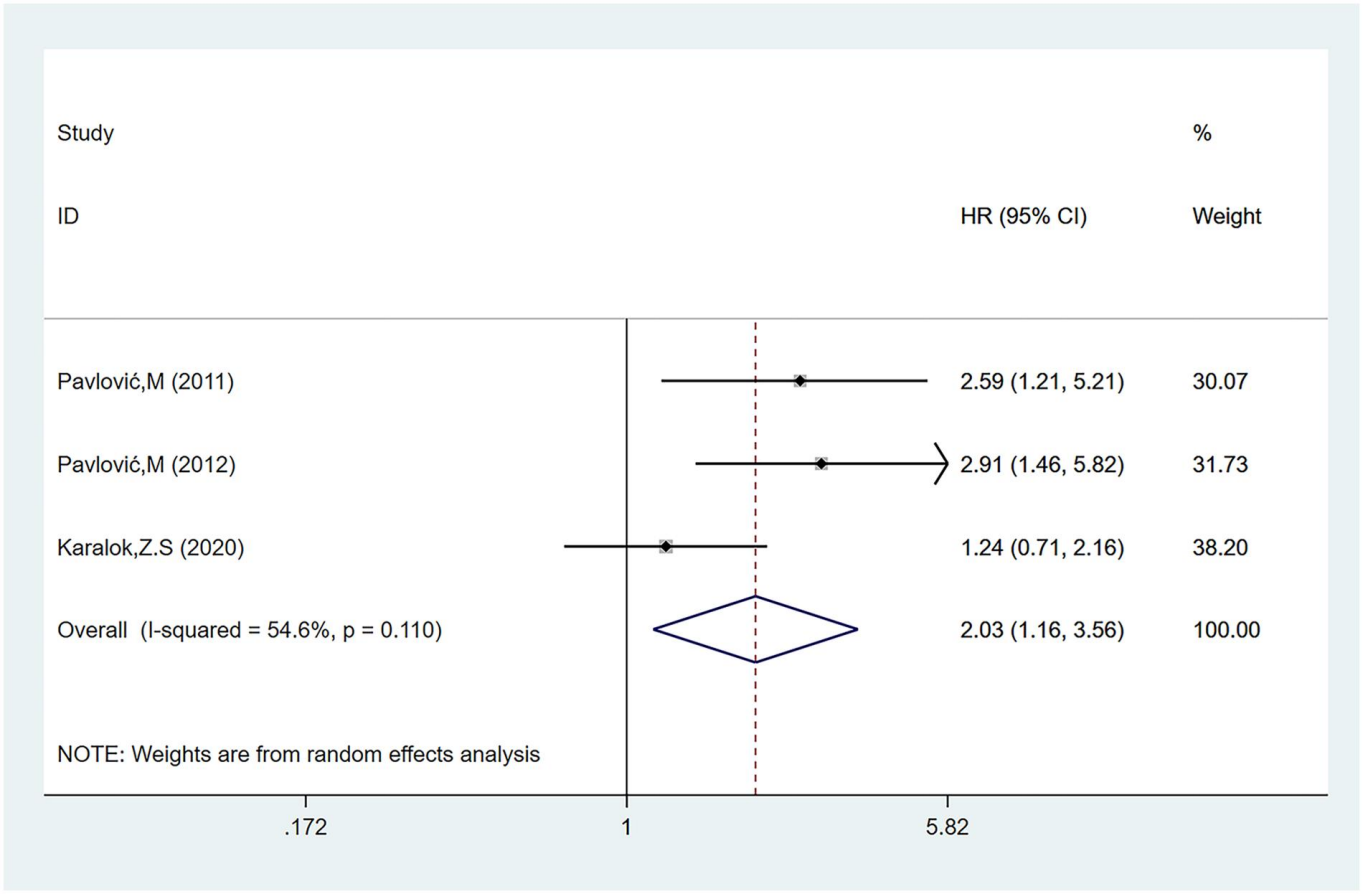
Forest plot of female gender (single factor).

Three studies were included (14, 18, 19). Heterogeneity testing yielded results of *I*^2^ = 54.6% and *p* = 0.110, prompting the use of a random-effects model in the meta-analysis. The results indicated that female sex constitutes a risk factor for recurrence following ASMs discontinuation, with the difference reaching statistical significance (HR = 2.03, 95% CI 1.16–3.56, *p* = 0.014). Additionally, a sensitivity analysis was carried out, and the results indicated low sensitivity and stable results (see Figure 4, Supplementary Figure S3, Supplementary Table S4).

#### 3.4.2 Focal seizure

**Figure 5:**
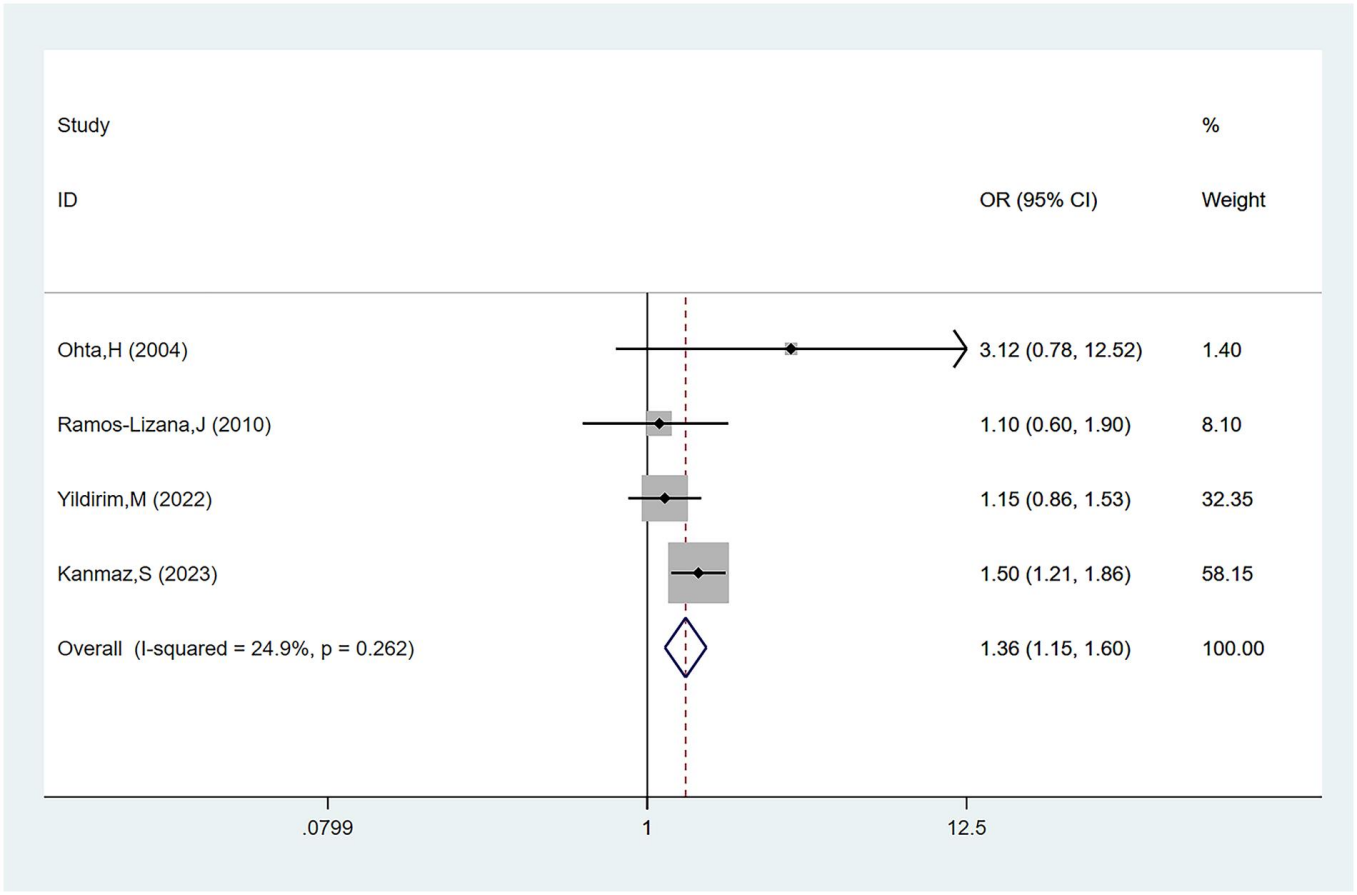
Forest plot of focal seizure (single factor).

Four studies were included (13, 16, 20, 25). Heterogeneity testing yielded results of *I*^2^ = 24.9% (*p* = 0.262), so a fixed-effects model was employed in the meta-analysis. It was found that focal seizures act as a risk factor for recurrence following ASMs discontinuation, with a statistically significant difference (OR = 1.36, 95% CI 1.15–1.60, *p* < 0.001) (see Figure 5, Supplementary Table S4).

#### 3.4.3 History of febrile seizure

**Figure 6:**
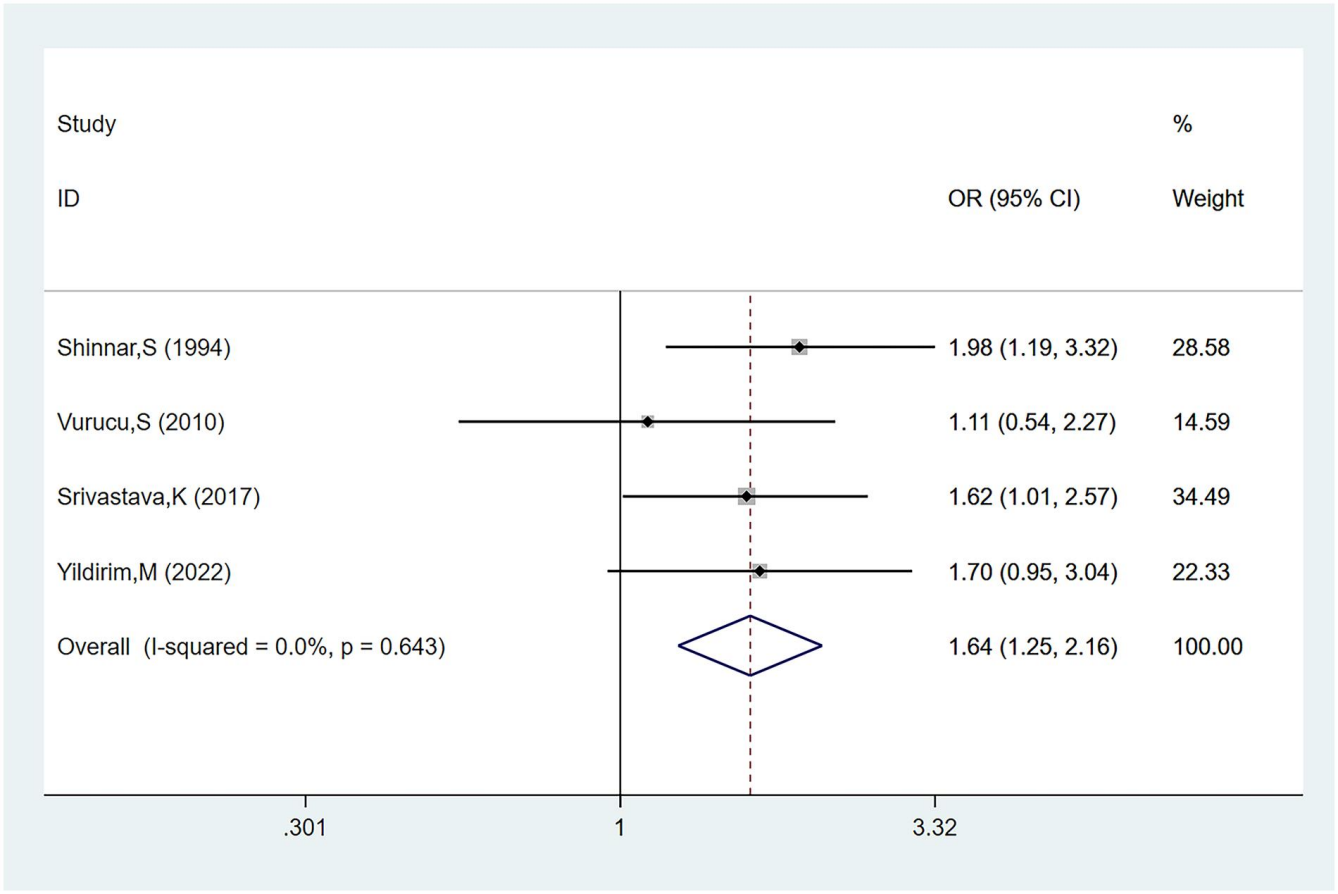
Forest plot of history of febrile seizure (single factor).

Four studies were included (21, 22, 24, 25). Heterogeneity testing returned results of *I*^2^ = 0.0% (*p* = 0.643), prompting the use of a fixed-effects model in the meta-analysis. Findings indicated that a history of febrile seizures serves as a risk factor for recurrence following ASMs discontinuation, with a statistically significant difference (OR = 1.64, 95% CI 1.25–2.16, *p* < 0.001) (see Figure 6, Supplementary Table S4).

#### 3.4.4 Delayed development/intellectual disability

**Figure 7:**
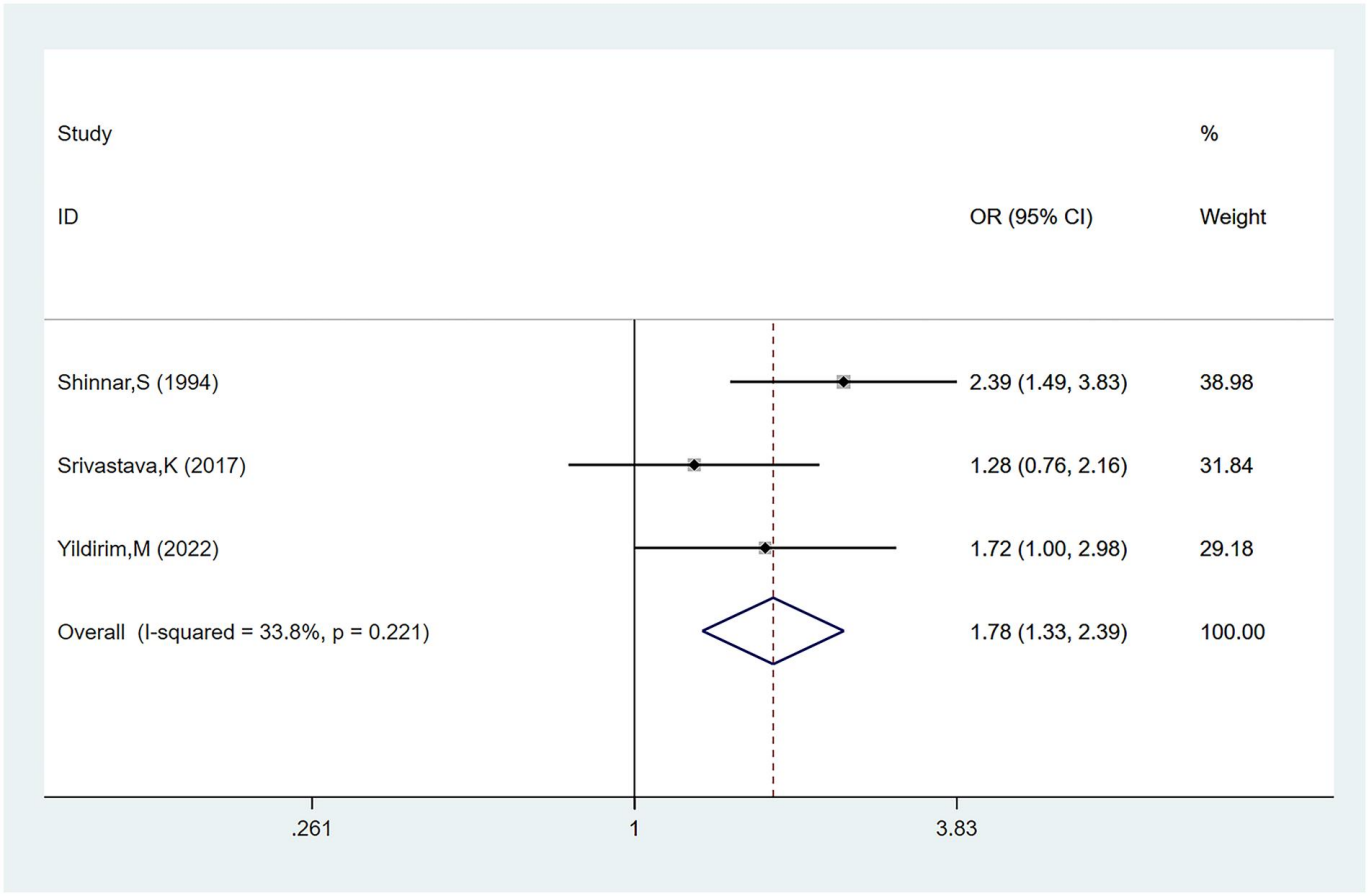
Forest plot of delayed development or intellectual disability (single factor).

Three studies were included (21, 22, 25). Heterogeneity testing yielded an *I*^2^ = 33.8% (*p* = 0.221), which resulted in the adoption of a fixed-effects model for the meta-analysis. Findings revealed that delayed development/intellectual disability constitutes a risk factor for recurrence following ASMs discontinuation, with statistical significance observed (OR = 1.78, 95% CI 1.33–2.39, *p* < 0.001) (see Figure 7, Supplementary Table S4).

#### 3.4.5 Symptomatic etiology

**Figure 8:**
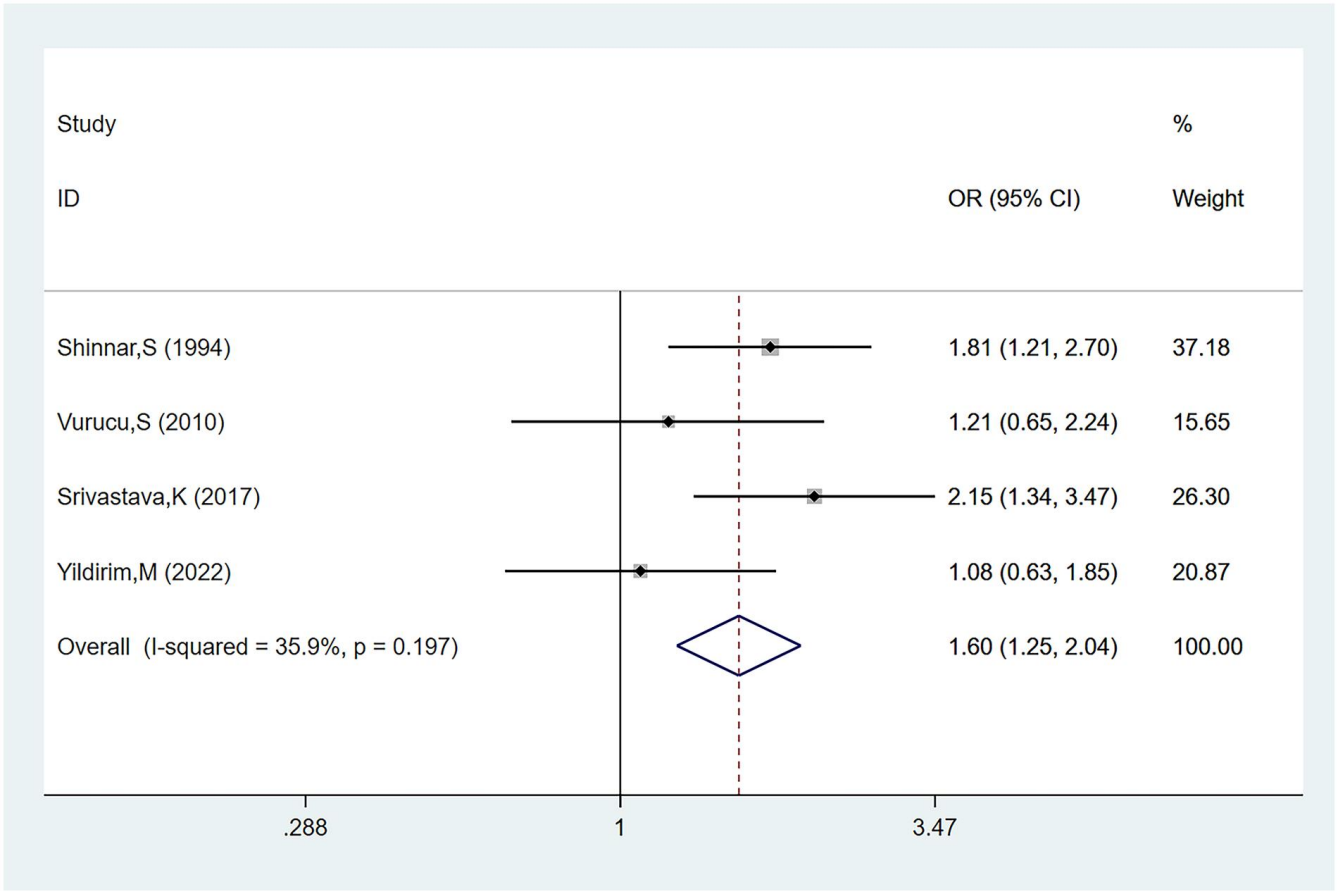
Forest plot of symptomatic etiology (single factor).

Four studies were included (21, 22, 24, 25). Heterogeneity analysis returned an *I*^2^ = 35.9% (*p* = 0.197), prompting the application of a fixed-effects model in the meta-analysis. Findings indicated that symptomatic etiology acts as a risk factor for recurrence following ASMs discontinuation, with a statistically significant difference (OR = 1.60, 95% CI 1.25–2.04, *p* < 0.001) (see Figure 8, Supplementary Table S4).

### 3.5 Results of multivariate meta-analysis

The results of multivariate analyses reported in the included studies were pooled. The findings of this research indicated that female gender (HR = 1.97, 95% CI 1.41–2.76, *p* < 0.001), abnormal EEG after withdrawal (OR = 3.11, 95% CI 1.85–5.24, *p* < 0.001), history of febrile seizure (OR = 2.21, 95% CI 1.24–3.95, *p* = 0.007), delayed development/intellectual disability (OR = 1.86, 95% CI 1.25–2.75, *p* < 0.001) can be regarded as risk factors for recurrence following ASMs discontinuation (see Supplementary Figures S4–S7, Supplementary Table S5).

### 3.6 Other meta-analysis results

No statistically significant associations were observed between seizure recurrence after ASMs withdrawal and the following factors: family history of epilepsy, history of status epilepticus, age over 10 years at withdrawal, clinical evidence of neurologic abnormalities, seizures before treatment ≥2 (see Supplementary Figures S8–S15, Supplementary Tables S4, S5).

### 3.7 Publication bias

Funnel plots and Egger's test were employed to evaluate publication bias for every risk factor. The funnel plots for all risk factors were basically symmetric, and Egger's test results indicated a *p*-value >0.05, with no publication bias detected (see Supplementary Figures S16–S30, Supplementary Tables S4, S5).

## 4 Discussion

ASMs are the mainstay treatment for childhood epilepsy. After long-term seizure freedom, the decision to discontinue drugs is a common clinical dilemma for pediatric neurologists and caregivers, as seizure relapse after drug withdrawal will not only increase the risk of repeated treatment and adverse reactions, but also bring psychological and economic burdens to children and their families. This meta-analysis systematically integrated relevant clinical studies to explore the overall relapse rate and independent prognostic factors of childhood epilepsy after ASM withdrawal.

### 4.1 Recurrence rate

This meta-analysis found that the pooled relapse rate of children with epilepsy after ASM withdrawal (28%, 95% CI: 23%–33%) was consistent with the overall level reported in meta-analyses focused on ASM discontinuation. Berg and Shinnar conducted a classic meta-analysis covering children and adult patients with epilepsy, and reported that the 1-year and 2-year relapse rates after drug withdrawal were 25% and 29% respectively (31). A prospective cohort study by Ramos-Lizana et al. targeting children under 14 years old showed that the 2- and 5-year cumulative relapse rates after drug withdrawal were 23% and 28% (20) which was similar to the pooled relapse rate in the present study. This cross-study consistency supports the reliability of our pooled estimate. In contrast to earlier meta-analyses that often included mixed pediatric and adult populations (31), our study strictly included children only. Given that adolescent- or adult-onset epilepsy may carry a higher relapse risk after withdrawal (31), our pediatric-specific estimate provides more targeted guidance for childhood epilepsy management. Furthermore, we found that the type of epilepsy included in the population was an important source of heterogeneity affecting the recurrence rate. The pooled recurrence rate for GGE was 47%. However, only two studies in this analysis exclusively recruited patients with genetic generalized epilepsy (GGE). Thus, the 47% relapse rate is not representative of the overall relapse rate for the GGE population. We may only conclude that GGE patients have a higher relapse rate than other groups, and this subgroup substantially impacts the pooled relapse rate of the total cohort.

### 4.2 Analysis of risk factors for seizure recurrence

This meta-analysis revealed that female gender is an independent risk factor for seizure recurrence following ASMs discontinuation in epileptic children, with female patients exhibiting a significantly higher recurrence risk than male counterparts. The univariable hazard ratio (HR) was 2.03, and the multivariable adjusted HR was slightly reduced to 1.97 after adjusting for confounding factors; the significant association persisted, confirming female sex as an independent risk factor rather than a confounder-driven outcome affected by age, seizure type and other covariates. To date, the biological mechanisms behind this sex-related discrepancy remain incompletely elucidated. Existing studies demonstrate that female hormone levels modulate neuronal excitability, and hormonal fluctuations during menstruation and puberty increase epileptic seizure susceptibility (32–34). This study enrolled children aged 0–18 years encompassing prepubertal girls and peripubertal female adolescents, and elevated relapse risk was also observed in prepubertal girls without obvious sex hormone fluctuations, indicating the hormonal hypothesis can only partially explain this phenomenon, and further basic studies are required to validate the underlying molecular mechanisms. Clinically, cautious ASMs tapering and discontinuation are warranted for female pediatric patients, especially peripubertal girls. Notably, the prognostic weight of female sex varies substantially across existing clinical researches, and discrepancies in the sex-associated relapse risk across published studies are mainly attributed to differences in study population composition, etiological spectrum and follow-up strategies. Consistent with the findings from Olmez et al. (17) in a mixed pediatric cohort with diverse etiologies and seizure types, multivariate Cox regression identified female sex as a major relapse risk factor, and Pavlović et al. (19) further validated its independent predictive value in the cryptogenic focal epilepsy subgroup, supporting its prognostic role in focal and mixed epilepsies. By contrast, Koken et al. (15) found no gender differences in a homogeneous cohort of idiopathic/genetic generalized epilepsy, as high etiological homogeneity diluted sex-specific effects, suggesting sex has limited prognostic weight in this epilepsy subtype. Though Yıldırım et al. (25) enrolled a mixed-etiology cohort, 72.5% of cases had idiopathic epilepsy and only 24.9% had symptomatic/structural epilepsy, and its median follow-up of 46 months was shorter than the maximum 172-month follow-up in Olmez et al.'s research, which may fail to capture long-term relapse events. By pooling heterogeneous data and improving statistical power, this meta-analysis confirmed the stable sex-related prognostic effect. Clinically, etiology-based stratified management is recommended: intensified follow-up and electroencephalography surveillance are necessary for girls with focal or cryptogenic epilepsy after ASMs discontinuation, while sex only serves as a secondary reference for children with pure idiopathic/genetic generalized epilepsy.

Moreover, focal seizure was verified as a strong independent predictor of post-discontinuation seizure recurrence. A review (35) conducted by Schmidt and Löscher on children and adults with epilepsy indicated that focal seizures constitute an important risk factor for epilepsy recurrence following ASMs discontinuation. Numerous studies (10, 35, 36) have indicated that individuals with focal seizures have a higher tendency toward recurrence following ASMs discontinuation. This factor is closely linked to the concept of “pharmacoresistance” early in the treatment course. Studies have shown that failure to achieve initial seizure control with the first well-tolerated ASMs is a strong predictor of subsequent intractability (37). It may be that the abnormal discharges in focal seizures are usually associated with structural lesions or functional abnormalities, whereas genetic etiologies are more common in generalized seizures and are less directly linked to local structural lesions. Therefore, compared with children with generalized seizures, clinicians should be more cautious when discontinuing medication in children with focal seizures and may consider extending the seizure-free observation period.

Apart from seizure type, disease background characteristics represented by symptomatic etiology and delayed development/intellectual disability also emerge as important risk factors for post-withdrawal seizure recurrence. Symptomatic etiology refers to epileptic seizures caused by identifiable abnormal brain structures or known acquired brain injuries. The mechanism by which it causes recurrence may be related to the persistent pathological basis (such as cerebral softening foci or residual tumors). The pathophysiological state induced by the etiology is prone to reactivation due to fluctuations in external factors. Population-based cohort studies have long indicated that children with remote symptomatic etiologies (e.g., cerebral palsy or significant cognitive impairment) have fundamentally different long-term prognoses compared to those with idiopathic epilepsies, with lower rates of sustained remission (38, 39). Berg and Shinnar's meta-analysis (31) found that individuals with symptomatic epilepsy face a 1.55-fold greater likelihood of seizure recurrence after discontinuing ASMs compared to those with idiopathic epilepsy. Clinical evidence of neurologic abnormalities may indicate damage to the integrity of neural pathways, as well as organic lesions in the brain or nerve tissues that affect the stability of neurological functions, thereby leading to recurrence. Meanwhile, multiple studies (20, 40) have indicated that individuals with intellectual disability are at an elevated risk of epileptic recurrence after discontinuing ASMs compared to those without intellectual disability. Such patients may have abnormalities in brain structure or function during neurodevelopment, resulting in reduced neural plasticity and impaired baseline regulatory capacity. They struggle to suppress abnormal discharges through their own compensatory mechanisms, ultimately increasing the risk of recurrence.

A history of febrile seizures was also associated with recurrence. Notably, prolonged febrile seizures (≥10 min), a key subtype of complex febrile seizures, have been confirmed to be closely associated with baseline developmental delay and potential brain injury in children, as such seizures may further impair the stability of neural circuits and reduce the brain's compensatory capacity by affecting early neurodevelopment (41). This may interfere with brain development at an early stage, reduce the stability of neural circuits, impair compensatory capacity, and lead to persistent long-term susceptibility, which together constitute the pathological basis for a heightened likelihood of recurrence after ASMs withdrawal.

Furthermore, abnormal EEG findings after ASM discontinuation were identified as an important predictor of seizure recurrence, which is consistent with the conclusions from a EEG-focused meta-analysis (42). Yao et al. reported that patients with abnormal EEG during ASM withdrawal exhibited a significantly higher recurrence rate compared with those with normal EEG, with the highest risk observed in individuals showing progressive EEG deterioration (42). Therefore, regular EEG monitoring is recommended throughout the ASM tapering and discontinuation process. If new or aggravated epileptiform discharges emerge, ASM withdrawal should be promptly suspended with subsequent re-evaluation.

### 4.3 Limitations of the study

The constraints of the present study are primarily manifested in three respects: First, all included studies were published in English, while non-English literature was excluded, leading to language selection bias. This limitation may fail to fully reflect the real-world research findings across regions worldwide and partially impair the generalizability of the present results. Second, although subgroup analyses and meta-regression were performed, only 34.46% of the high heterogeneity (*I*^2^ = 91.0%) could be explained. The remaining variance may be attributed to unmeasured confounding factors, such as medication tapering speed, the proportion of patients receiving monotherapy vs. polytherapy, and imaging abnormalities. Third, the exploration of underlying mechanisms remains limited. The biological mechanisms responsible for the elevated recurrence risk among female patients, particularly prepubertal girls, have not been fully elucidated. Further investigations combining basic experiments and cohort studies are therefore warranted.

## 5 Conclusion

Based on existing research data, this meta-analysis identifies the following as risk factors for recurrence after ASMs withdrawal in children with epilepsy: female gender, focal seizure, history of febrile seizures, delayed development/intellectual disability, symptomatic etiology, abnormal electroencephalogram (EEG) after withdrawal. The correlation between recurrence after withdrawal and factors such as family history of epilepsy, history of status epilepticus, age over 10 years at withdrawal, clinical evidence of neurologic abnormalities, seizures before treatment ≥2 remains undetermined. With the advancement of medical science and technology, higher-quality prospective studies are still needed to further explore the risk factors for recurrence in children with epilepsy after ASMs withdrawal. Future studies should adopt standardized, internationally harmonized protocols for defining remission, withdrawal indications, and taper schedules to reduce heterogeneity (43). This will allow more context-specific and personalized analyses, considering the potential risks and benefits of continued therapy, and thereby further inform clinical decisions regarding ASMs withdrawal and subsequent management. Several directions for future research can be proposed. First, based on the identified independent risk factors, multi-center clinical data can be integrated to establish a risk prediction model and a corresponding risk scoring scale for seizure recurrence after discontinuation of antiseizure medications in children with epilepsy. Clinically, this scale can be used to stratify patients by risk before drug withdrawal. For high-risk children, individualized management strategies can be implemented, such as prolonging the seizure-free observation period prior to drug tapering, slowing down the dose reduction rate, and increasing the frequency of electroencephalography monitoring. These measures facilitate precise drug withdrawal and stratified management, thereby reducing the risk of seizure recurrence. Second, future studies should conduct large-scale, prospective, multicenter cohort studies with unified diagnostic criteria for seizure recurrence and indications for antiseizure medication withdrawal. Potential covariates including medication withdrawal regimens, neuroimaging characteristics and combined antiseizure therapy should be systematically recorded to further validate and refine the risk factors identified in the present study. Third, subsequent studies may expand the literature search scope and incorporate publications in different languages, grey literature, and unpublished negative outcome studies to further validate the conclusions. Fourth, future studies should examine the long-term psychosocial consequences and quality of life of children undergoing drug withdrawal, regardless of whether seizure recurrence occurs, in order to comprehensively evaluate the benefits and potential disadvantages of discontinuation (44). Moreover, particular attention should be given to individuals who experience severe or intractable recurrence after withdrawal, as this subgroup may face the greatest clinical burden, and identifying predictors of such outcomes will be of particular importance (45).

## Data Availability

The original contributions presented in the study are included in the article/Supplementary Material, further inquiries can be directed to the corresponding author.
