# Supplementary material for: Recurrence rate and risk factors of recurrence after antiseizure medications withdrawal in children with epilepsy: a meta-analysis
Source: Front Pediatr. 2026 Jul 27;14:1827056. doi: 10.3389/fped.2026.1827056 (PMC13454394; doi:10.3389/fped.2026.1827056)
Supplement: Supplementary file 1 [file Datasheet1.pdf]

Supplementary Material

Table S1 The detailed search process

Database	Searching number	Query	Results
Pubmed	1	Child[MeSH Terms]	2,264,718
	2	(Child[Title/Abstract]) OR (Children[Title/Abstract])	1,637,816
	3	#1 OR #2	2,784,719
	4	Epilepsy[MeSH Terms]	132,979
	5	((((((((((((Epilepsy[Title/Abstract]) OR (Epilepsies[Title/Abstract])) OR (Seizure Disorder[Title/Abstract])) OR (Seizure Disorders[Title/Abstract])) OR (Epilepsy, Cryptogenic[Title/Abstract])) OR (Cryptogenic Epilepsies[Title/Abstract])) OR (Cryptogenic Epilepsy[Title/Abstract])) OR (Epilepsies, Cryptogenic[Title/Abstract])) OR (Aura[Title/Abstract])) OR (Auras[Title/Abstract])) OR (Awakening Epilepsy[Title/Abstract])) OR (Epilepsy, Awakening[Title/Abstract])) OR (Seizure[Title/Abstract])	191,488
	6	#4 OR #5	223,569
	7	Recurrence[MeSH Terms]	209,007
	8	(((((Recurrence[Title/Abstract]) OR	602,182

	(Recurrences[Title/Abstract])) OR (Relapse[Title/Abstract])) OR (Relapses[Title/Abstract])) OR (Recrudescence[Title/Abstract])) OR (Recrudescences[Title/Abstract])	
9	#7 OR #8	724,012
10	Risk Factors[MeSH Terms]	1,035,526
11	((((((((((((((((Risk Factors[Title/Abstract]) OR (Factor, Risk[Title/Abstract])) OR (Risk Factor[Title/Abstract])) OR (Population at Risk[Title/Abstract])) OR (Populations at Risk[Title/Abstract])) OR (Risk Scores[Title/Abstract])) OR (Risk Score[Title/Abstract])) OR (Score, Risk[Title/Abstract])) OR (Risk Factor Scores[Title/Abstract])) OR (Risk Factor Score[Title/Abstract])) OR (Score, Risk Factor[Title/Abstract])) OR (Health Correlates[Title/Abstract])) OR (Correlates, Health[Title/Abstract])) OR (Social Risk Factors[Title/Abstract])) OR (Factor, Social Risk[Title/Abstract])) OR (Factors, Social Risk[Title/Abstract])) OR (Risk Factor, Social[Title/Abstract])) OR (Risk Factors, Social[Title/Abstract])) OR (Social Risk Factor[Title/Abstract])	923,224

	12	#10 OR #11	1,551,803
	13	#3 AND #6 AND #9 AND #12	441
Embase	#51	#4 AND #19 AND #28 AND #50	696
	#50	#29 OR #30 OR #31 OR #32 OR #33 OR #34 OR #35 OR #36 OR #37 OR #38 OR #39 OR #40 OR #41 OR #42 OR #43 OR #44 OR #45 OR #46 OR #47 OR #48 OR #49	2033334
	#49	'social risk factor':ab,ti	143
	#48	'risk factors, social':ab,ti	162
	#47	'risk factor, social':ab,ti	9
	#46	'factors, social risk':ab,ti	10
	#45	'factor, social risk':ab,ti	0
	#44	'social risk factors':ab,ti	1591
	#43	'correlates, health':ab,ti	13
	#42	'health correlates':ab,ti	687
	#41	'score, risk factor':ab,ti	17
	#40	'risk factor score':ab,ti	310
	#39	'risk factor scores':ab,ti	156
	#38	'score, risk':ab,ti	1445
	#37	'risk score':ab,ti	55986
	#36	'risk scores':ab,ti	26935
	#35	'populations at risk':ab,ti	3997
	#34	'population at risk':ab,ti	6341
	#33	'risk factor':ab,ti	441725

#32	'factor, risk':ab,ti	373
#31	'risk factors':ab,ti	956558
#30	'risk factor':ab,ti	441725
#29	'risk factor'/exp	1544414
#28	#20 OR #21 OR #22 OR #23 OR #24 OR #25 OR #26 OR #27	1086575
#27	'recrudescences':ab,ti	271
#26	'recrudescence':ab,ti	4029
#25	'relapses':ab,ti	60371
#24	'relapse':ab,ti	283801
#23	'recurrences':ab,ti	92645
#22	'recurrence':ab,ti	619978
#21	'recurrent disease':ab,ti	25691
#20	'recurrent disease'/exp	243123
#19	#5 OR #6 OR #7 OR #8 OR #9 OR #10 OR #11 OR #12 OR #13 OR #14 OR #15 OR #16 OR #17 OR #18	396583
#18	'seizure':ab,ti	130903
#17	'epilepsy, awakening':ab,ti	3
#16	'awakening epilepsy':ab,ti	18
#15	'auras':ab,ti	1426
#14	'aura':ab,ti	13275
#13	'epilepsies, cryptogenic':ab,ti	4
#12	'cryptogenic epilepsy':ab,ti	427

	#11	'cryptogenic epilepsies':ab,ti	79
	#10	'epilepsy, cryptogenic':ab,ti	17
	#9	'seizure disorders':ab,ti	3075
	#8	'seizure disorder':ab,ti	4132
	#7	'epilepsies':ab,ti	12872
	#6	'epilepsy':ab,ti	199126
	#5	'epilepsy'/exp	322214
	#4	#1 OR #2 OR #3	4134894
	#3	'children':ab,ti	1832114
	#2	'child':ab,ti	612141
	#1	'child'/exp	3642832
Cochrane library	#1	MeSH descriptor: [Child] explode all trees	81919
	#2	(Child):ti,ab,kw OR (Children):ti,ab,kw	196753
	#3	#1 OR #2	196753
	#4	MeSH descriptor: [Epilepsy] explode all trees	3510
	#5	(Epilepsy):ti,ab,kw OR (Epilepsies):ti,ab,kw OR (Seizure Disorder):ti,ab,kw OR (Seizure Disorders):ti,ab,kw OR (Epilepsy, Cryptogenic):ti,ab,kw	10438
	#6	(Cryptogenic Epilepsies):ti,ab,kw OR (Cryptogenic Epilepsy):ti,ab,kw OR (Epilepsies, Cryptogenic):ti,ab,kw OR (Aura):ti,ab,kw OR (Auras):ti,ab,kw	2184
	#7	(Awakening Epilepsy):ti,ab,kw OR (Epilepsy,	7834

		Awakening):ti,ab,kw OR (Seizure):ti,ab,kw	
	#8	#4 OR #5 OR #6 OR #7	15325
	#9	MeSH descriptor: [Recurrence] explode all trees	16448
	#10	(Recurrence):ti,ab,kw OR (Recurrences):ti,ab,kw OR (Relapse):ti,ab,kw OR (Relapses):ti,ab,kw OR (Recrudescence):ti,ab,kw	93721
	#11	(Recrudescences):ti,ab,kw	73
	#12	#9 OR #10 OR #11	93941
	#13	MeSH descriptor: [Risk Factors] explode all trees	37757
	#14	(Risk Factors):ti,ab,kw OR (Factor, Risk):ti,ab,kw OR (Risk Factor):ti,ab,kw OR (Population at Risk):ti,ab,kw OR (Populations at Risk):ti,ab,kw	160633
	#15	(Risk Scores):ti,ab,kw OR (Risk Score):ti,ab,kw OR (Score, Risk):ti,ab,kw OR (Risk Factor Scores):ti,ab,kw OR (Risk Factor Score):ti,ab,kw	56912
	#16	(Score, Risk Factor):ti,ab,kw OR (Health Correlates):ti,ab,kw OR (Correlates, Health):ti,ab,kw OR (Social Risk Factors):ti,ab,kw OR (Factor, Social Risk):ti,ab,kw	16677
	#17	(Factors, Social Risk):ti,ab,kw OR (Risk Factor, Social):ti,ab,kw OR (Risk Factors, Social):ti,ab,kw OR (Social Risk Factor):ti,ab,kw	7518
	#18	#13 OR #14 OR #15 OR #16 OR #17	189907
	#19	#3 AND #8 AND #12 AND #18	76
Web of science	#1	TS=(Child) OR TS=(Children)	2270944

#2	TS=(Epilepsy) OR TS=(Epilepsies) OR TS=(Seizure Disorder) OR TS=(Seizure Disorders) OR TS=(Epilepsy, Cryptogenic) OR TS=(Cryptogenic Epilepsies) OR TS=(Cryptogenic Epilepsy) OR TS=(Epilepsies, Cryptogenic) OR TS=(Aura) OR TS=(Auras) OR TS=(Awakening Epilepsy) OR TS=(Epilepsy, Awakening) OR TS=(Seizure)	297908
#3	TS=(Recurrence) OR TS=(Recurrences) OR TS=(Relapse) OR TS=(Relapses) OR TS=(Recrudescence) OR TS=(Recrudescences)	722721
#4	TS=(Risk Factors) OR TS=(Factor, Risk) OR TS=(Risk Factor) OR TS=(Population at Risk) OR TS=(Populations at Risk) OR TS=(Risk Scores) OR TS=(Risk Score) OR TS=(Score, Risk) OR TS=(Risk Factor Scores) OR TS=(Risk Factor Score) OR TS=(Score, Risk Factor) OR TS=(Health Correlates) OR TS=(Correlates, Health) OR TS=(Social Risk Factors) OR TS=(Factor, Social Risk) OR TS=(Factors, Social Risk) OR TS=(Risk Factor, Social) OR TS=(Risk Factors, Social) OR TS=(Social Risk Factor)	2378664
#5	#4 AND #3 AND #2 AND #1	599

Table S2 NOS scores (1)

Study	Is the case definition adequate?	Representativeness of the cases	Selection of controls	Definition of Controls	Comparability of cases and controls based on the design or analysis	Ascertainment of exposure	Same method of ascertainment for cases and controls	Non-Response rate	Total scores
Triono,A 2024	*	*	*	*	*	*	*	*	8

Table S3 NOS scores (2)

Study	Representativeness of the exposed cohort	Selection of the non-exposed cohort	Ascertainment of exposure	Identification of outcome indicators not yet to be observed at study entry	Comparability of cohorts on the basis of the design or statistical analysis	Assessment of outcome	Was follow-up long enough for outcomes to occur	Adequacy of follow-up of cohorts	Total scores
Altunbaşak,S 1999	*	*	*	*	*	*	*	*	8
Caviedes,B.E 1998	*	*	*	*	*	*	*	*	8
Dooley,J 1996	*	*	*	*	*	*	*	*	8
Gebremariam,A	*	*	*	/	*	*	*	*	7

1999									
Kanmaz,S 2023	*	*	*	*	*	*	*	*	8
Karalok,Z.S 2020	*	*	*	*	*	*	*	*	8
Koken,O.Y 2021	*	*	*	*	*	*	*	*	8
Ohta,H 2004	*	*	*	*	*	*	*	*	8
Olmez,A 2009	*	*	*	*	*	*	*	*	8
Pavlović,M 2012	*	*	*	*	*	*	*	*	8
Ramos-Lizana,J 2010	*	*	*	*	*	*	*	*	8
Shinnar,S 1994	*	*	*	*	*	*	*	*	8
Verrotti,A 2012	*	*	*	*	*	*	*	*	8
Vurucu,S 2010	*	*	*	*	*	*	*	*	8
Yildirim,M 2022	*	*	*	*	*	*	*	*	8
Zhao,Y 2023	*	*	*	*	**	*	*	*	9
Srivastava,K	*	*	*	*	*	*	*	*	8

2017									
Pavlović,M 2011	*	*	*	*	*	*	*	*	8

Table S4 Single factor meta-analysis

Risk factors	No of study	heterogeneity		OR (95%CI)	HR (95%CI)	P	Egger
		I^2(%)	P				
Female gender	3	54.6	0.110	-	2.03 (1.16, 3.56)	0.014	0.187
Focal seizure	4	24.9	0.262	1.36 (1.15, 1.60)	-	0.000	0.867
Family history of epilepsy	3	0.0	0.880	-	1.46 (0.80, 2.66)	0.217	0.996
History of febrile seizure	4	0.0	0.643	1.64 (1.25, 2.16)	-	0.000	0.279
History of status epilepticus	3	0.0	0.862	1.18 (0.66, 2.10)	-	0.582	0.983
Age at withdrawal > 10 y	3	83.9	0.002	1.94 (0.73, 5.14)	-	0.185	0.572
Delayed development/intellectual disability	3	33.8	0.221	1.78 (1.33, 2.39)	-	0.000	0.479
Clinical evidence of neurologic abnormalities	4	56.3	0.077	0.05 (-0.44, 0.55)	-	0.083	0.370
Seizures before treatment ≥2	3	57.0	0.098	0.81 (0.54, 1.21)	-	0.297	0.180
Symptomatic etiology	4	35.9	0.197	1.60 (1.25, 2.04)	-	0.000	0.308

Table S5 Multi-factor meta-analysis

Risk factors	No of study	heterogeneity		OR (95%CI)	HR (95%CI)	P	Egger
		I^2(%)	P				
Female gender	5	35.3	0.186	-	1.97 (1.41, 2.76)	0.000	0.542
Abnormal EEG after withdrawal	3	0.0	0.596	3.11 (1.85, 5.24)	-	0.000	0.445
Family history of epilepsy	3	0.0	0.811	-	1.26 (0.70, 2.26)	0.445	0.972
History of febrile seizure	3	0.0	0.941	-	2.21 (1.24, 3.95	0.007	0.820
Delayed development/intellectual disability	3	0.0	0.755	1.86 (1.25, 2.75)	-	0.000	0.487

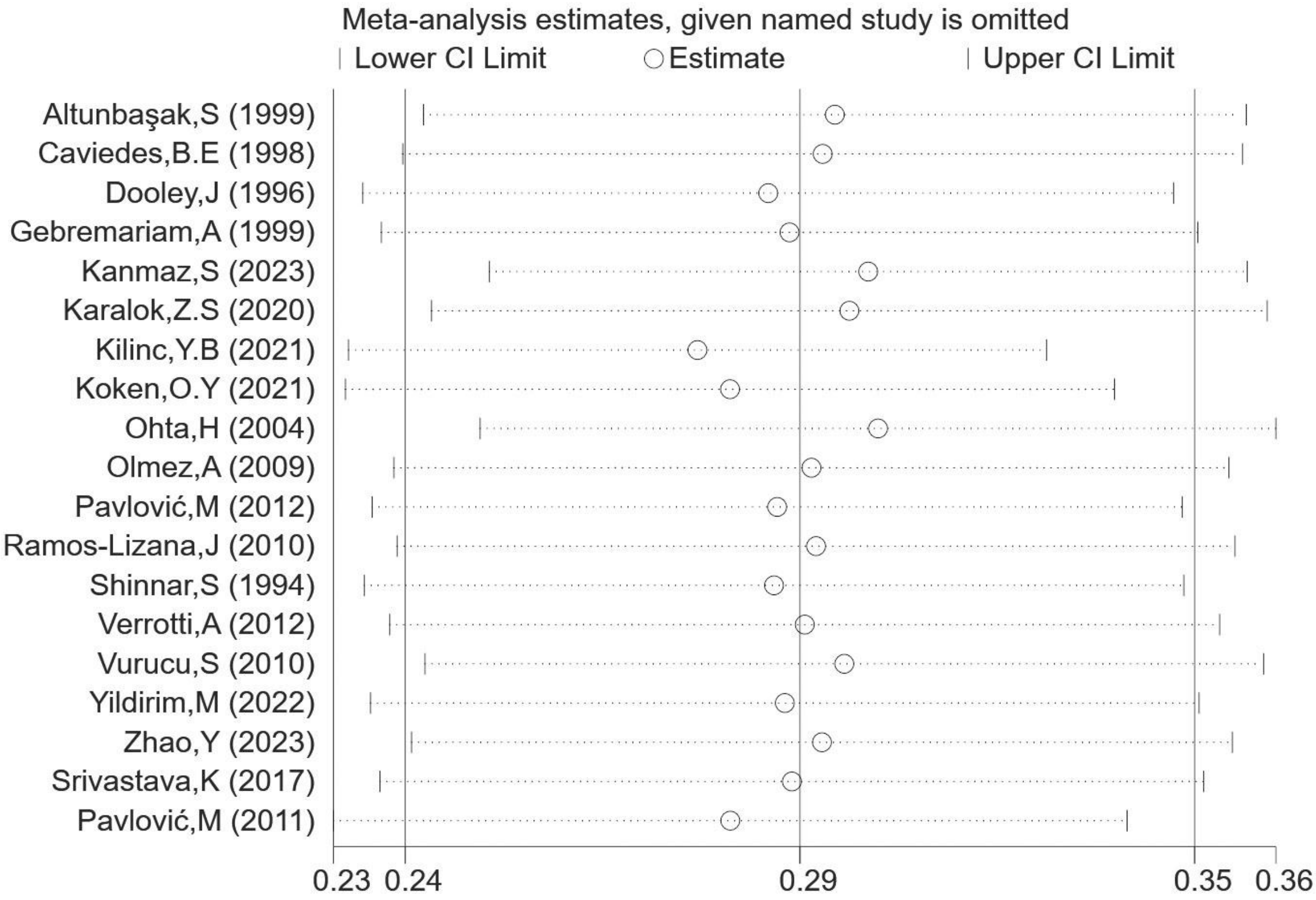

Figure S1 Sensitivity analysis of recurrence rate

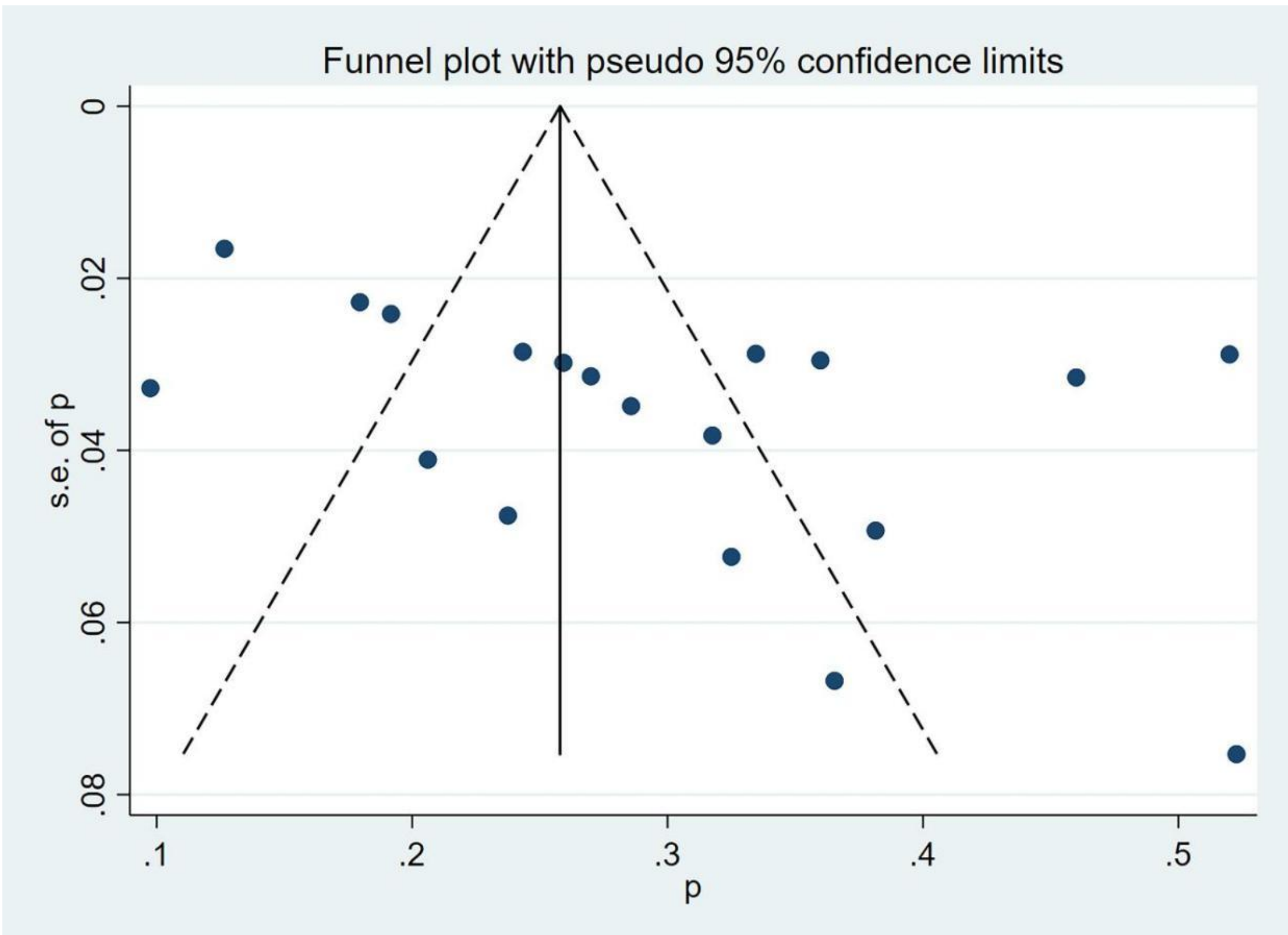

Figure S2 Funnel plot of recurrence rate

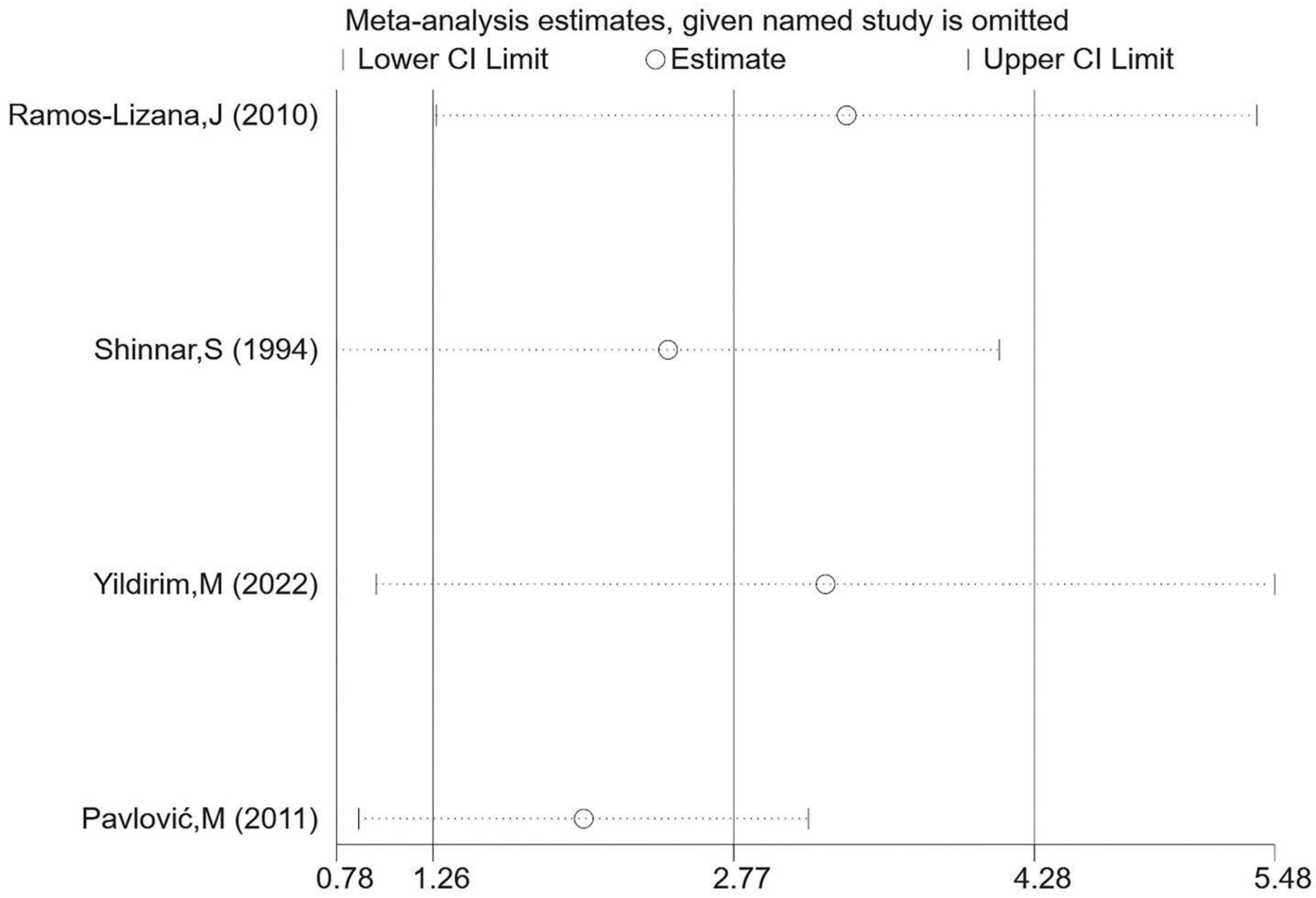

Figure S3 Sensitivity analysis of age of seizure onset≥10y (Single factor)

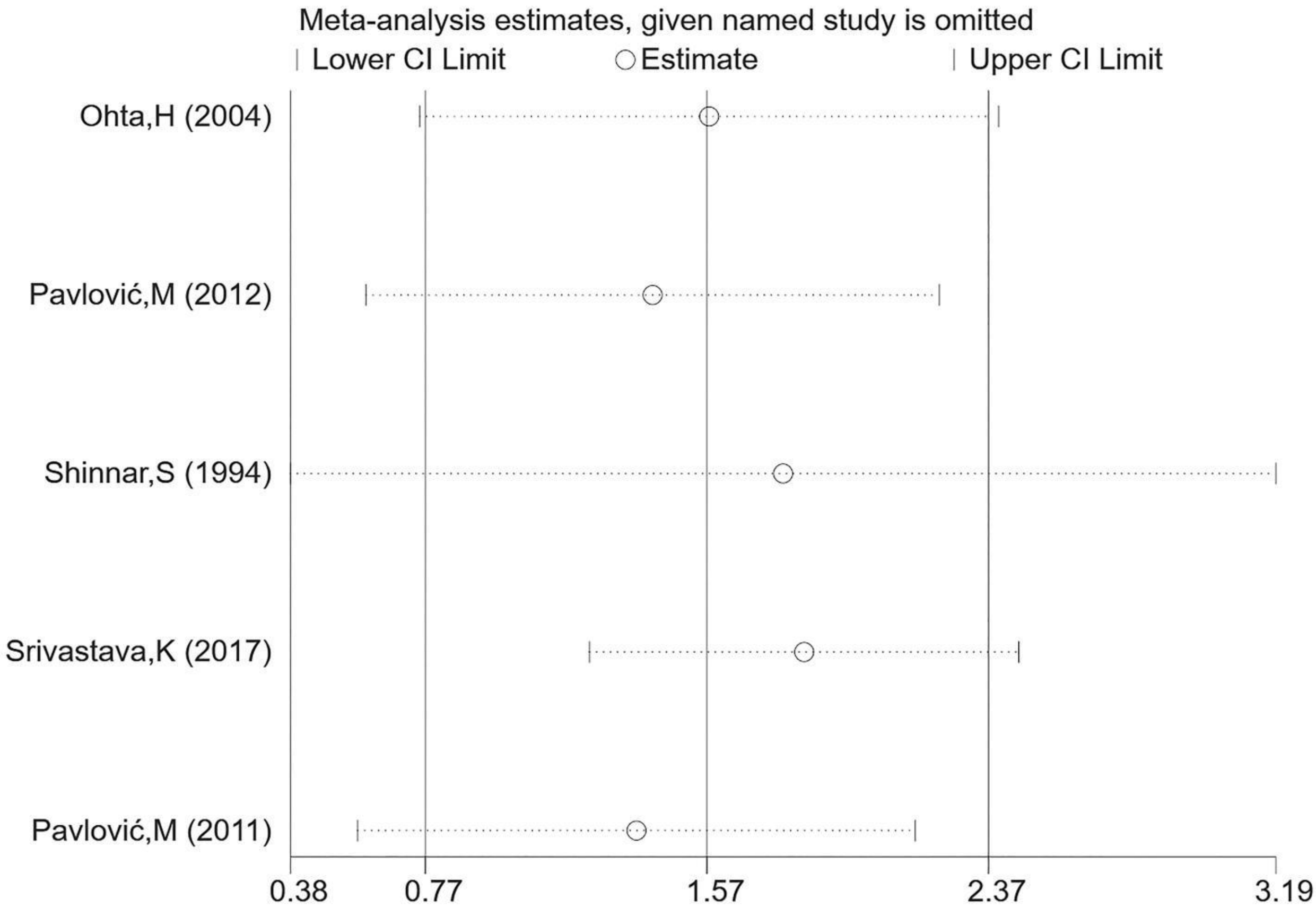

Figure S4 Sensitivity analysis of age at withdrawal＞10y (Single factor)

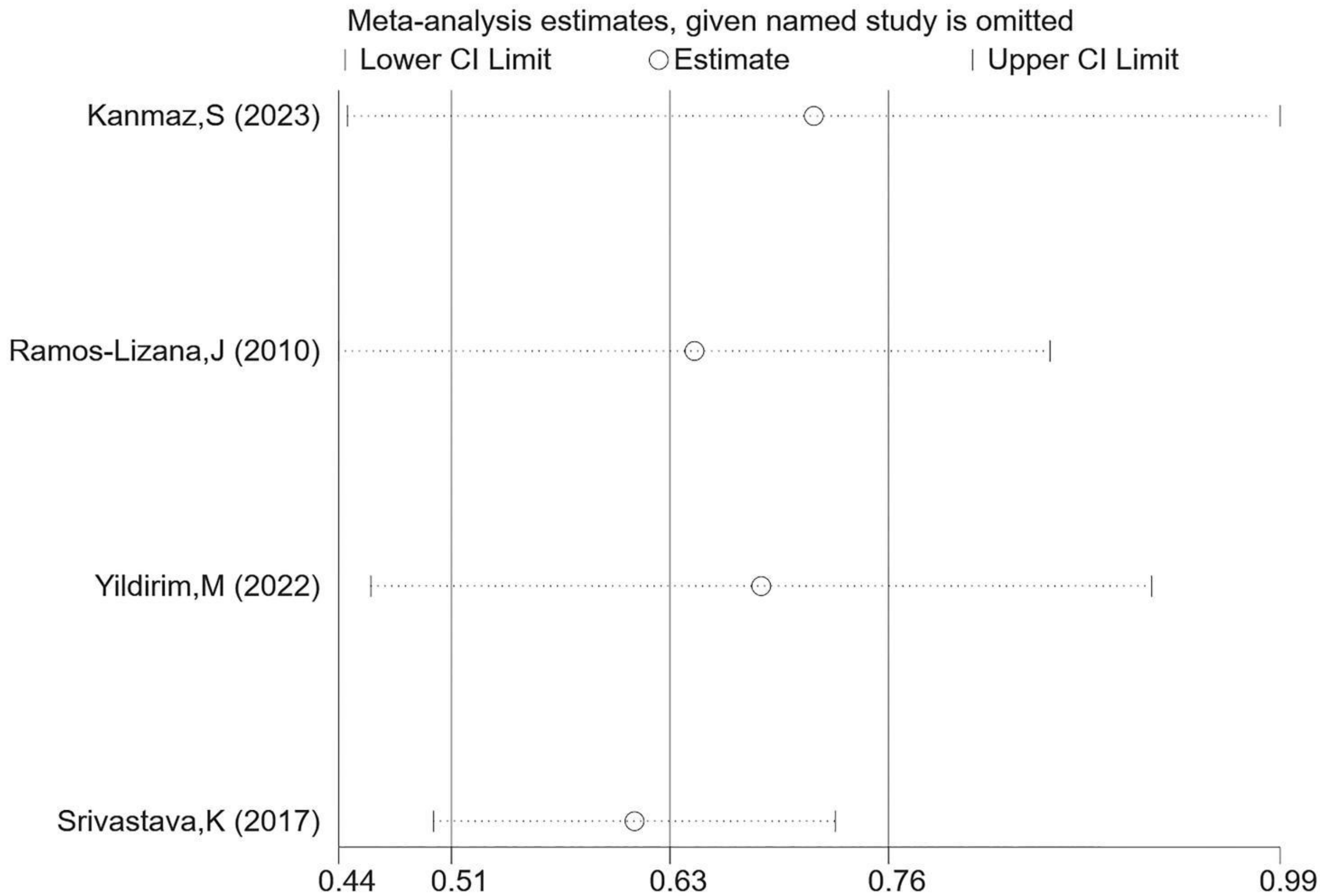

Figure S5 Sensitivity analysis of withdrawal period ≥4months (Single factor)

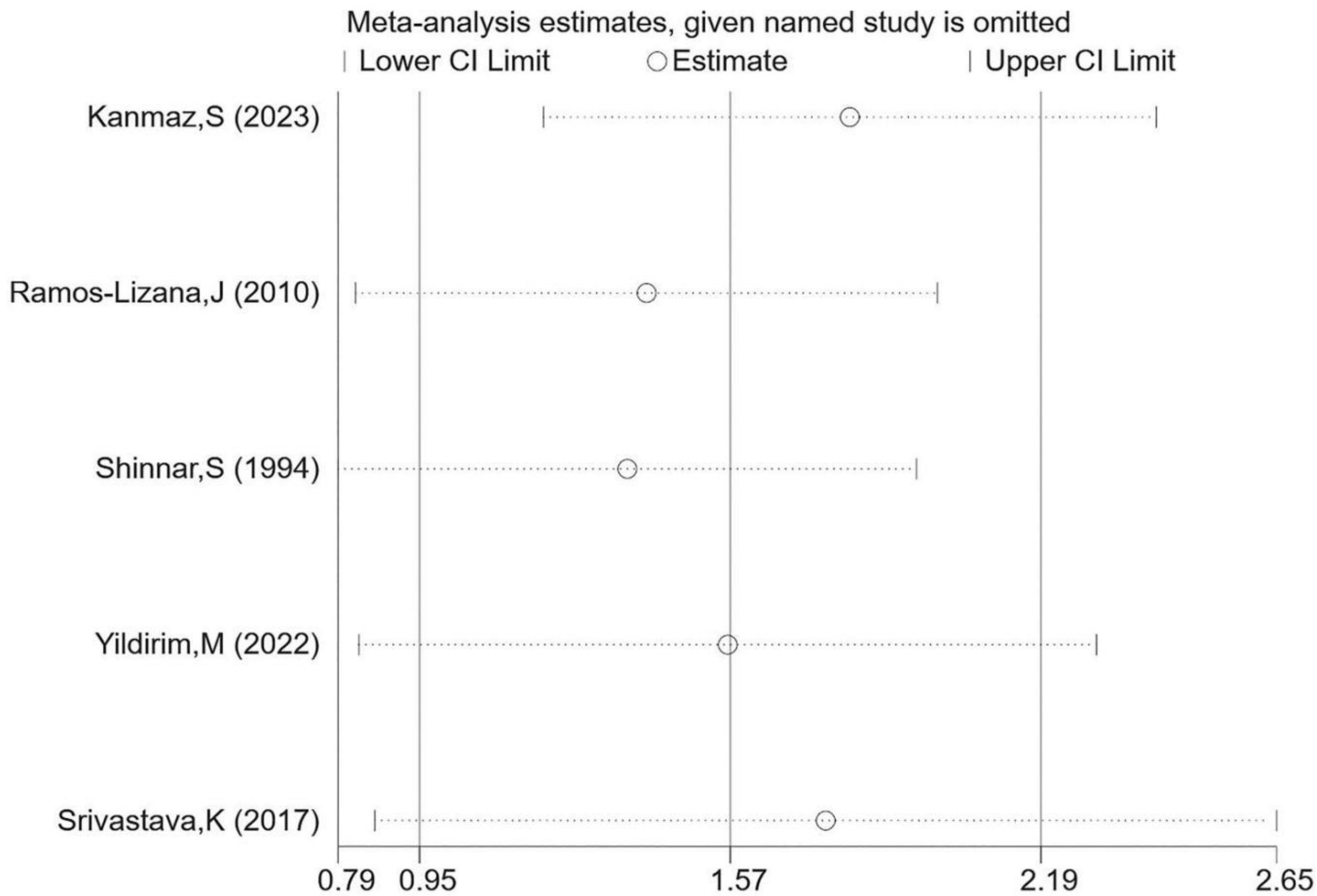

Figure S6 Sensitivity analysis of delayed development or intellectual disability (Single factor)

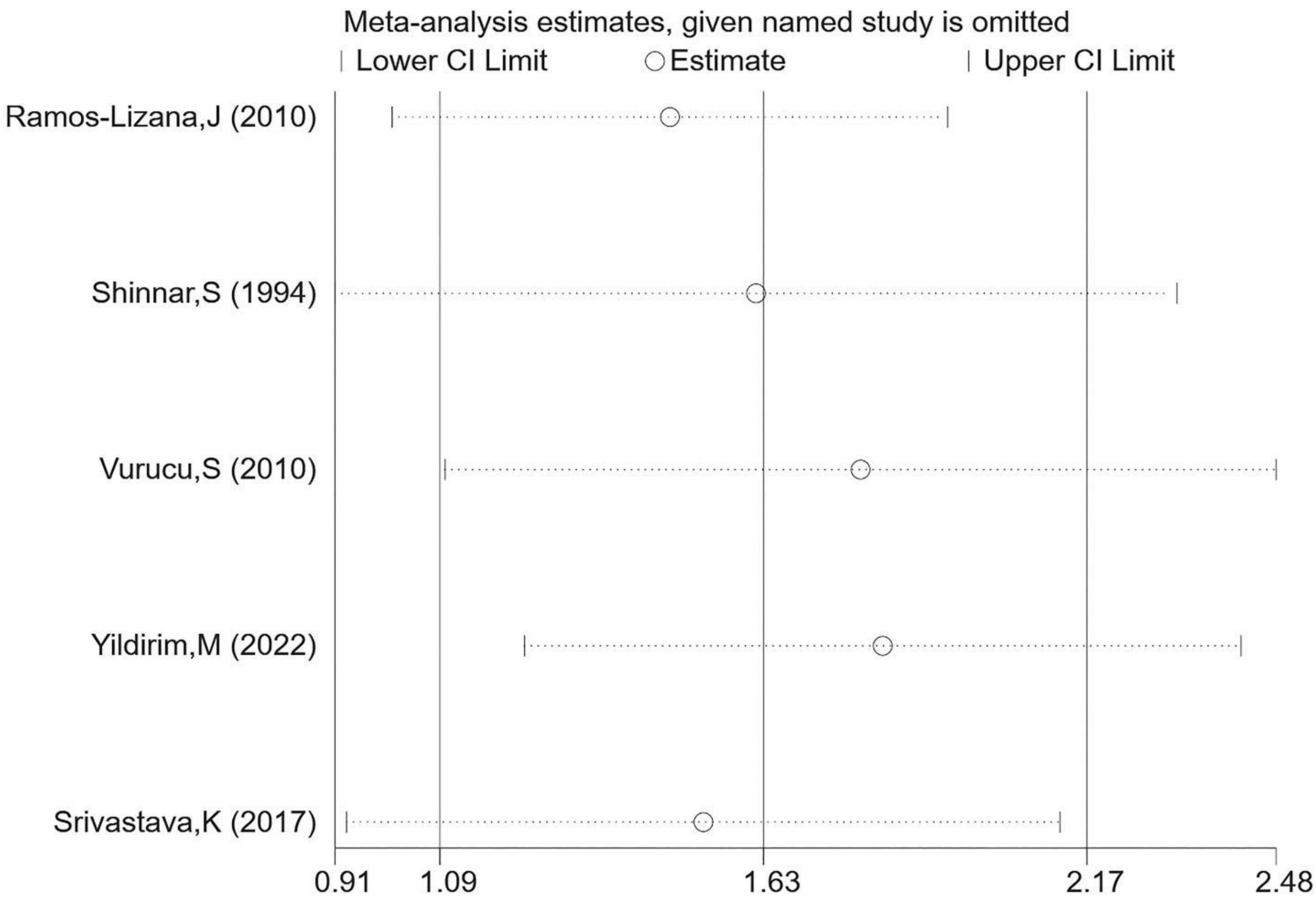

Figure S7 Sensitivity analysis of symptomatic etiology (Single factor)

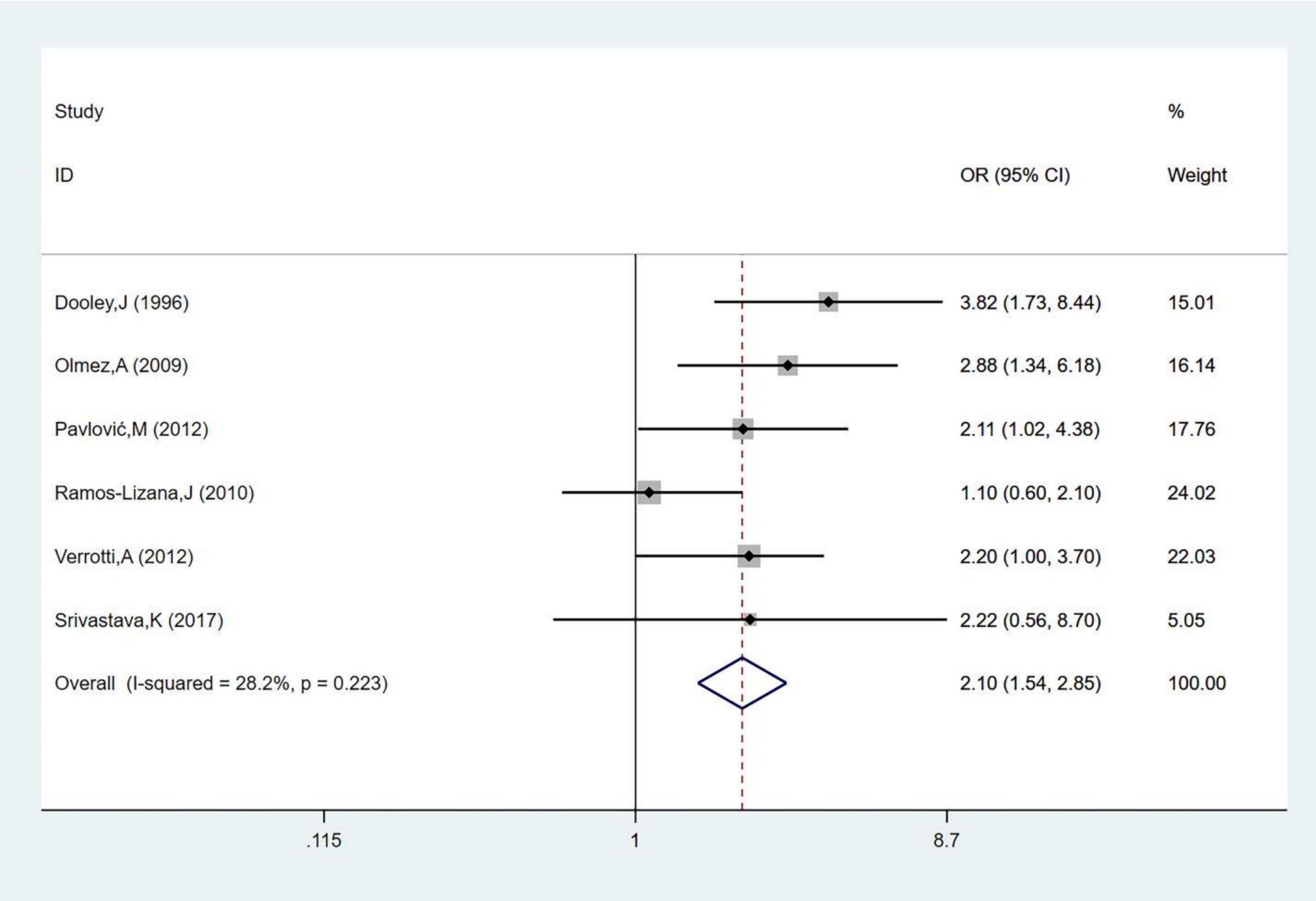

Figure S8 Forest plot of female gender (Multi-factor)

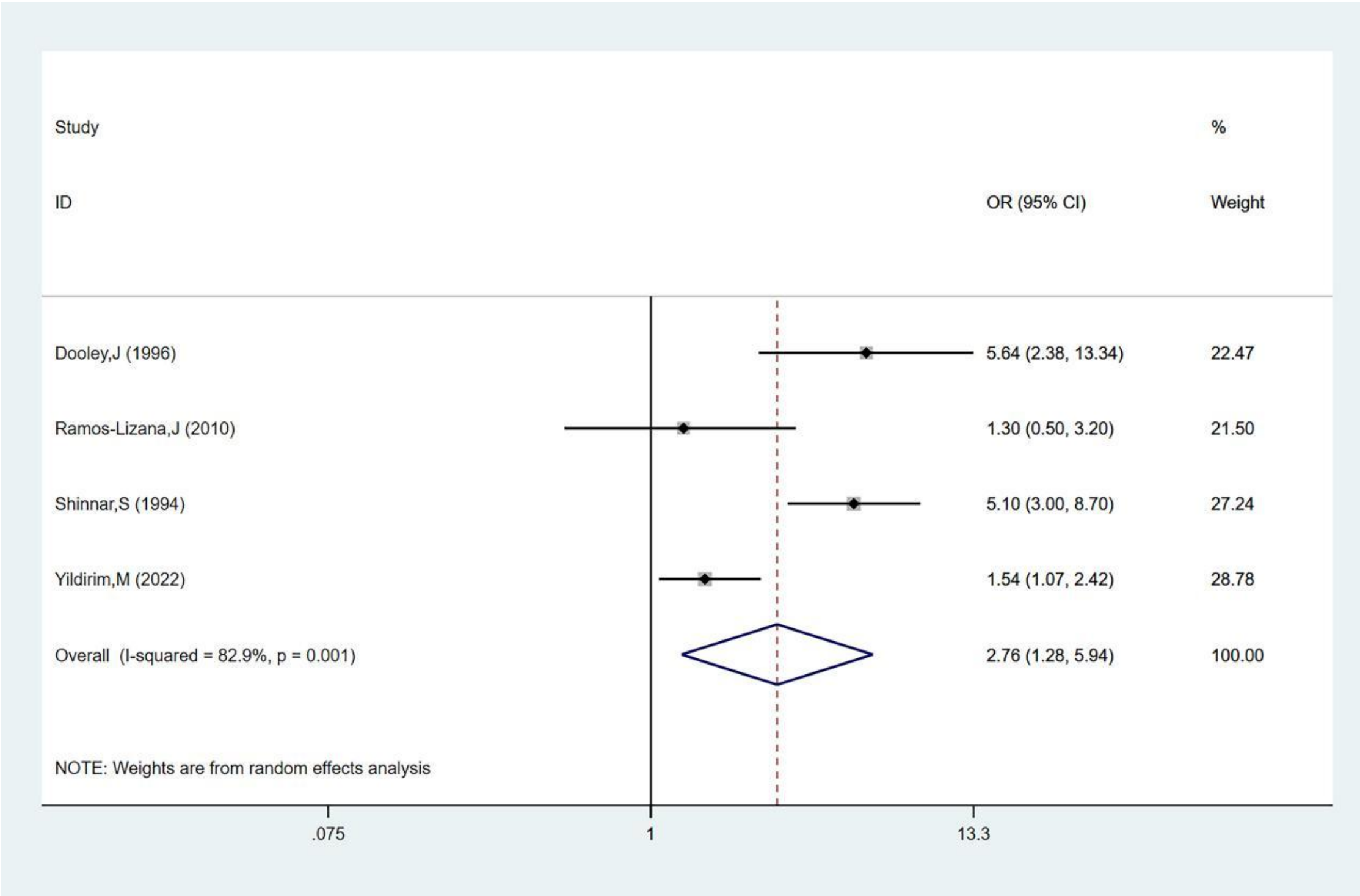

Figure S9 Forest plot of age of seizure onset≥10y (Multi-factor)

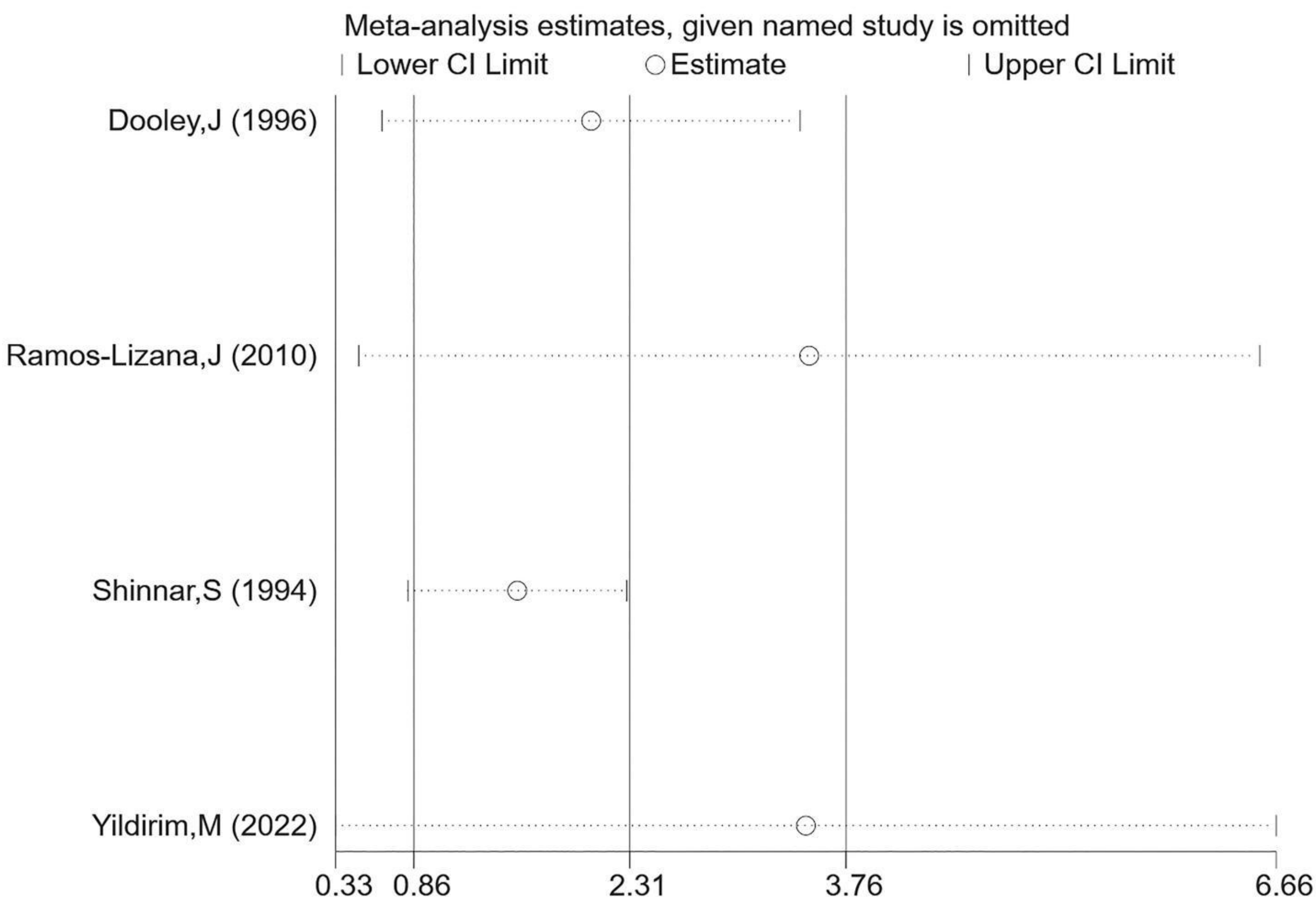

Figure S10 Sensitivity analysis of age of seizure onset≥10y (Multi-factor)

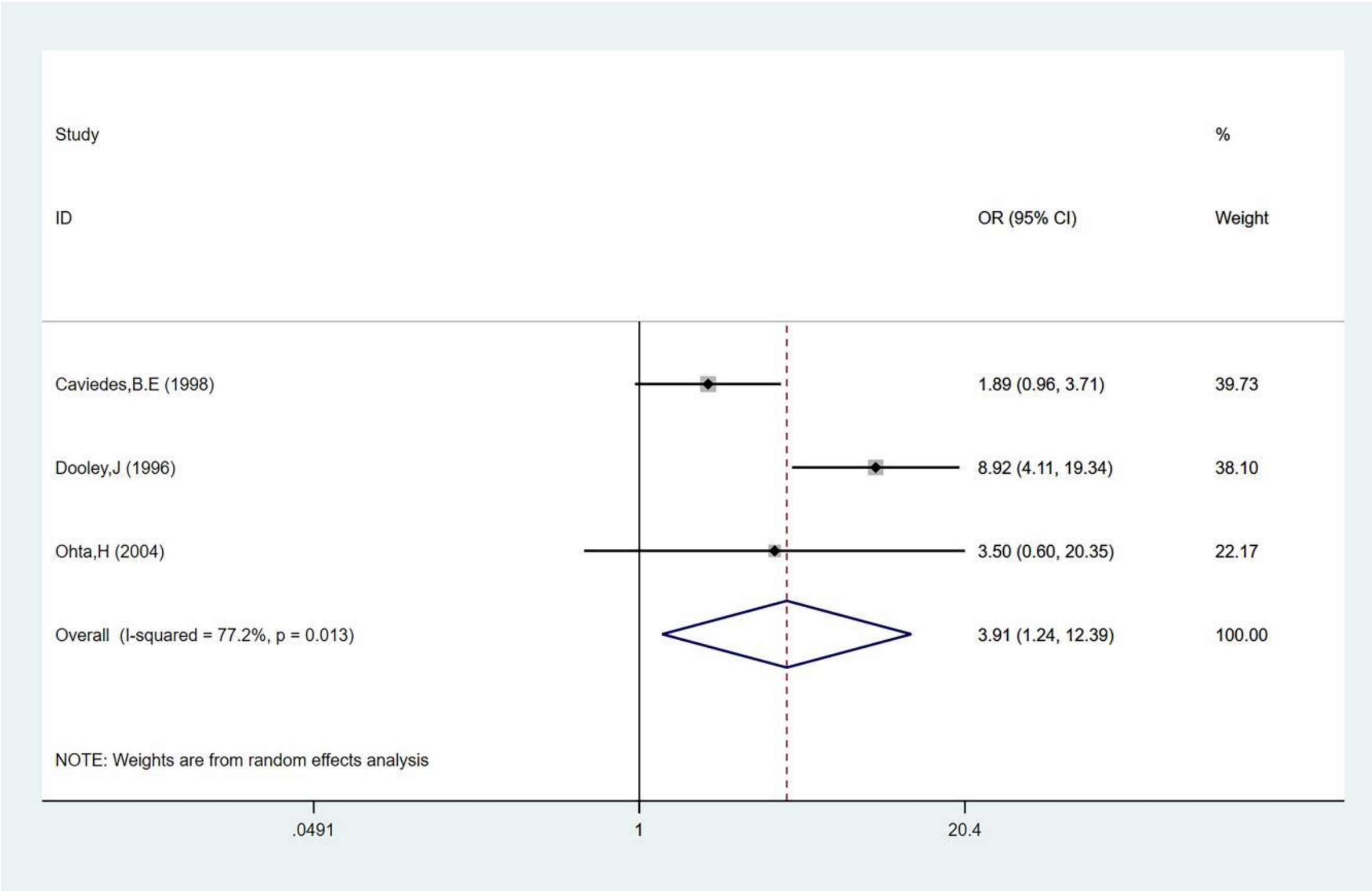

Figure S11 Forest plot of age of focal seizure (Multi-factor)

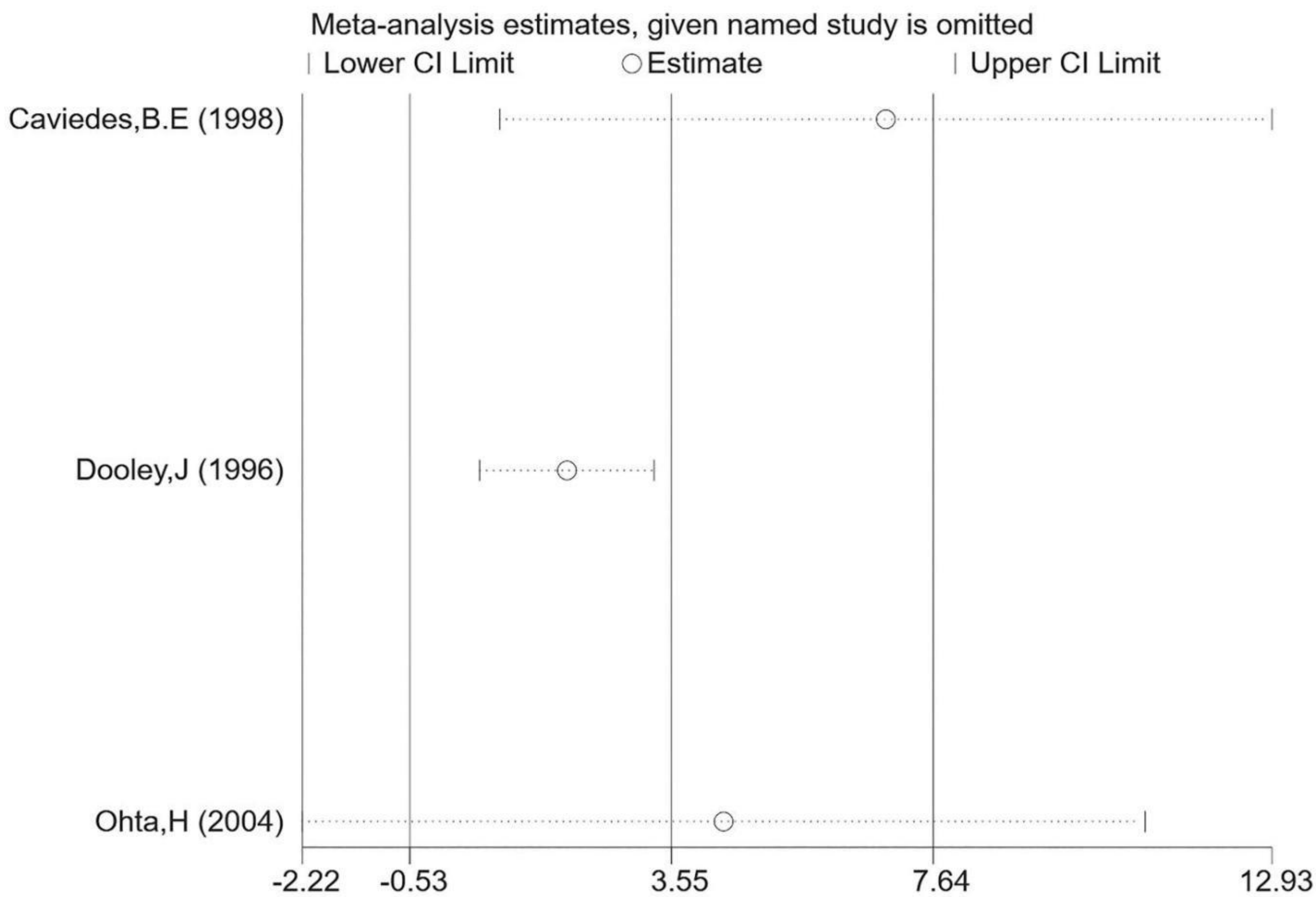

Figure S12 Sensitivity analysis of focal seizure (Multi-factor)

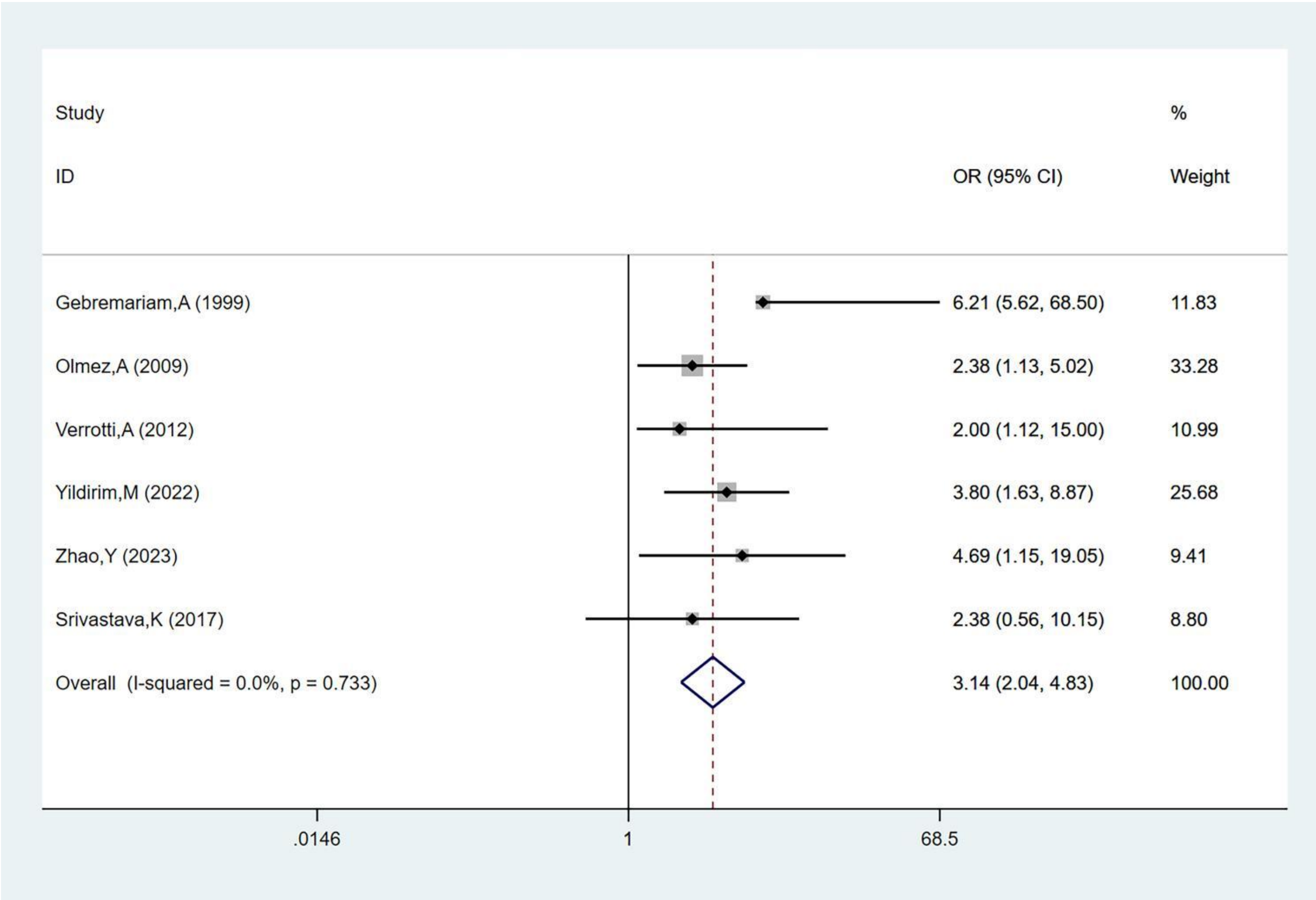

Figure S13 Forest plot of abnormal EEG after withdrawal (Multi-factor)

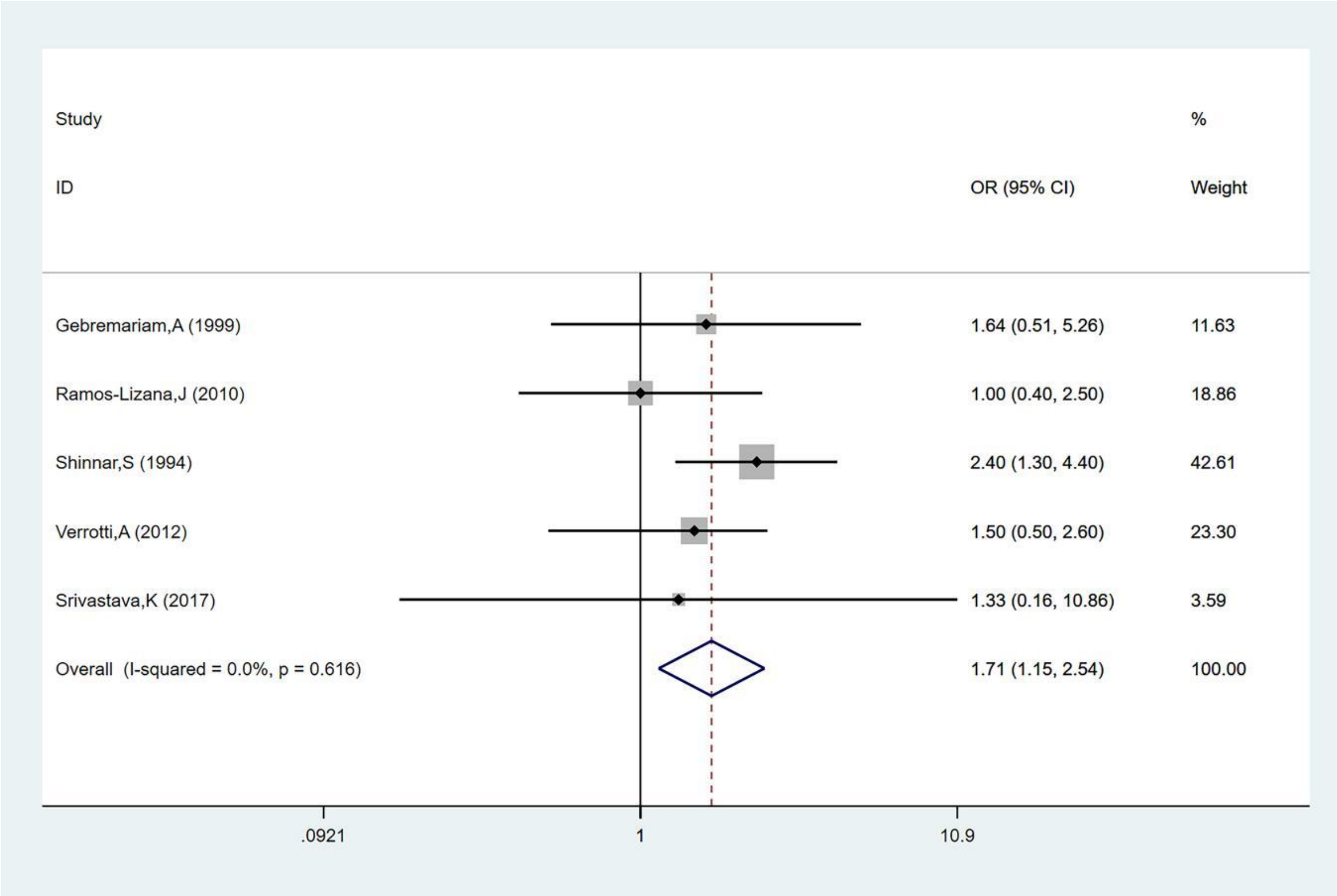

Figure S14 Forest plot of family history of epilepsy (Multi-factor)

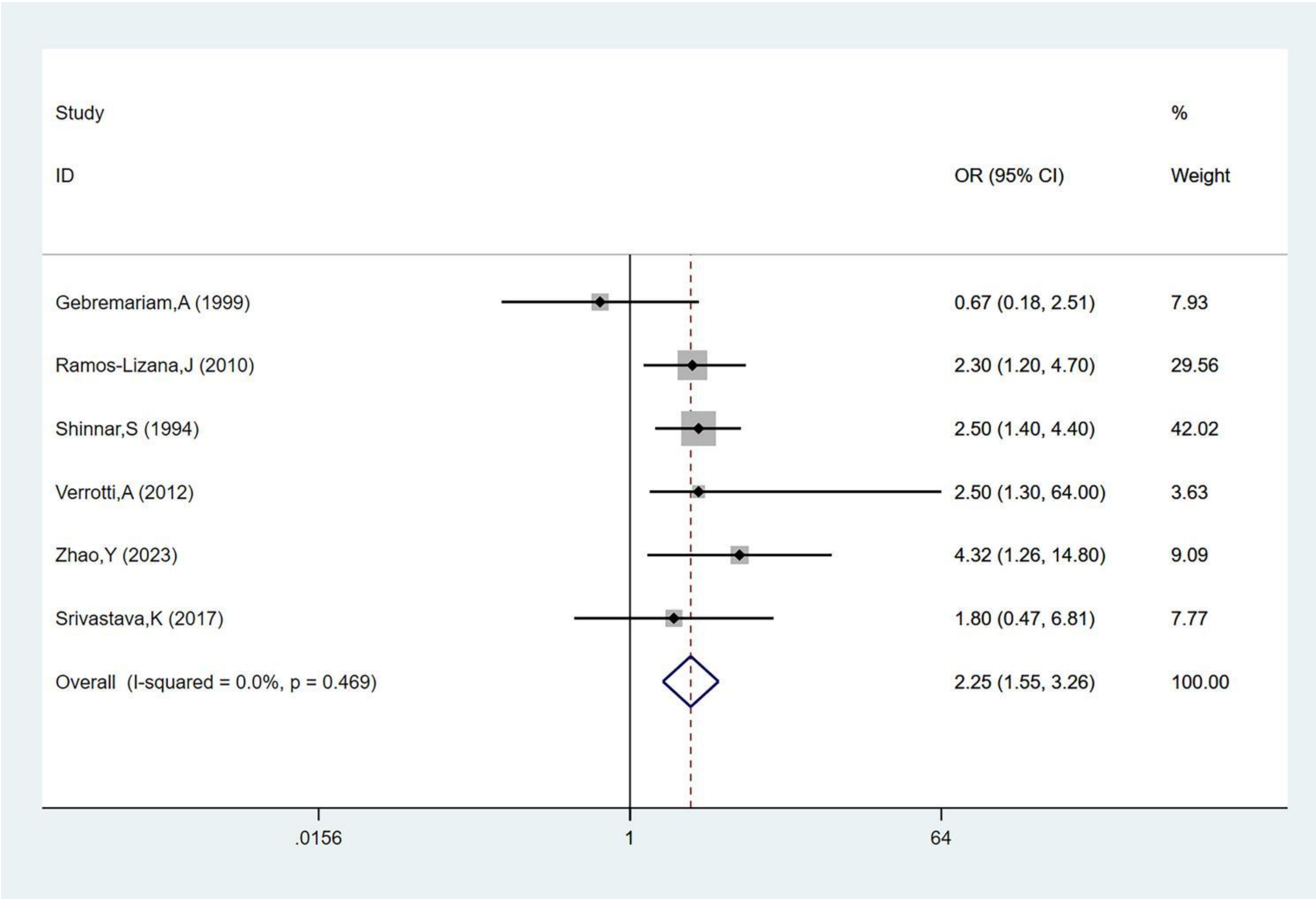

Figure S15 Forest plot of history of febrile seizure (Multi-factor)

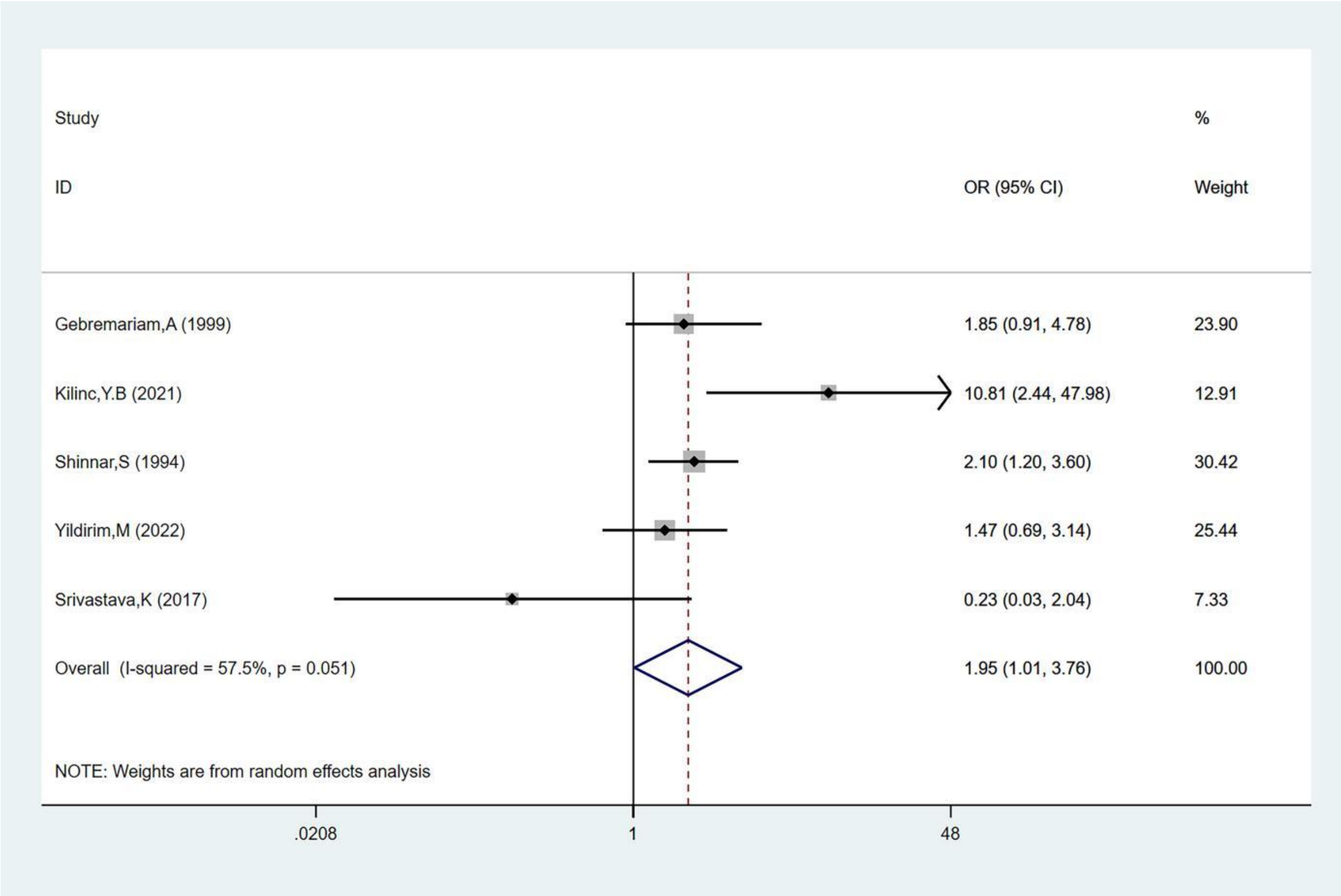

Figure S16 Forest plot of delayed development or intellectual disability (Multi-factor)

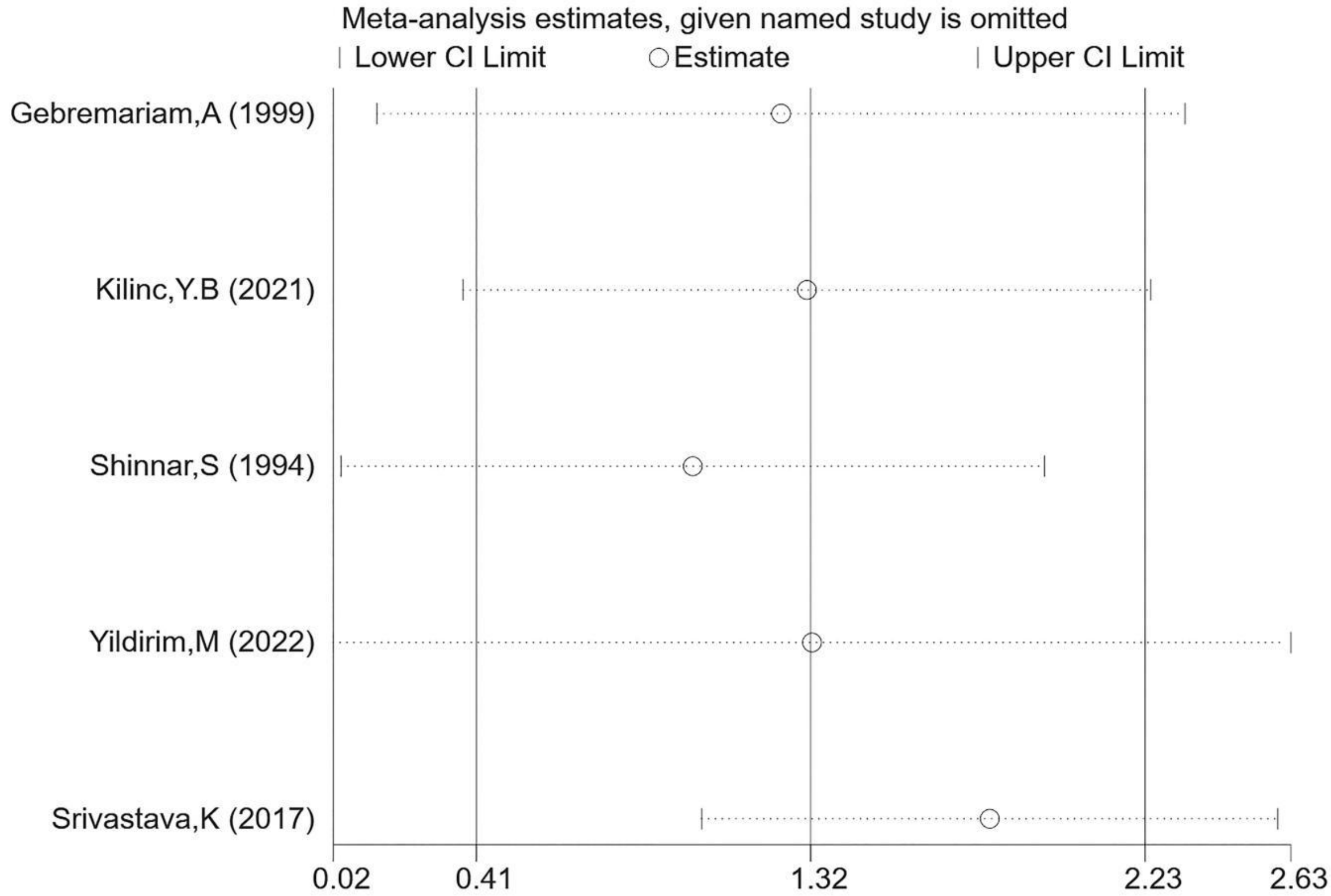

Figure S17 Sensitivity analysis of delayed development or intellectual disability (Multi-factor)

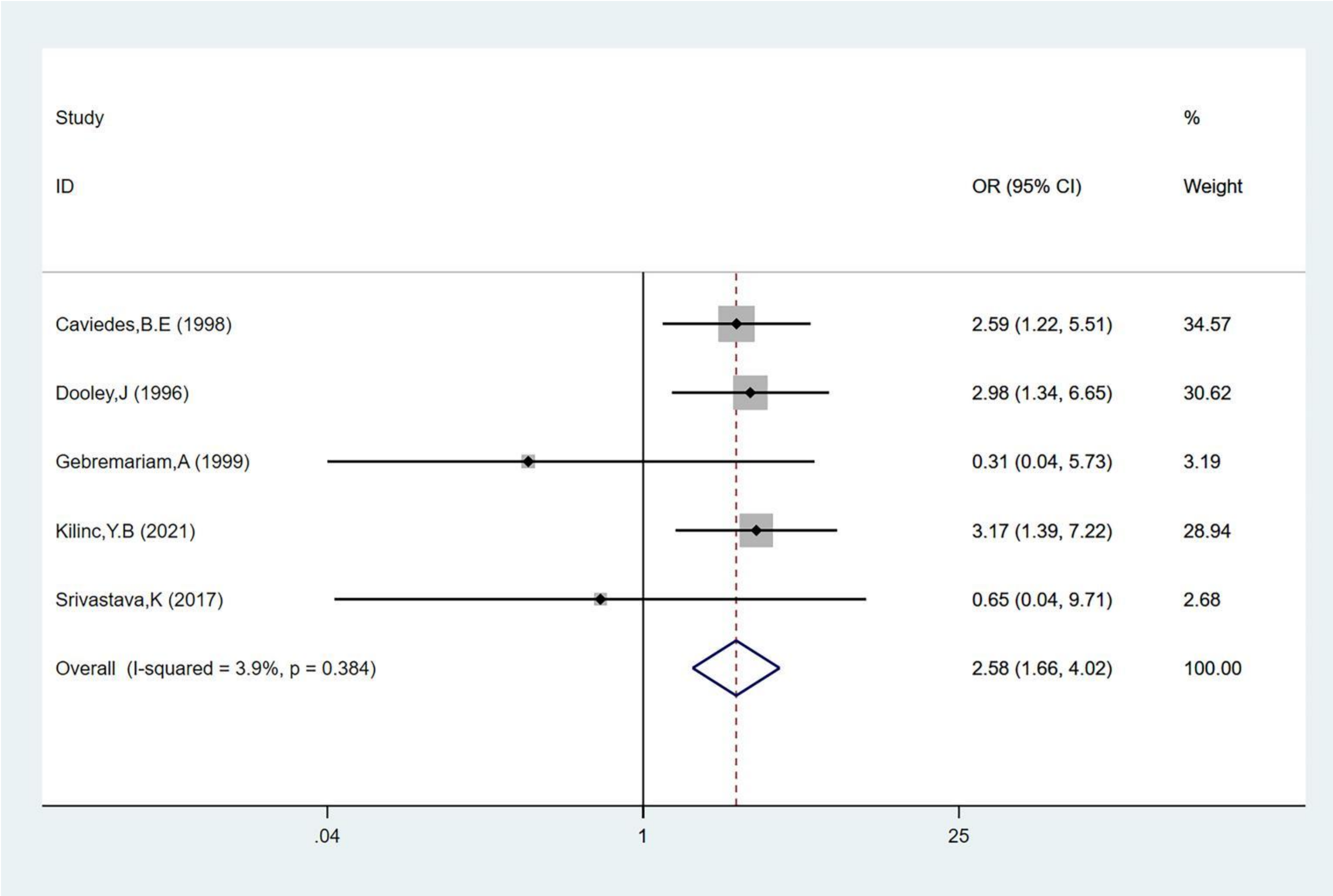

Figure S18 Forest plot of clinical evidence of neurologic abnormalities (Multi-factor)

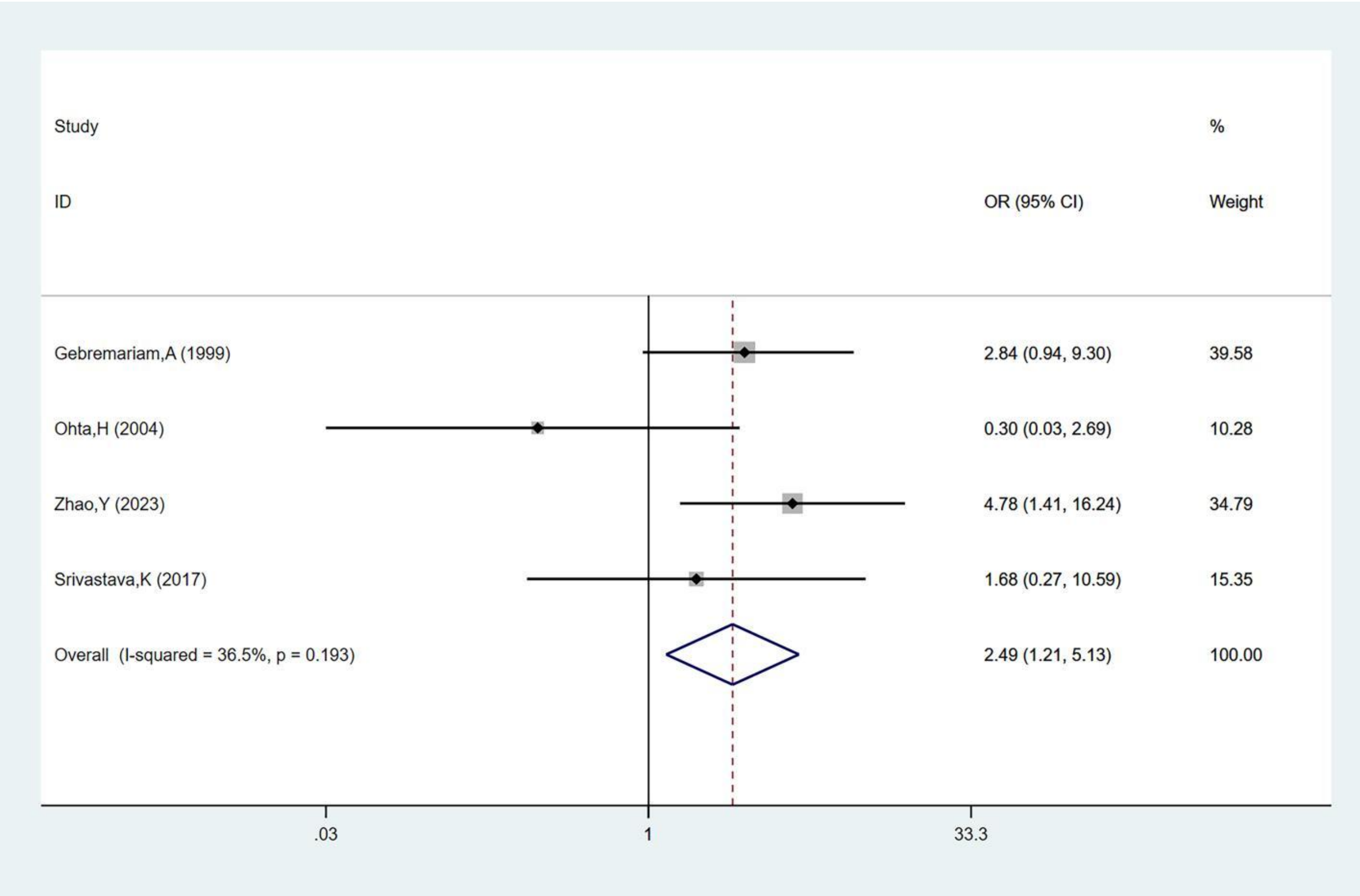

Figure S19 Forest plot of polytherapy (Multi-factor)

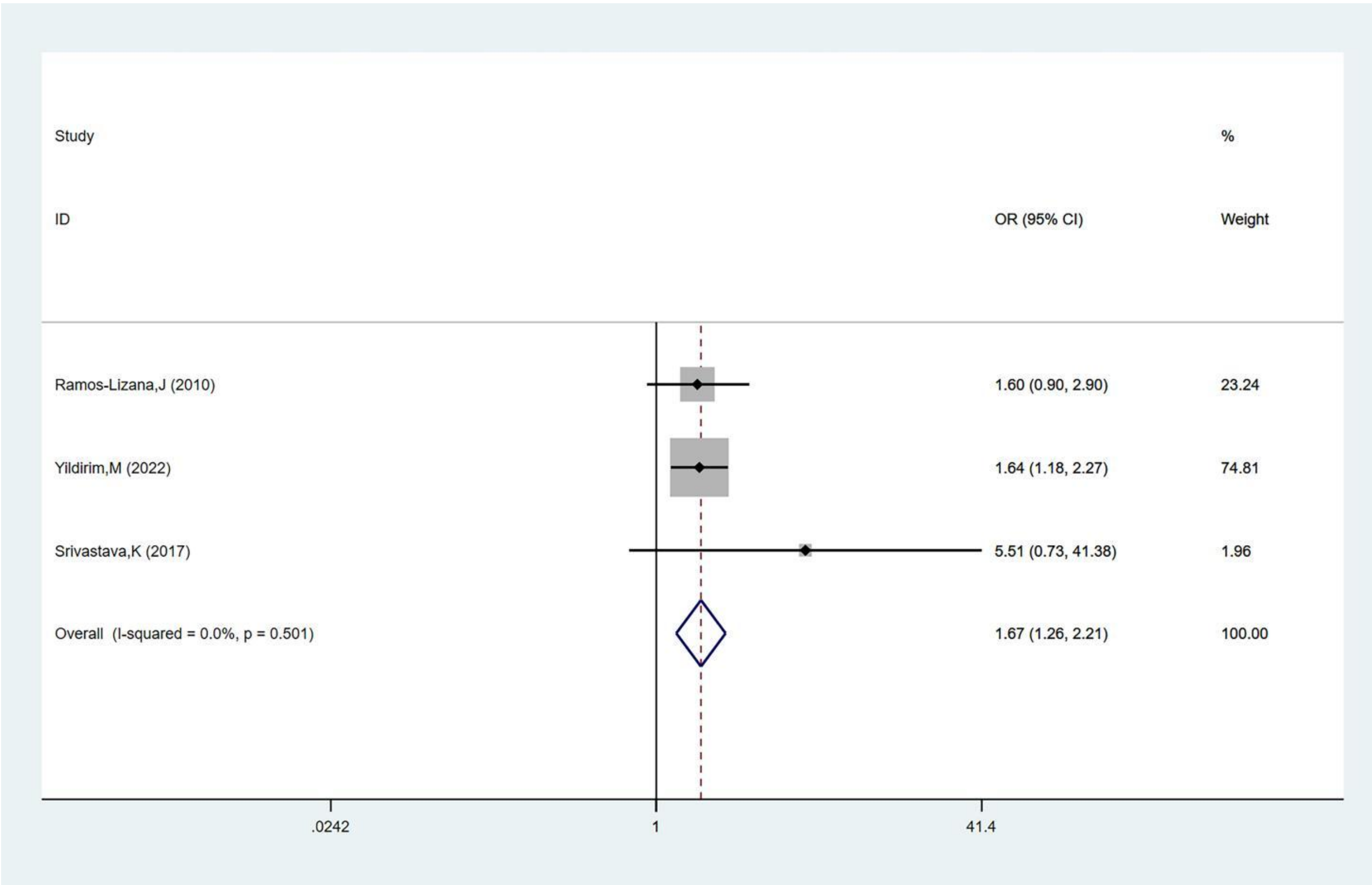

Figure S20 Forest plot of history of seizures during treatment (Multi-factor)

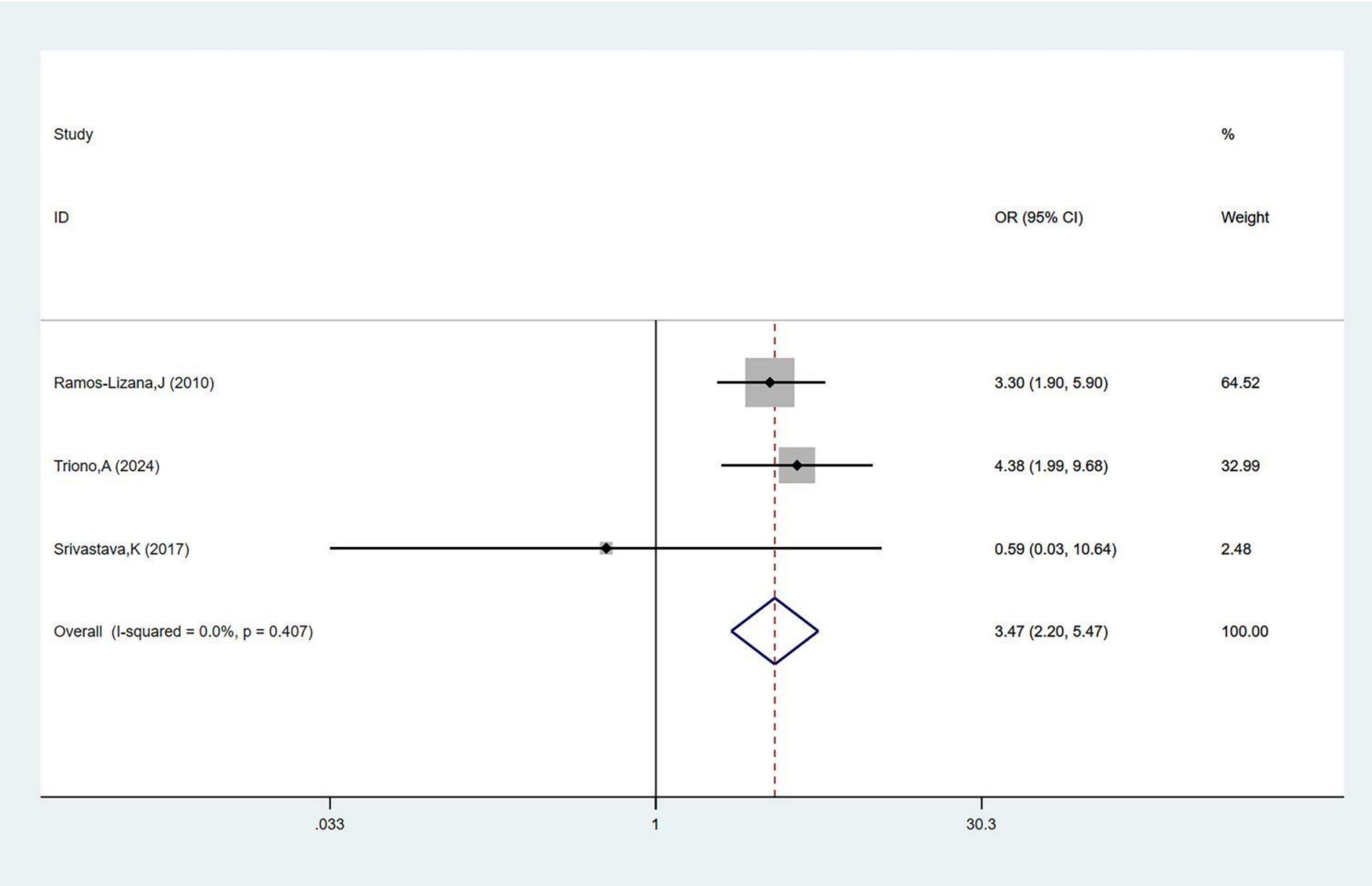

Figure S21 Forest plot of symptomatic etiology (Multi-factor)

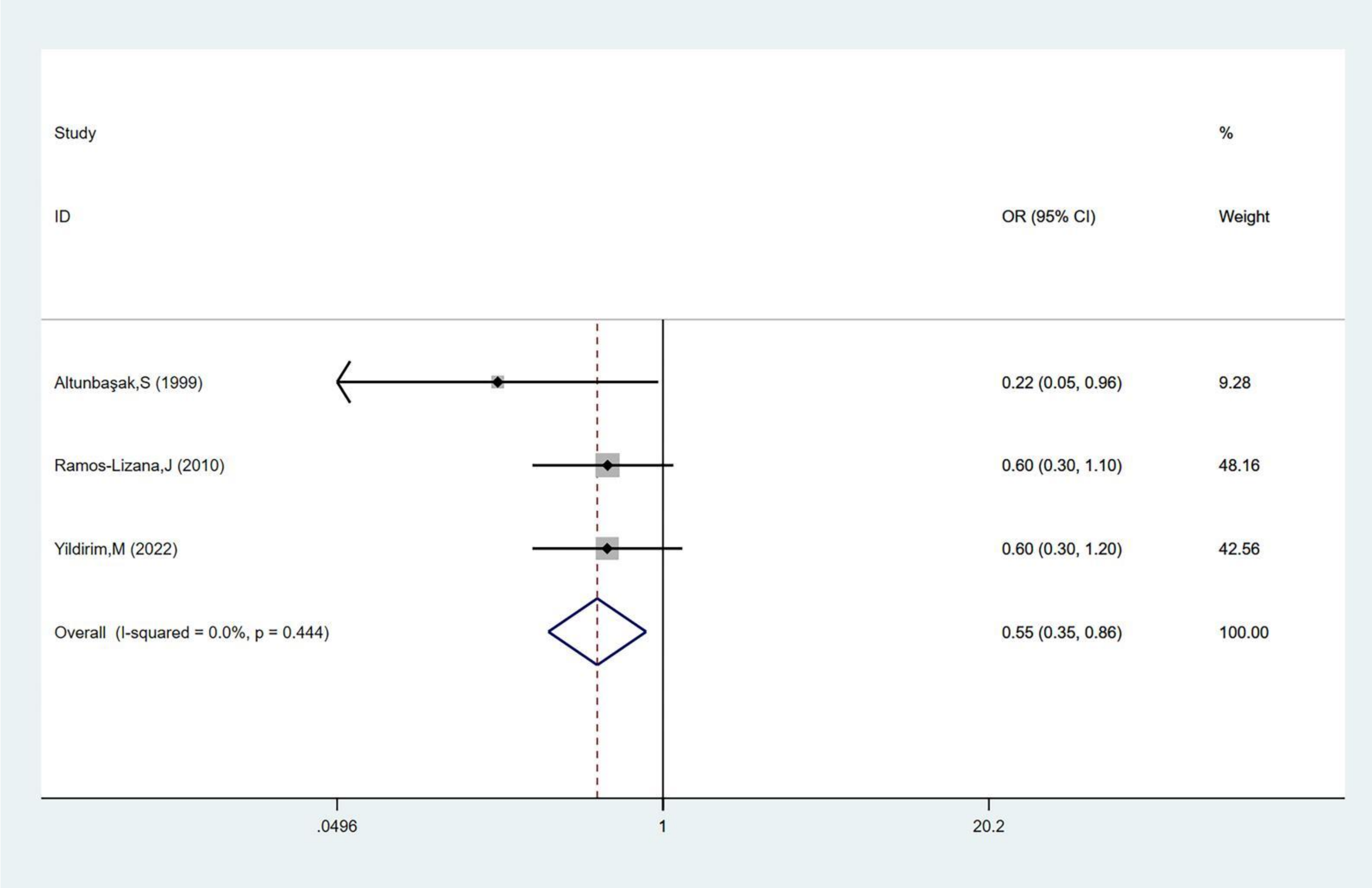

Figure S22 Forest plot of withdrawal period ≥4months (Multi-factor)

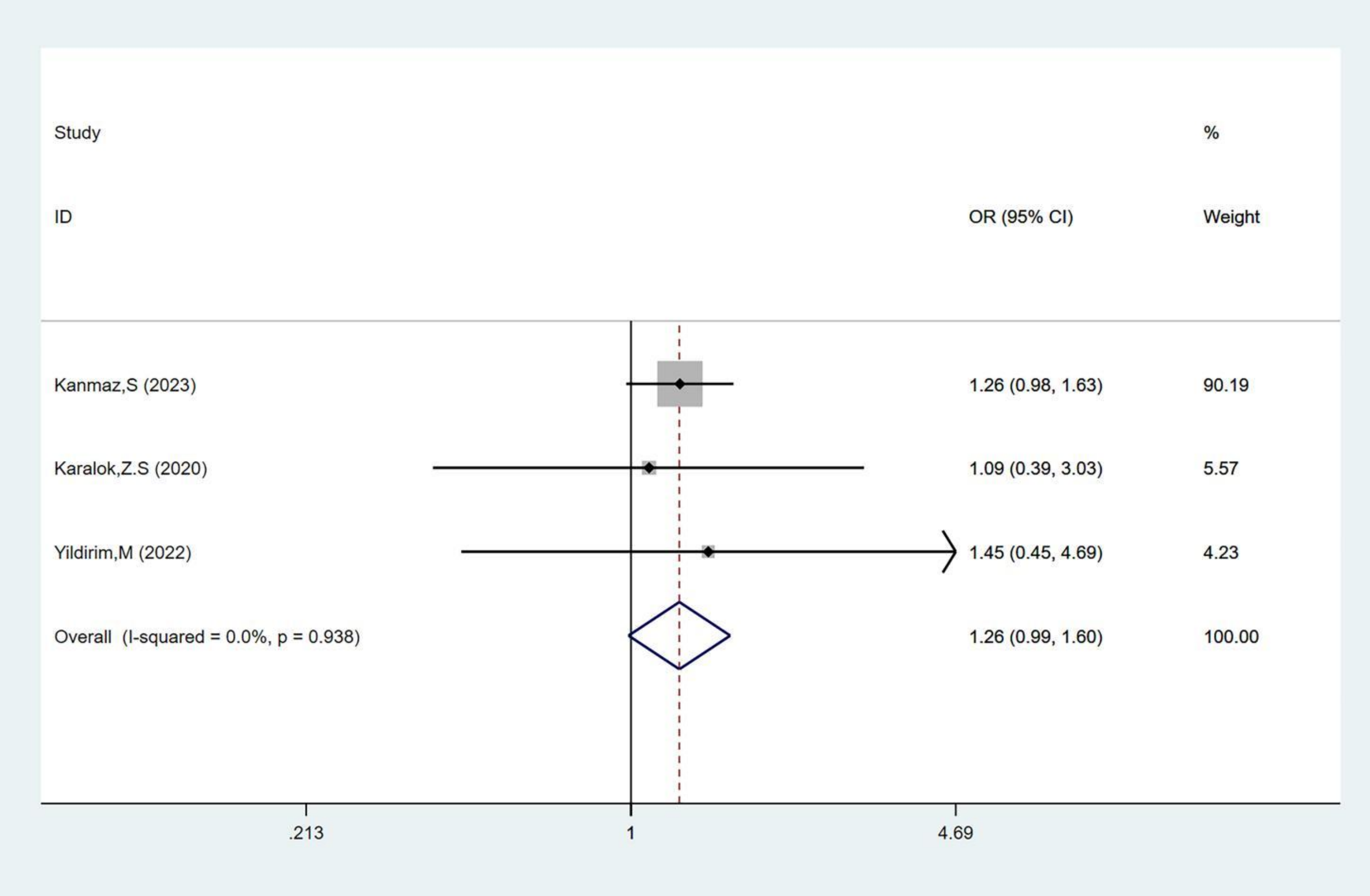

Figure S23 Forest plot of abnormal EEG before withdrawal (Single factor)

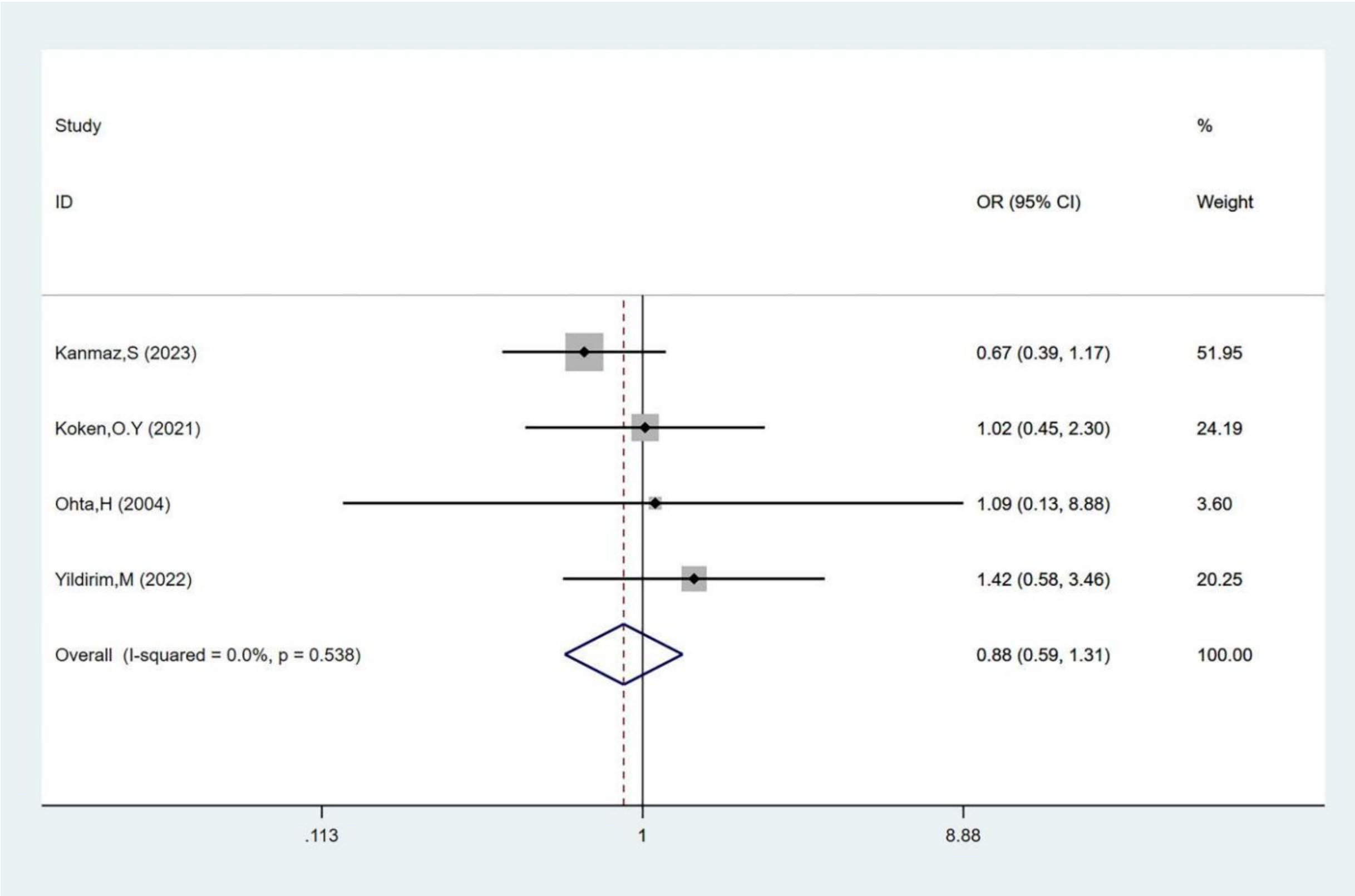

Figure S24 Forest plot of history of status epilepticus (Single factor)

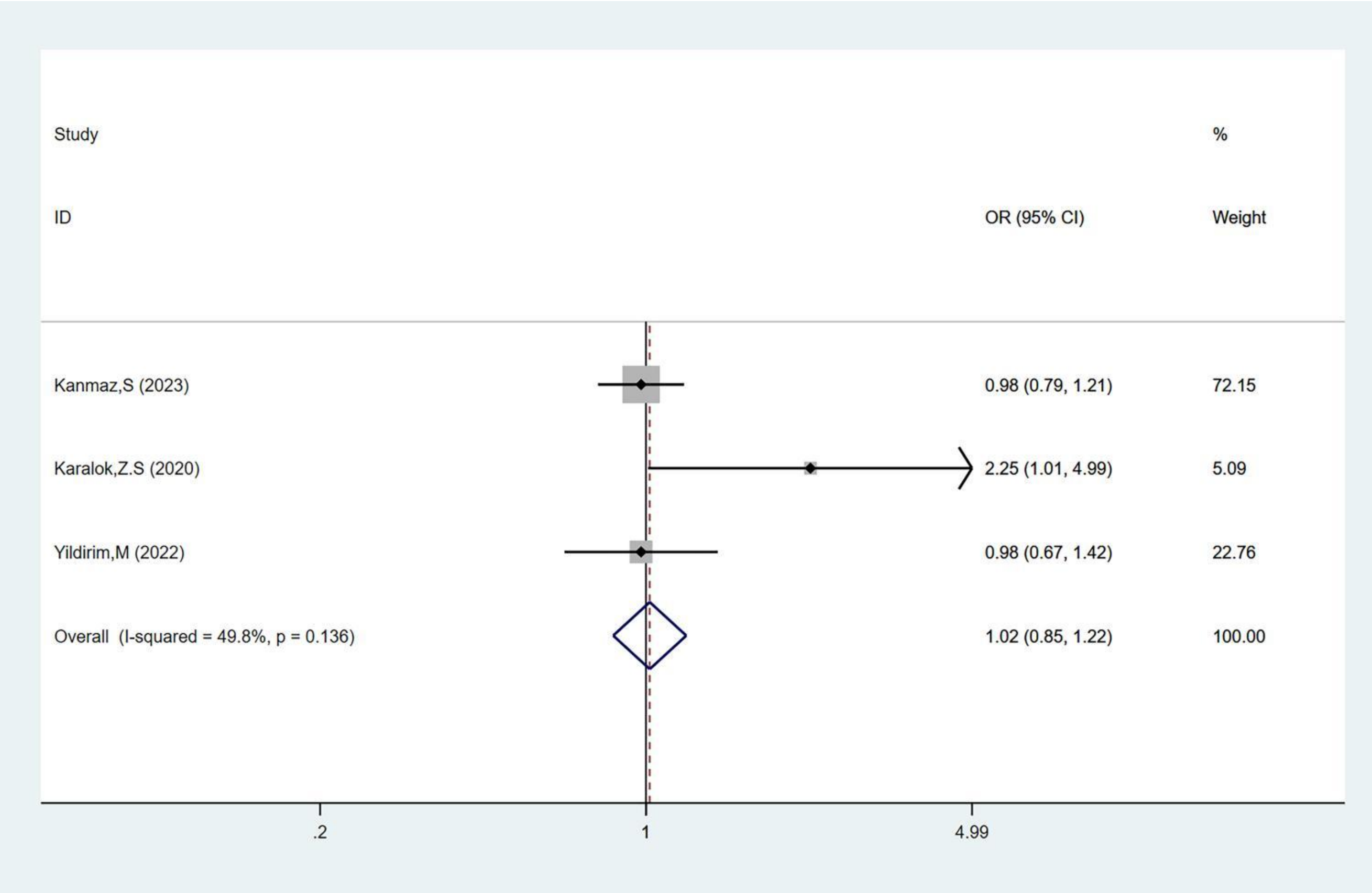

Figure S25 Forest plot of seizure-free period＜3y (Single factor)

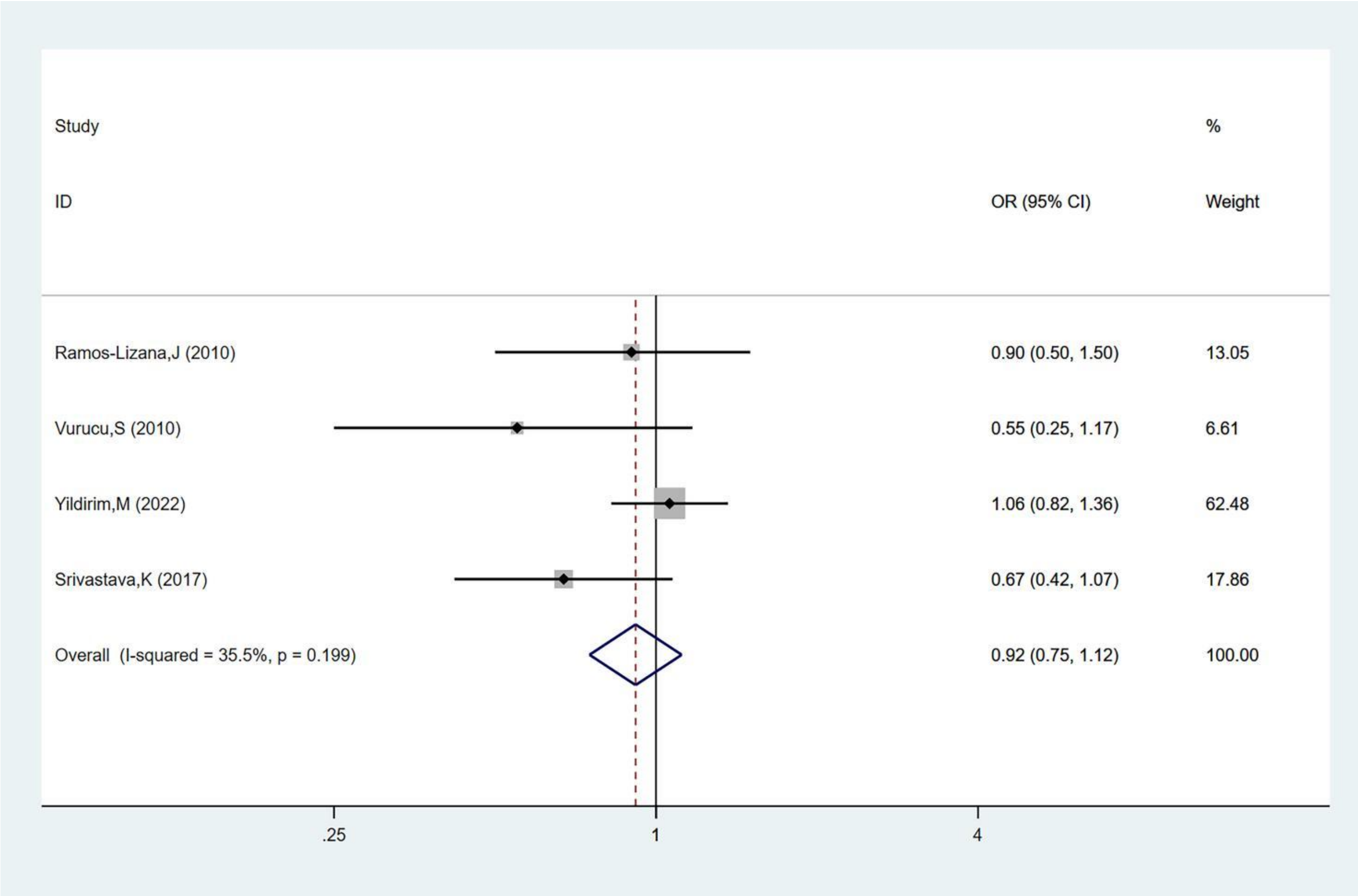

Figure S26 Forest plot of Seizures before treatment≥2 (Single factor)

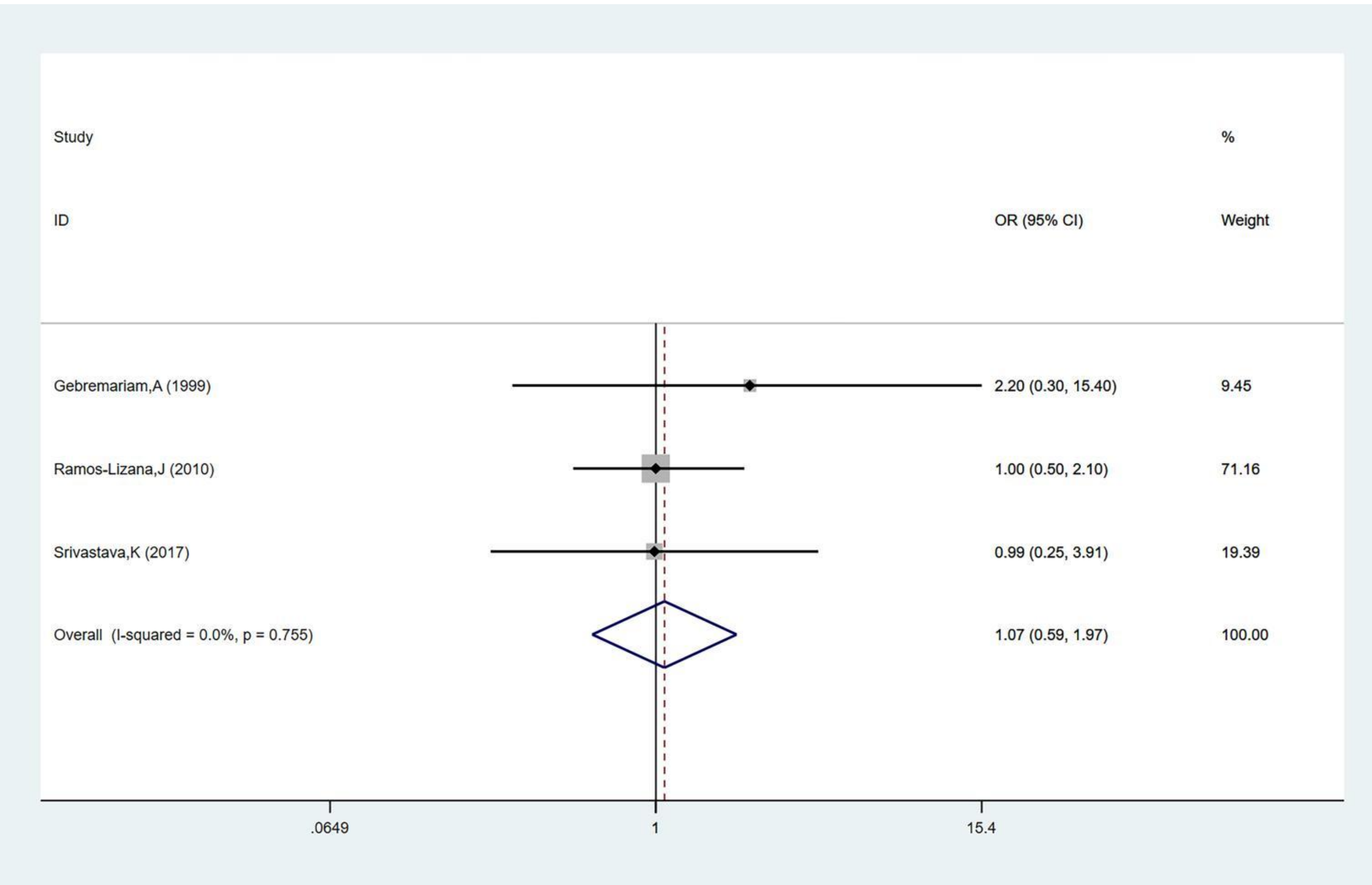

Figure S27 Forest plot of Seizures before treatment≥2 (Multi-factor)

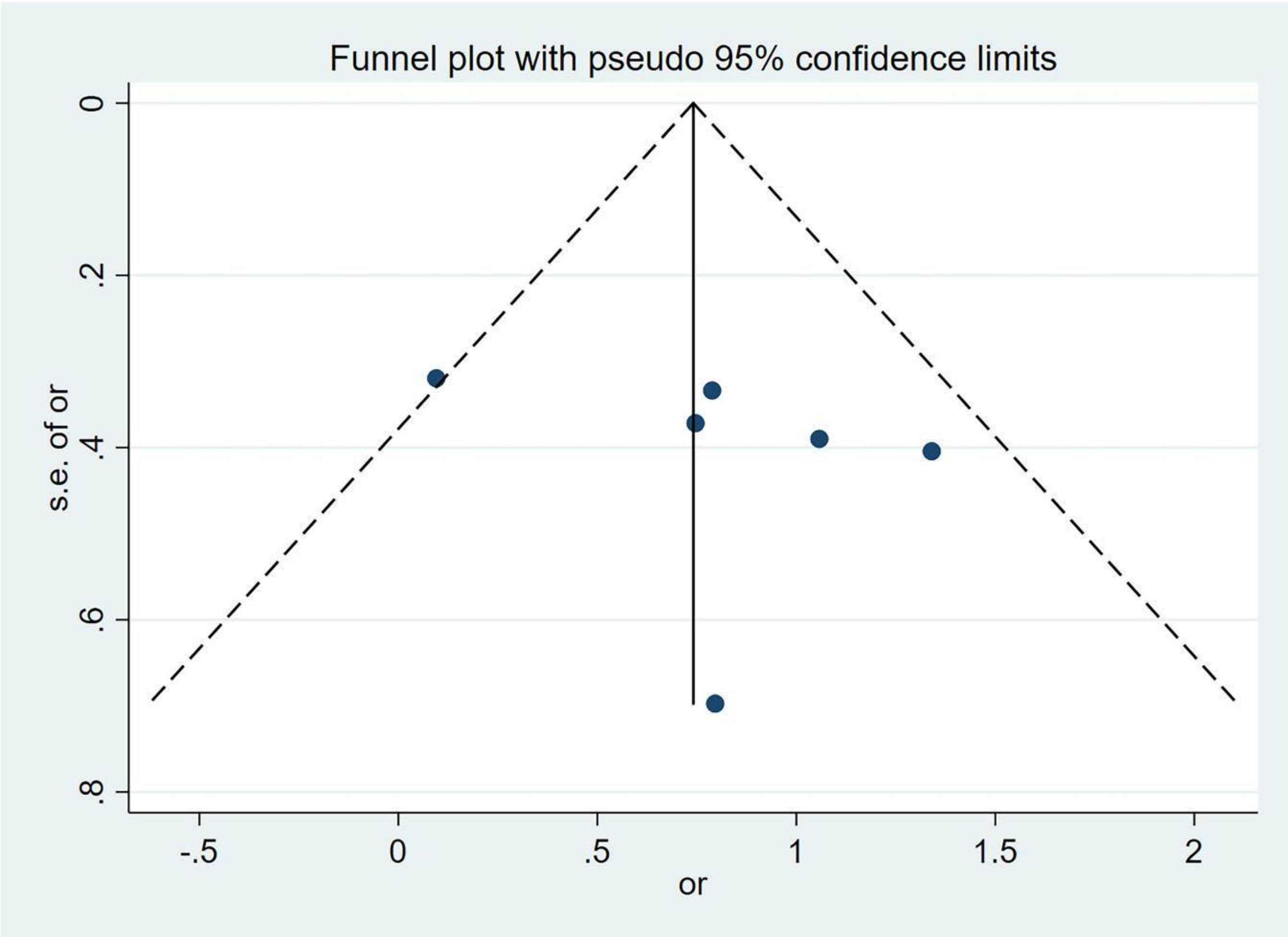

Figure S28 Funnel plot of female gender (Multi-factor)

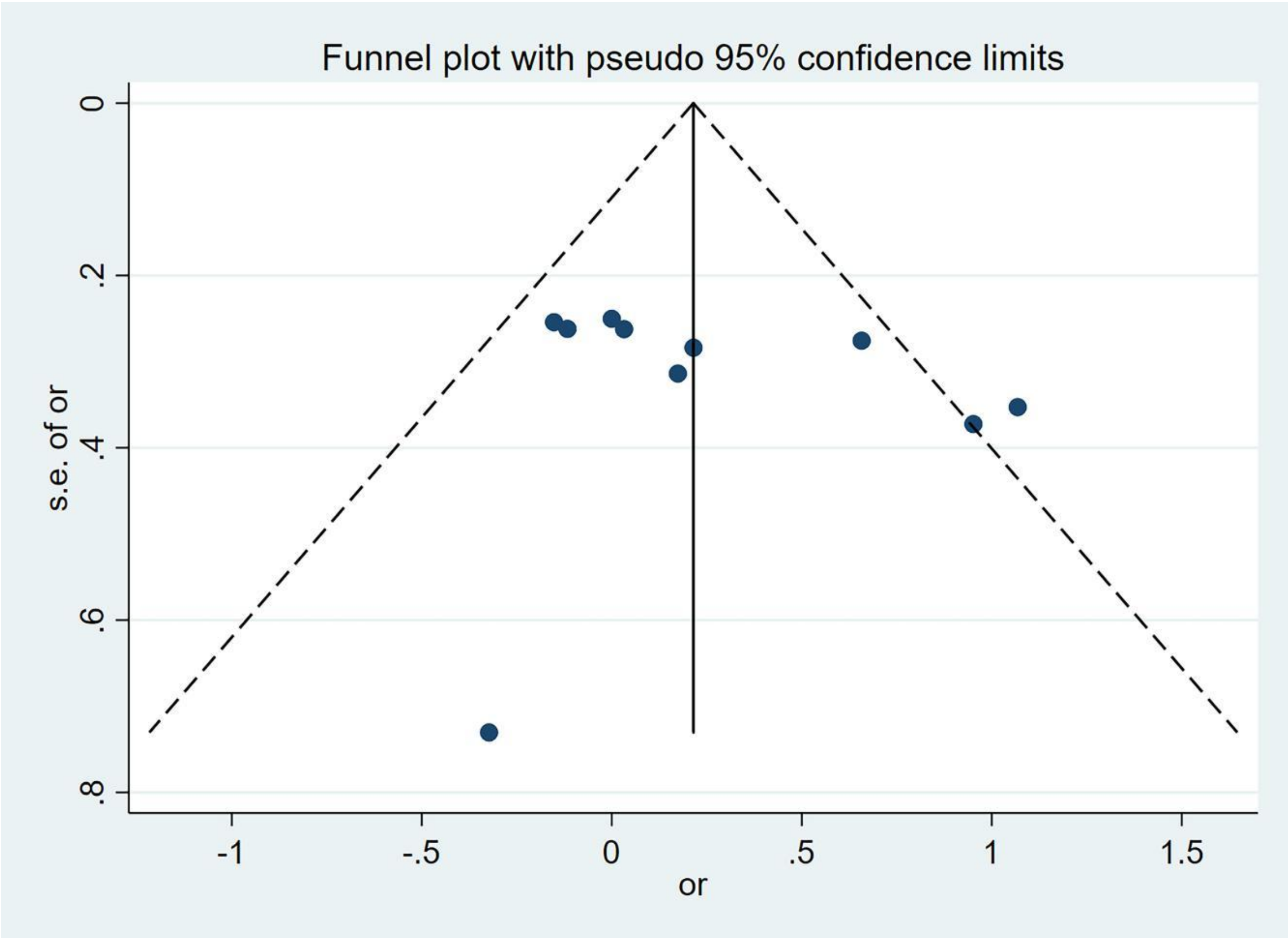

Figure S29 Funnel plot of female gender (Single factor)

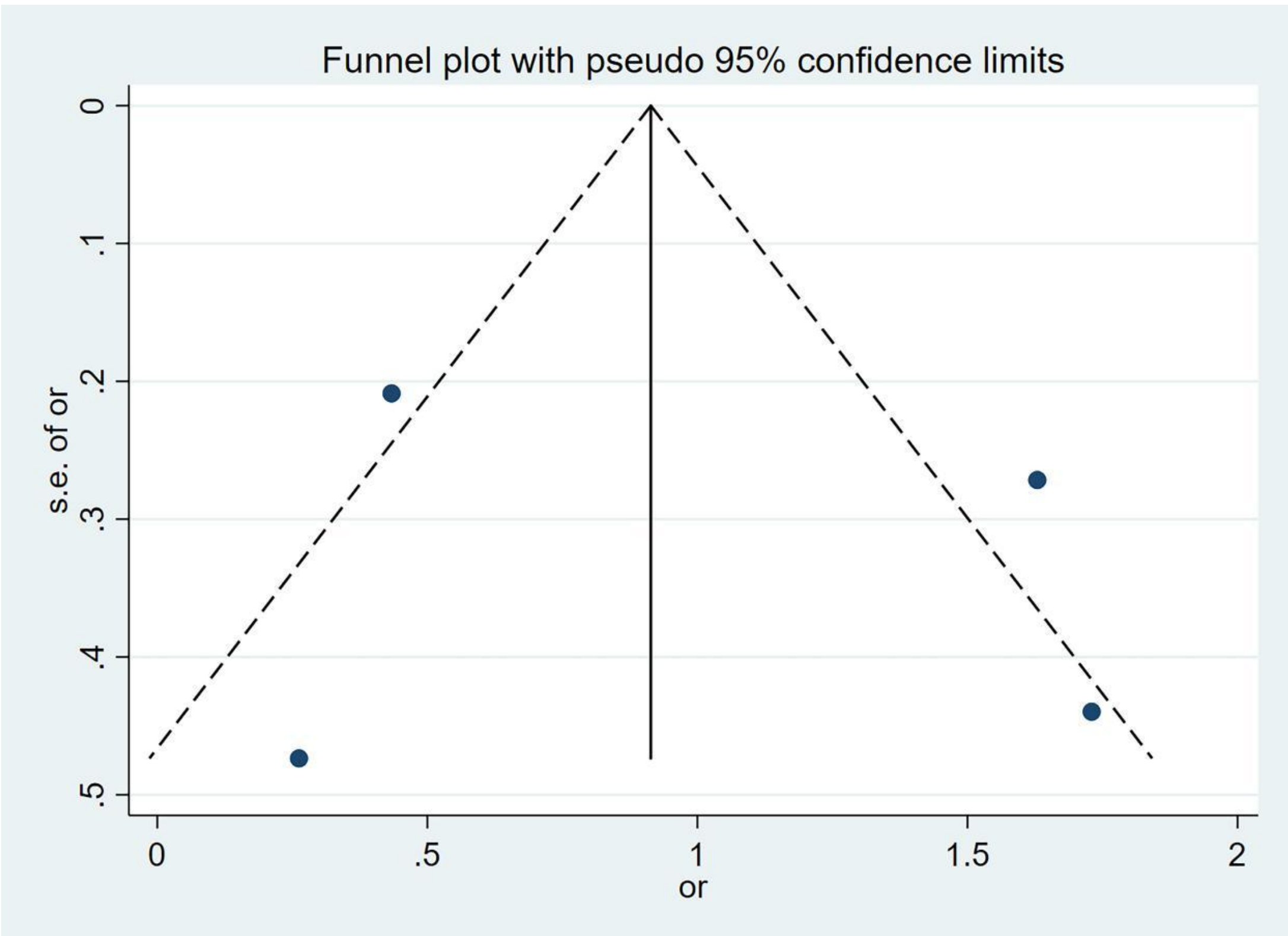

Figure S30 Funnel plot of age of seizure onset≥10y (Multi-factor)

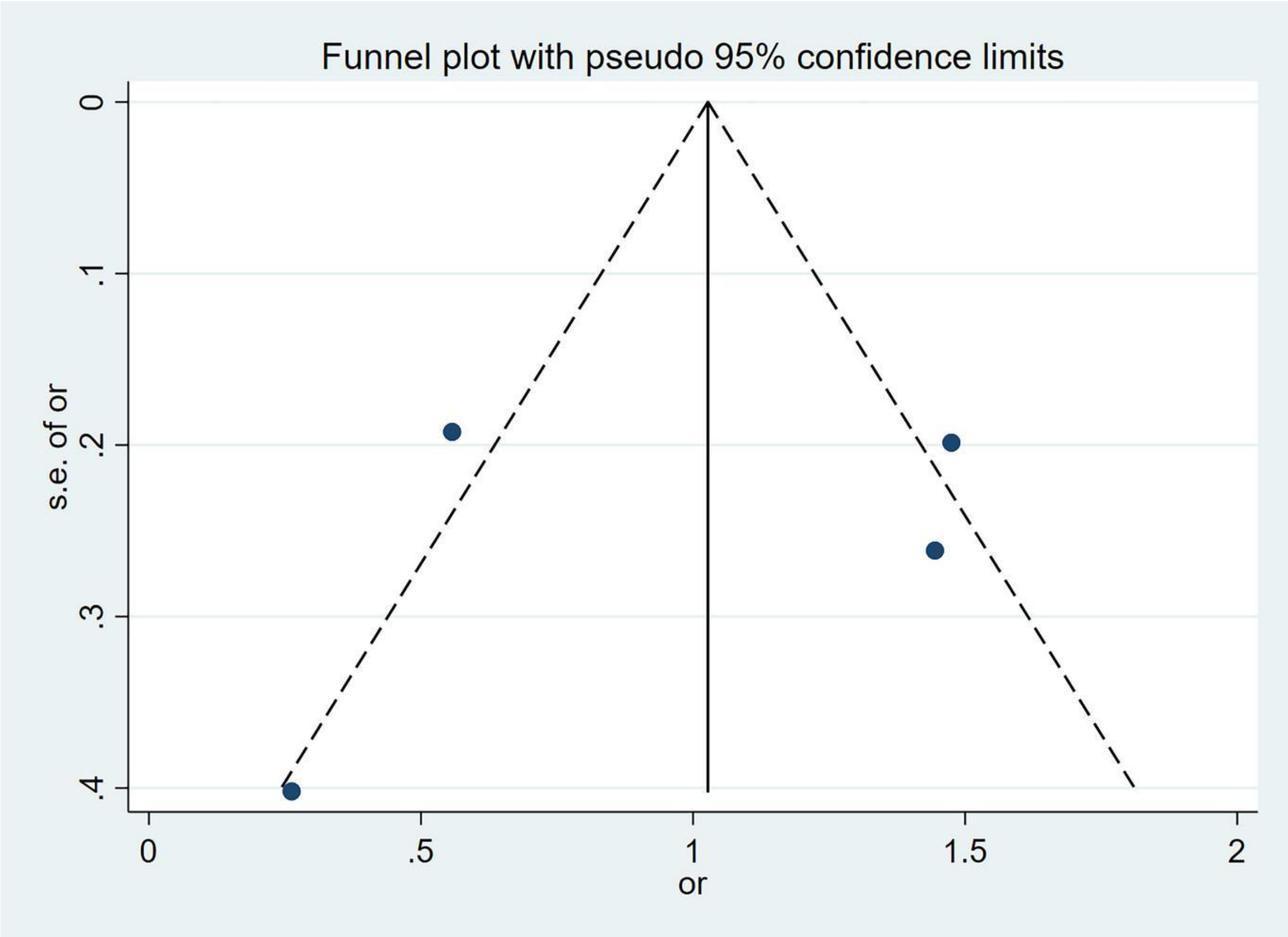

Figure S31 Funnel plot of age of seizure onset≥10y (Single factor)

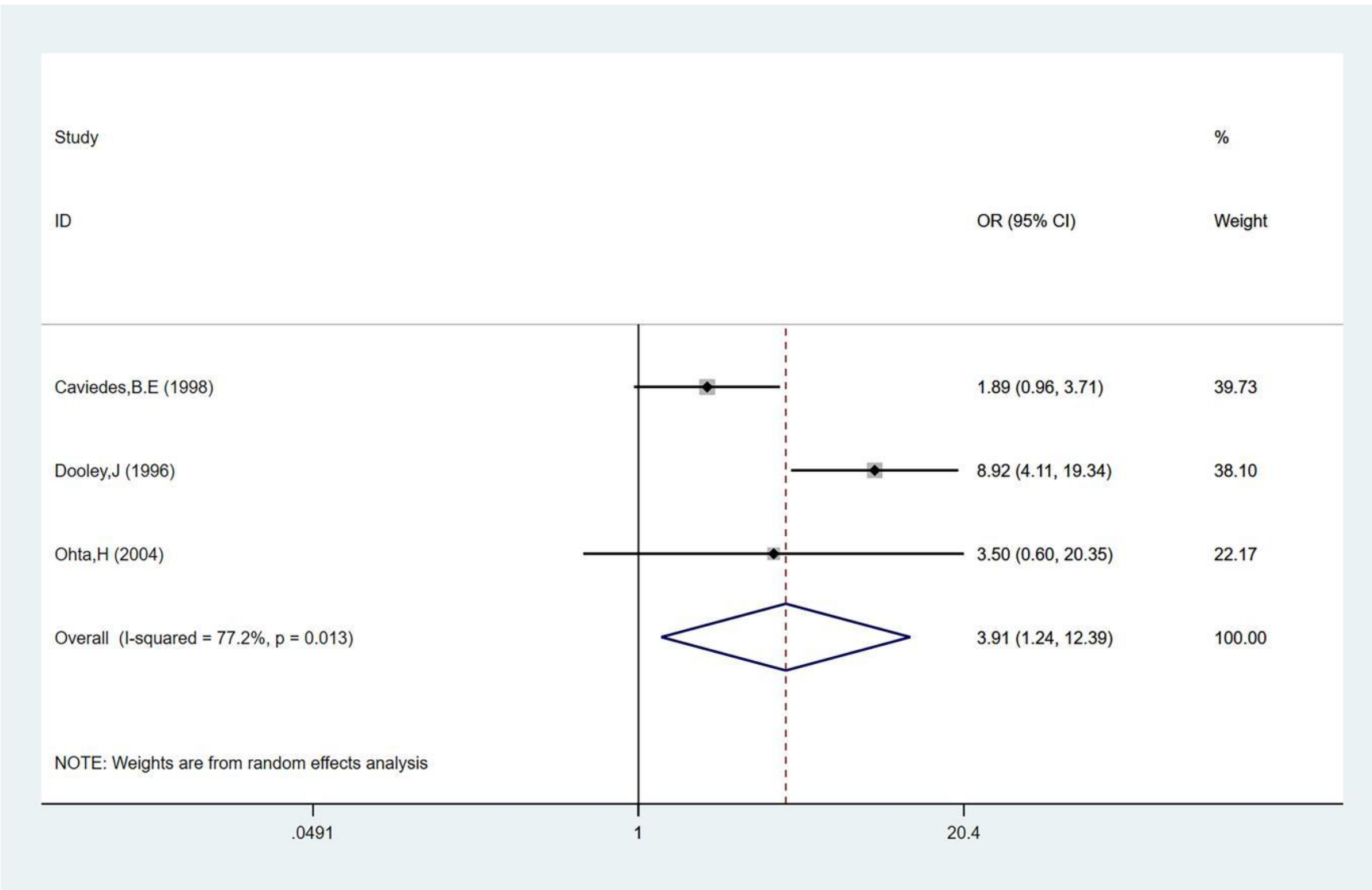

Figure S32 Funnel plot of focal seizure (Multi-factor)

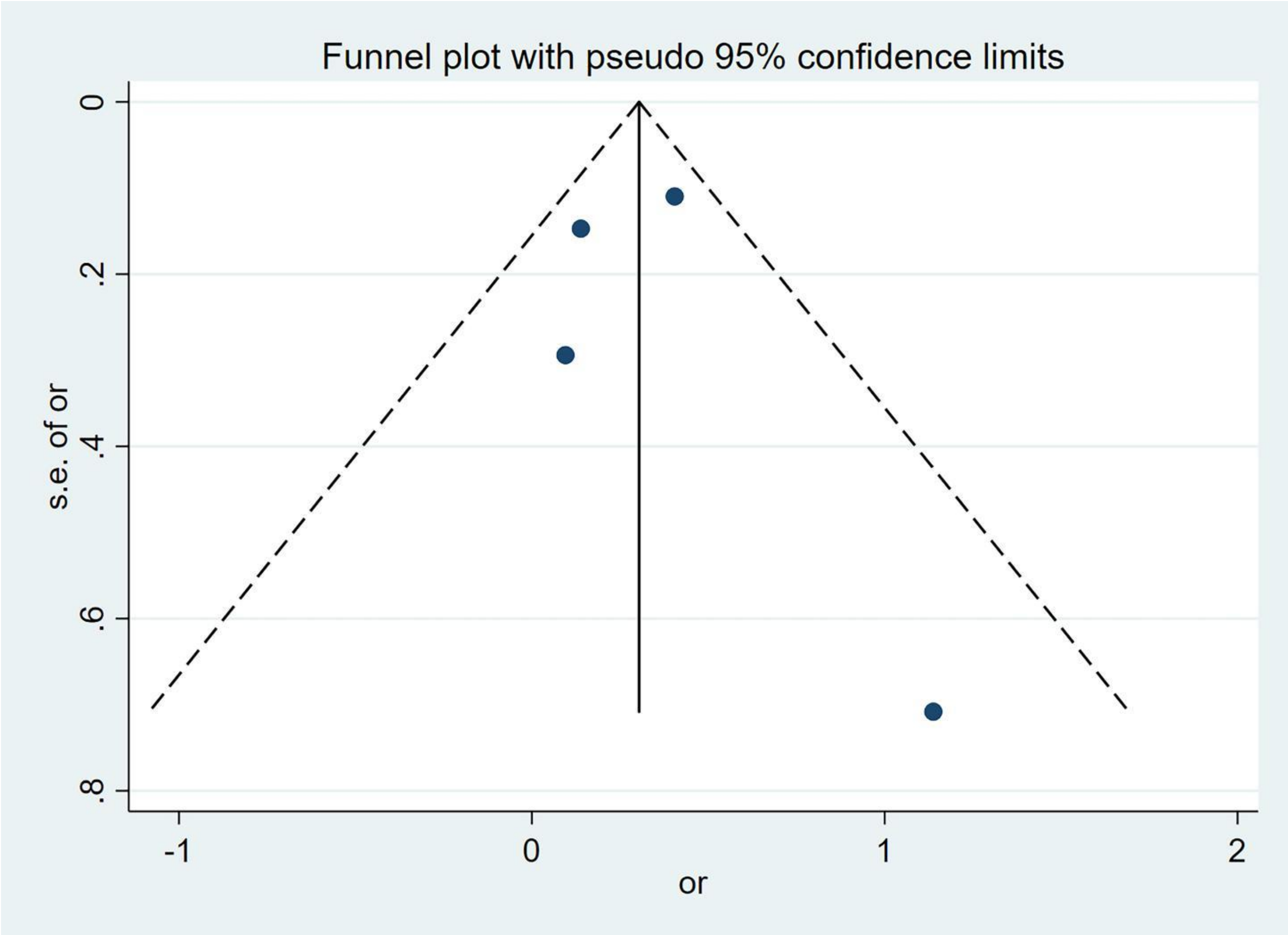

Figure S33 Funnel plot of focal seizure (Single factor)

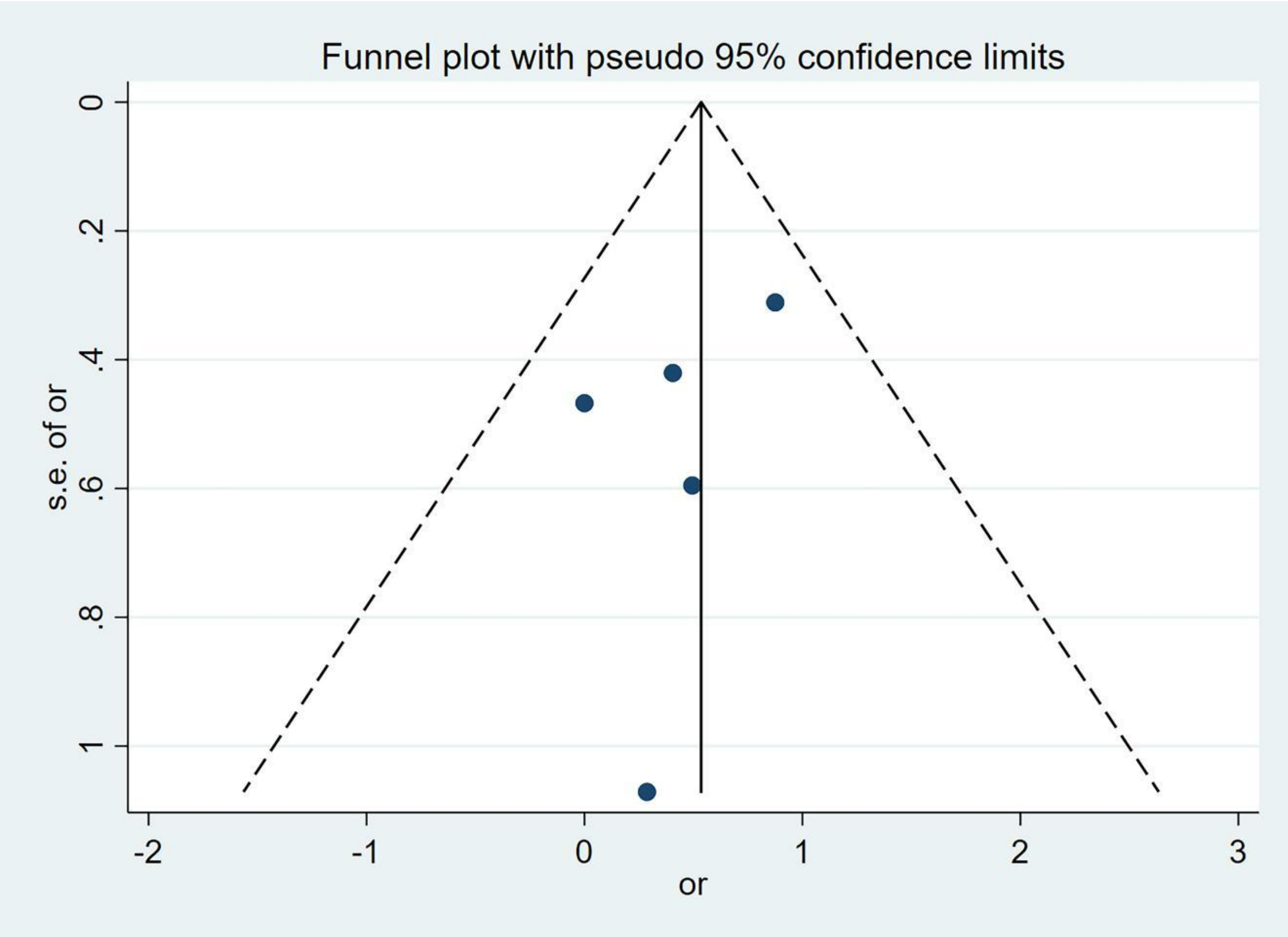

Figure S34 Funnel plot of family history of epilepsy (Multi-factor)

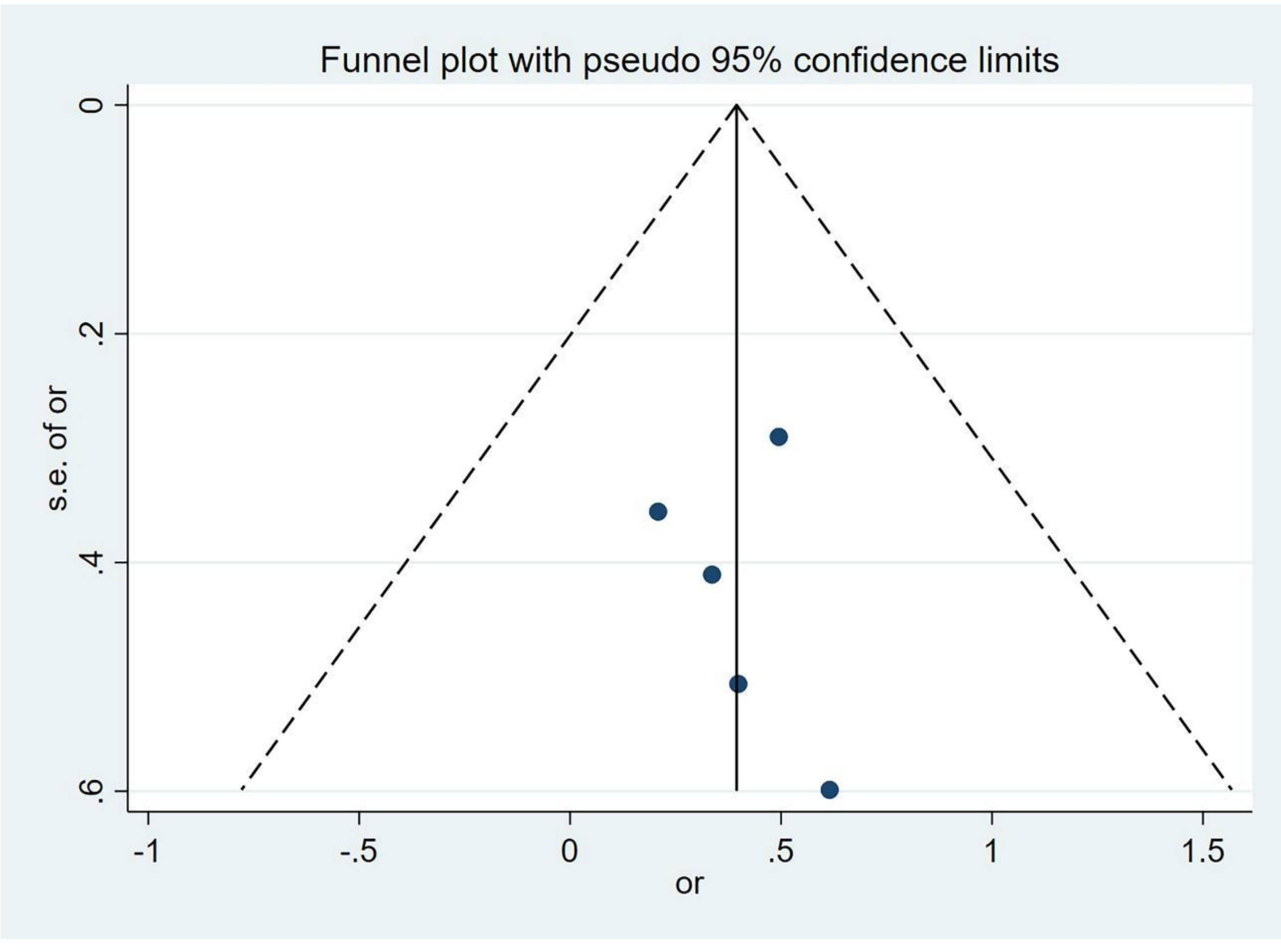

Figure S35 Funnel plot of family history of epilepsy (Single factor)

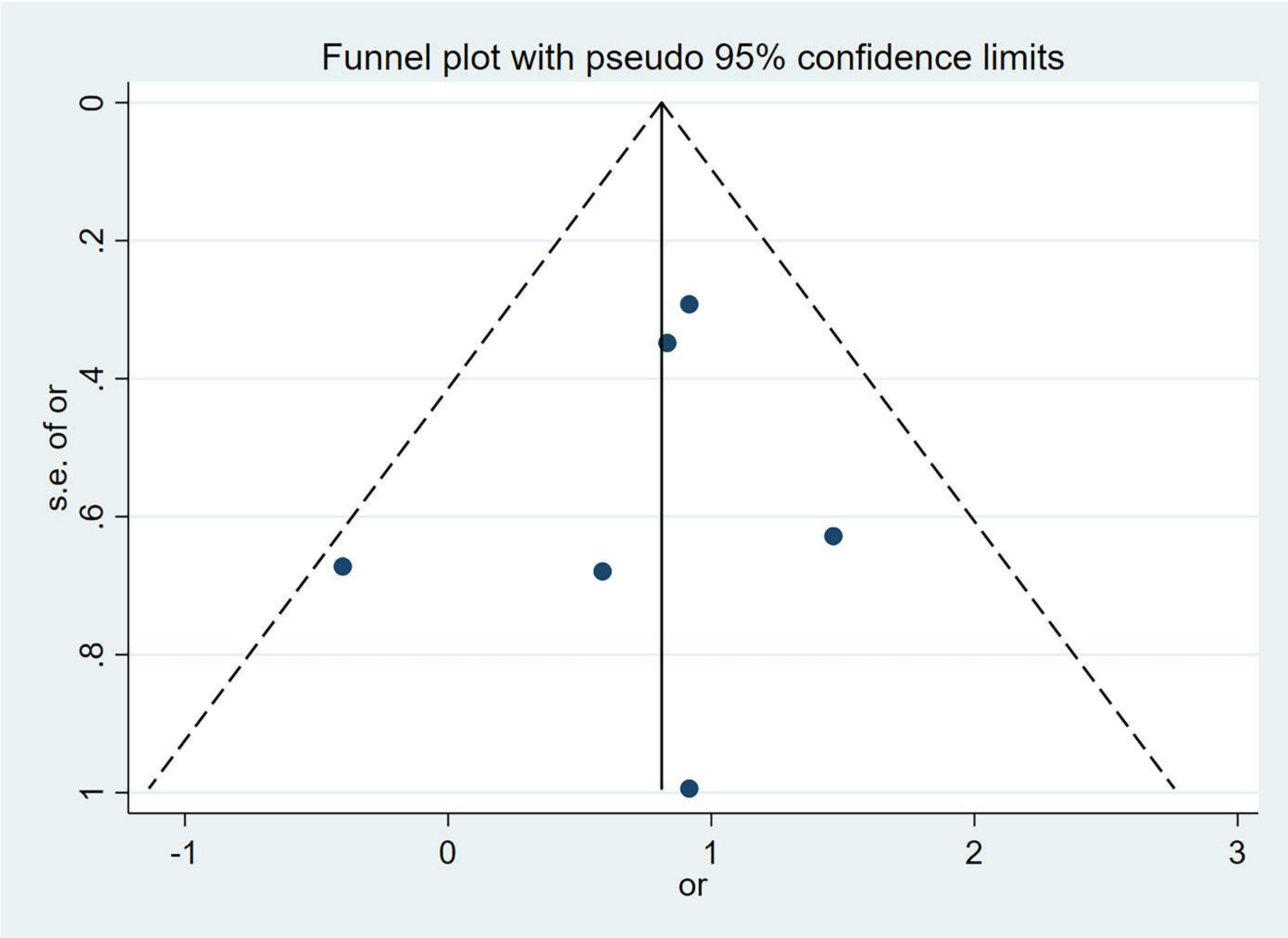

Figure S36 Funnel plot of history of febrile seizure (Multi-factor)

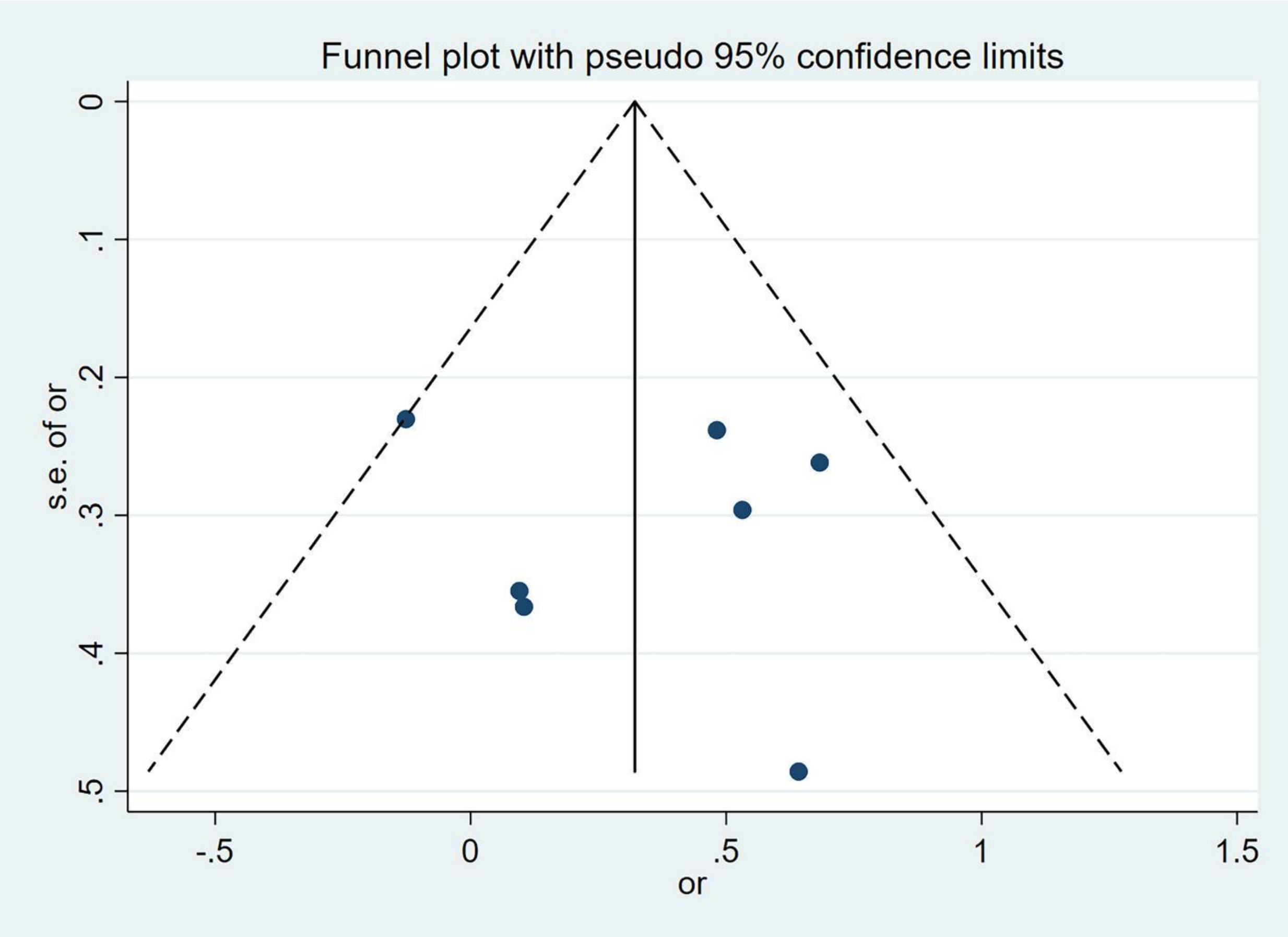

Figure S37 Funnel plot of history of febrile seizure (Single factor)

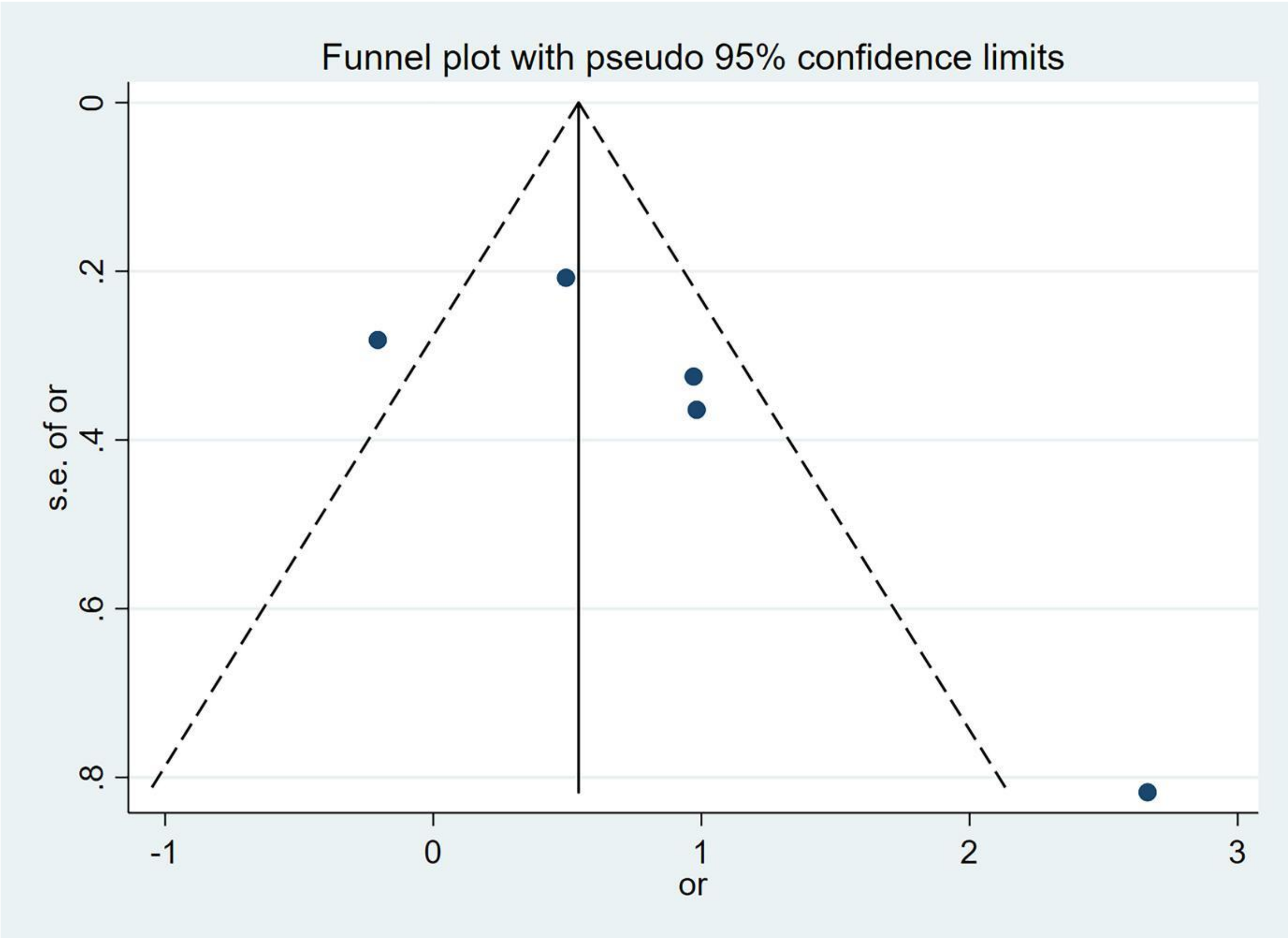

Figure S38 Funnel plot of age at withdrawal ＞10 y (Single factor)

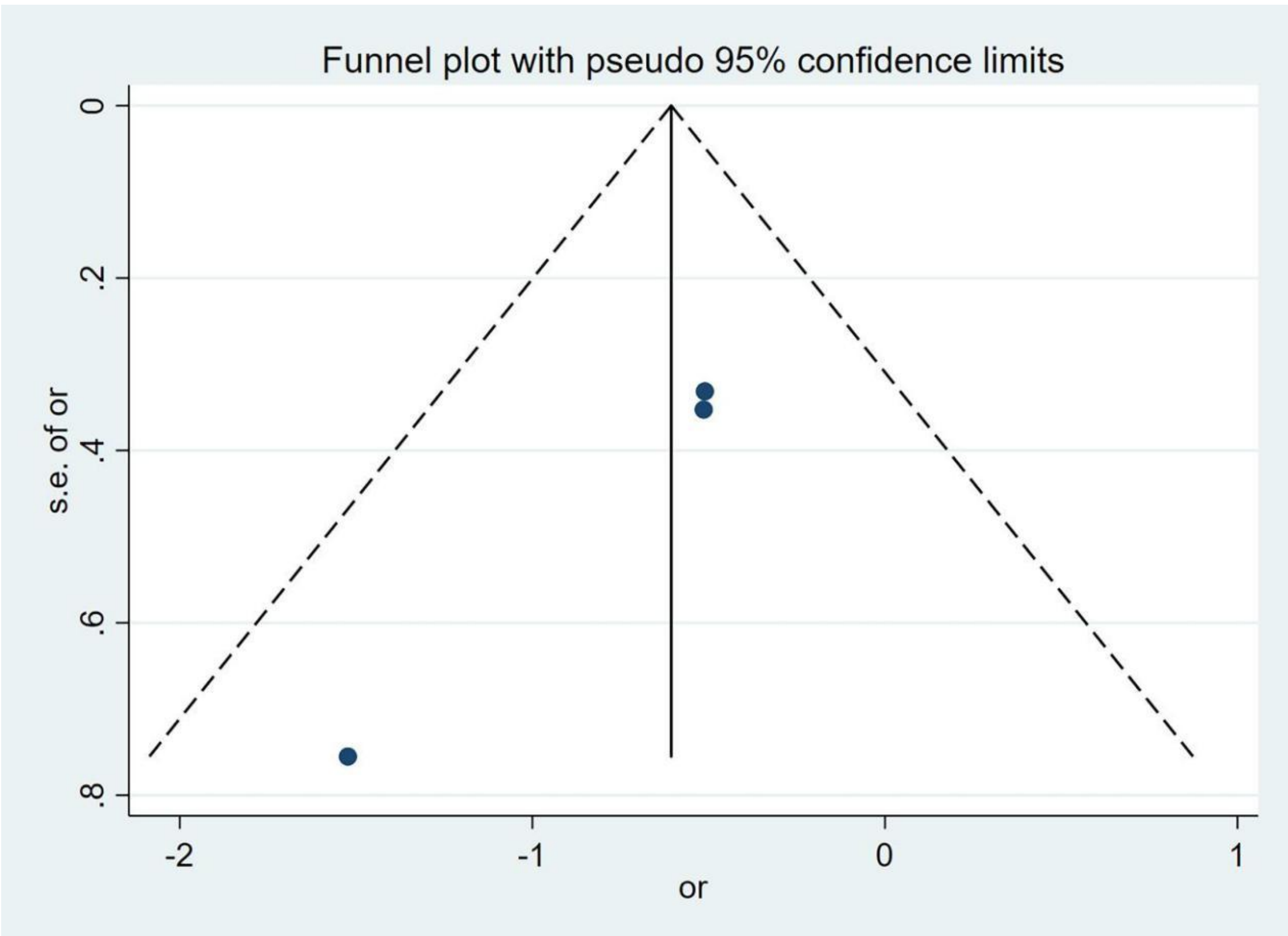

Figure S39 Funnel plot of withdrawal period ≥4months (Multi-factor)

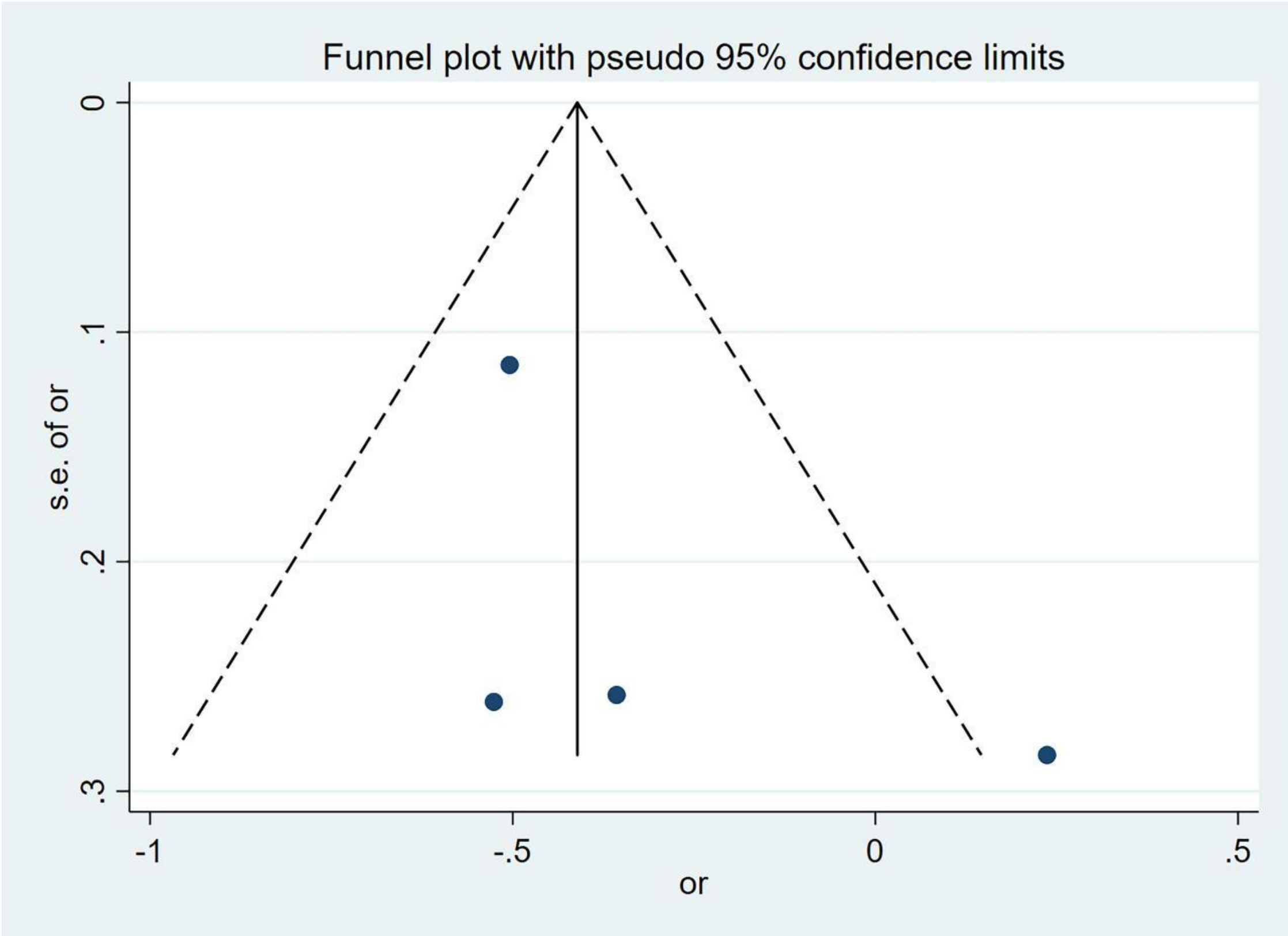

Figure S40 Funnel plot of withdrawal period ≥4months (Single factor)

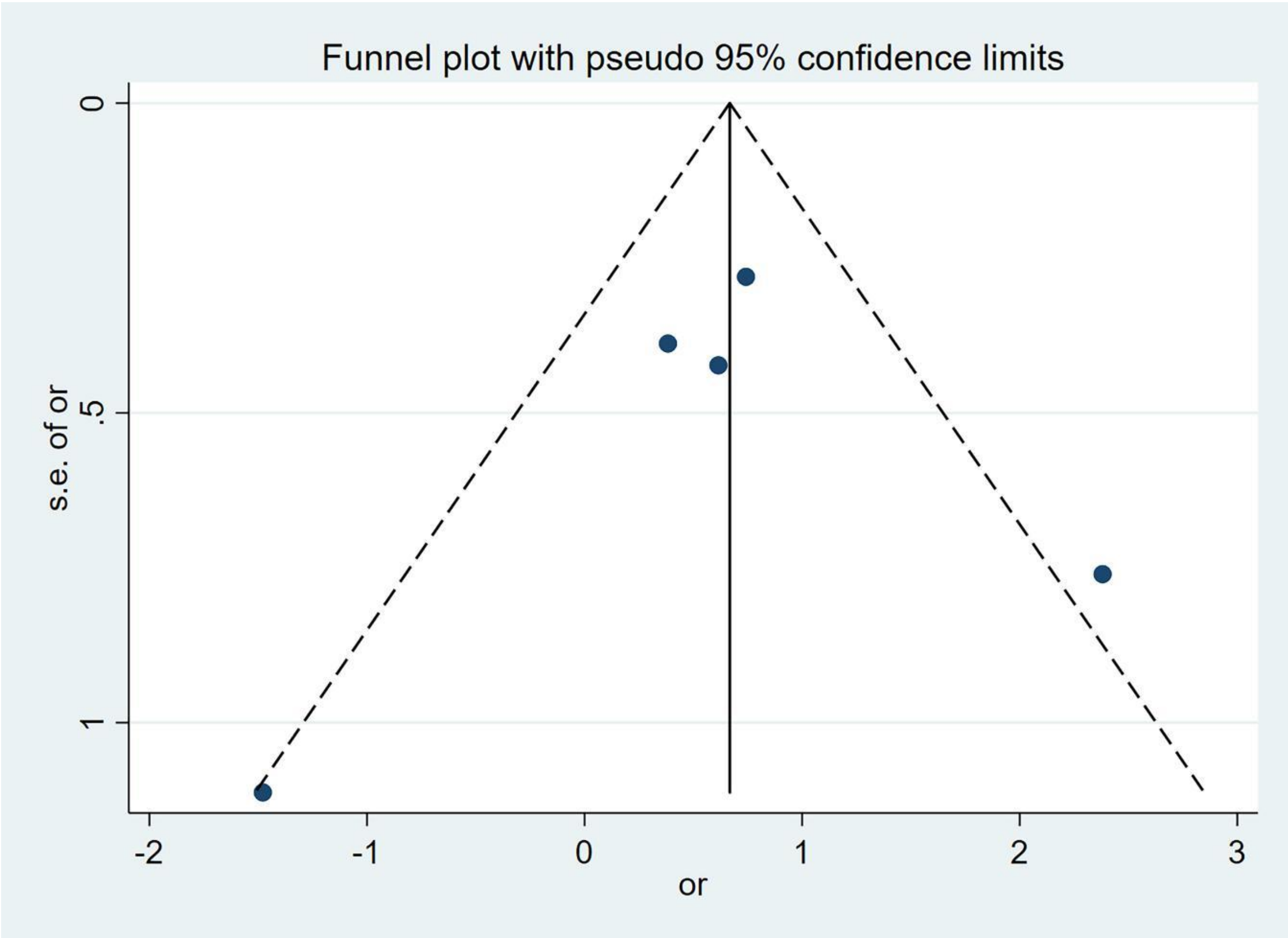

Figure S41 Funnel plot of delayed development or intellectual disability (Multi-factor)

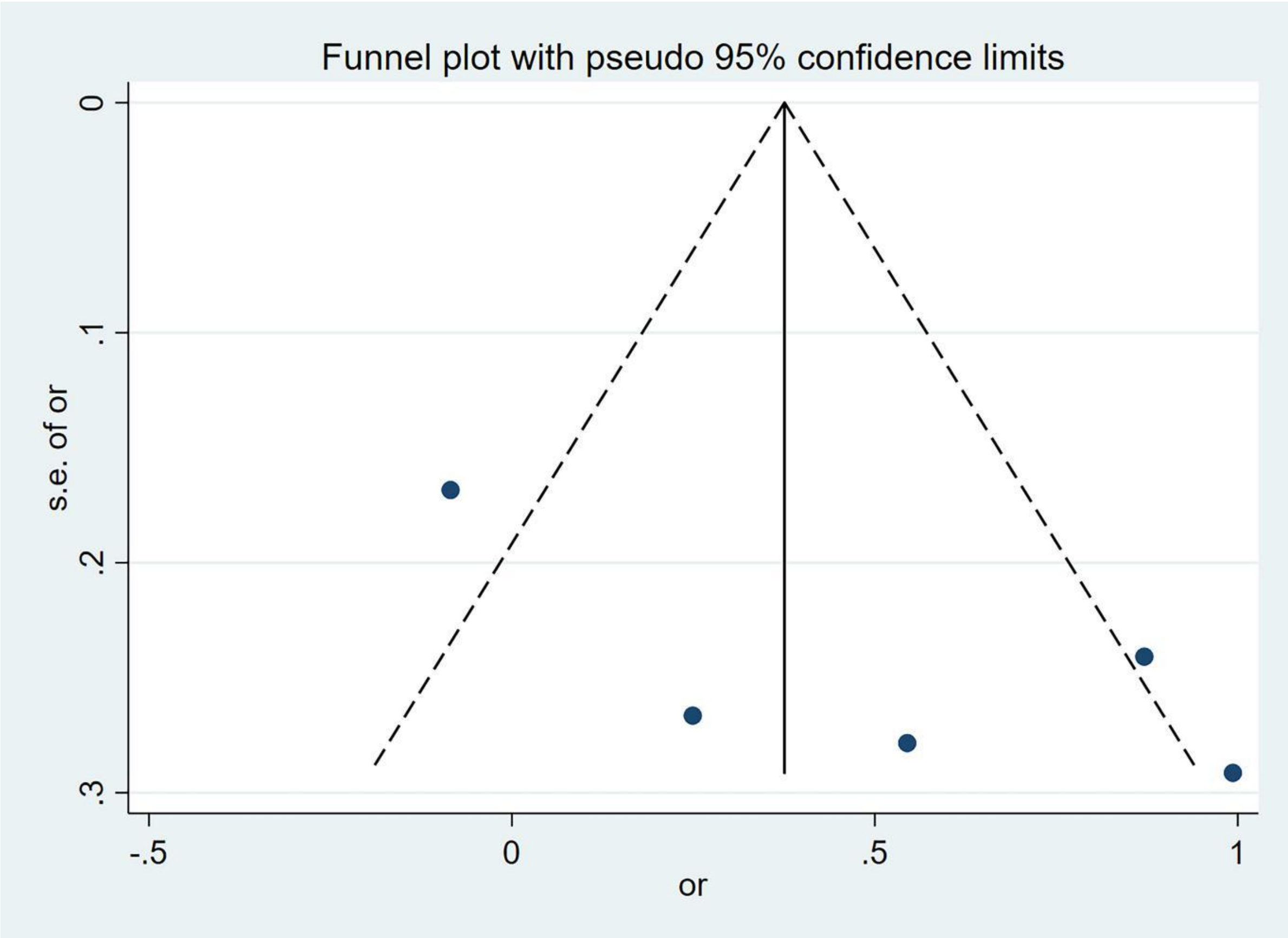

Figure S42 Funnel plot of delayed development or intellectual disability (Single factor)

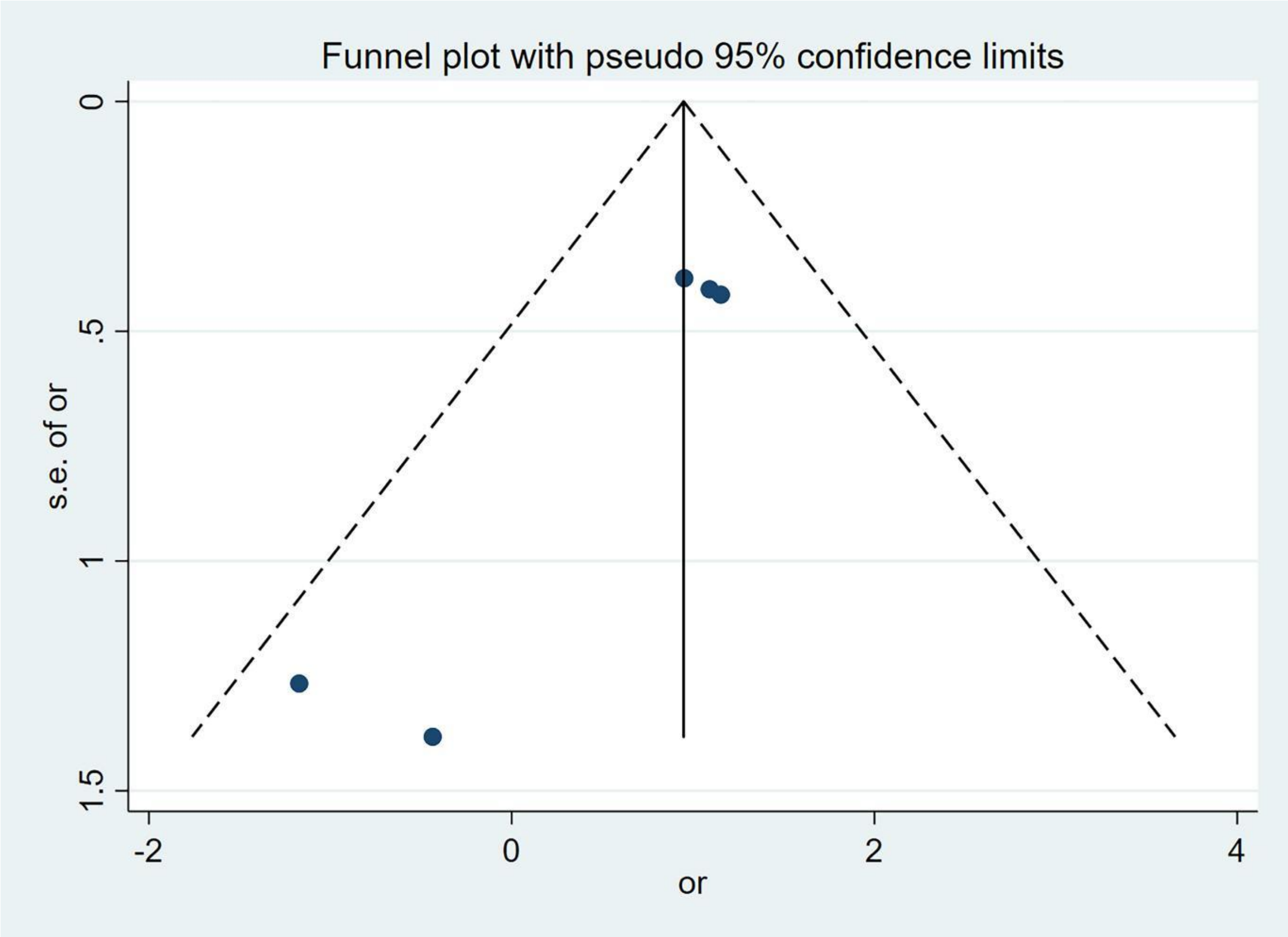

Figure S43 Funnel plot of clinical evidence of neurologic abnormalities (Multi-factor)

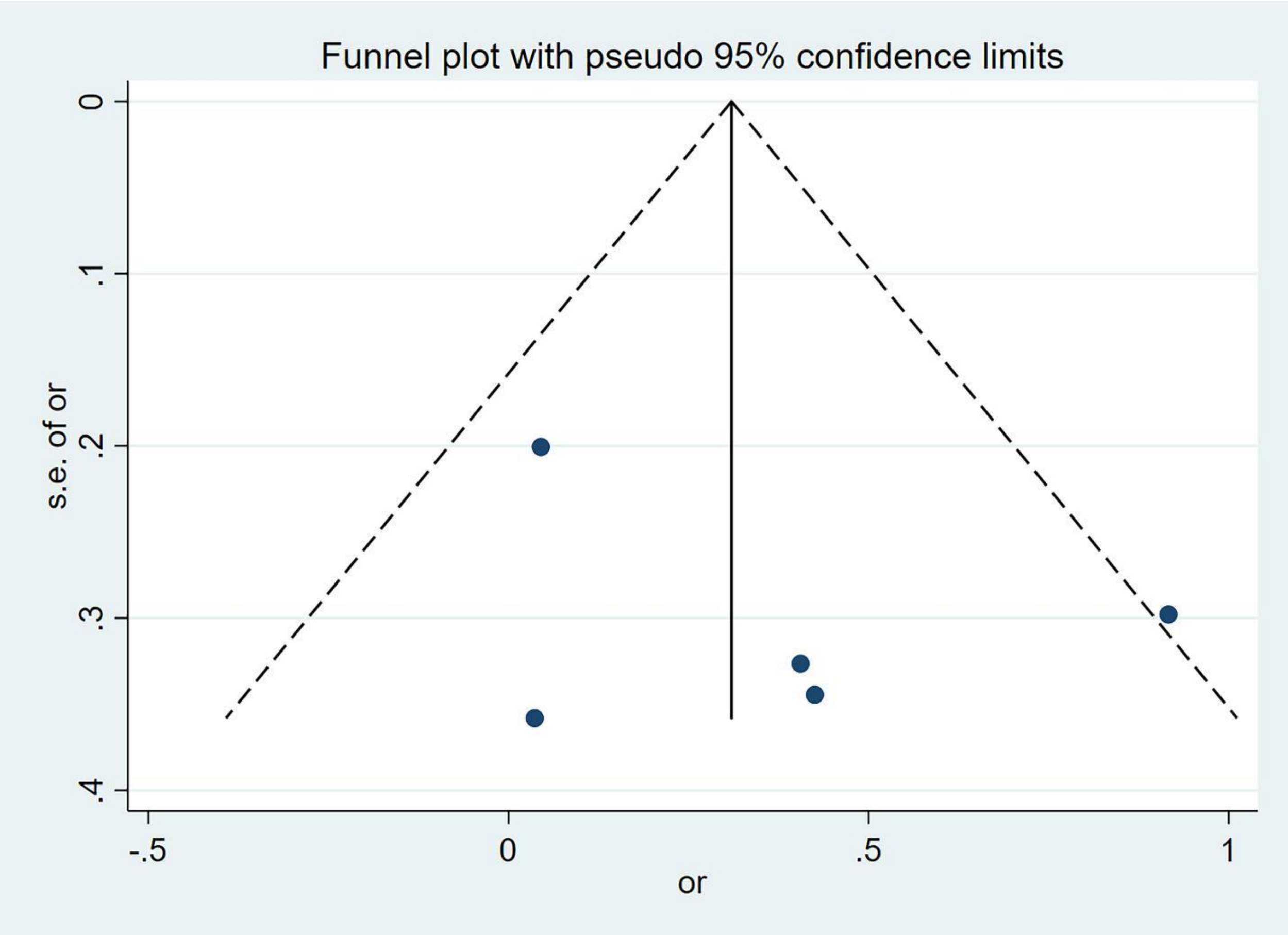

Figure S44 Funnel plot of clinical evidence of neurologic abnormalities (Single factor)

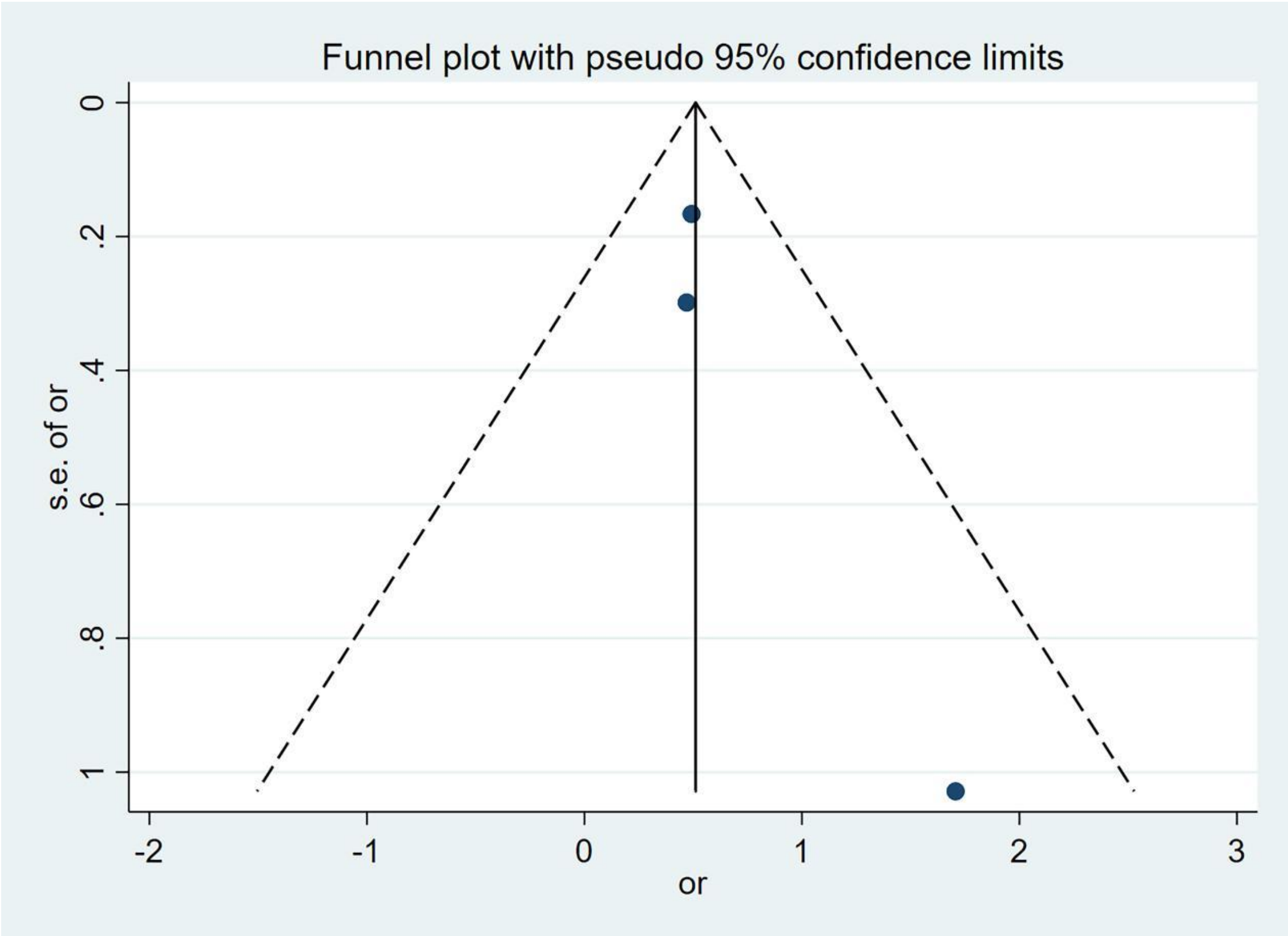

Figure S45 Funnel plot of history of seizures during treatment (Multi-factor)

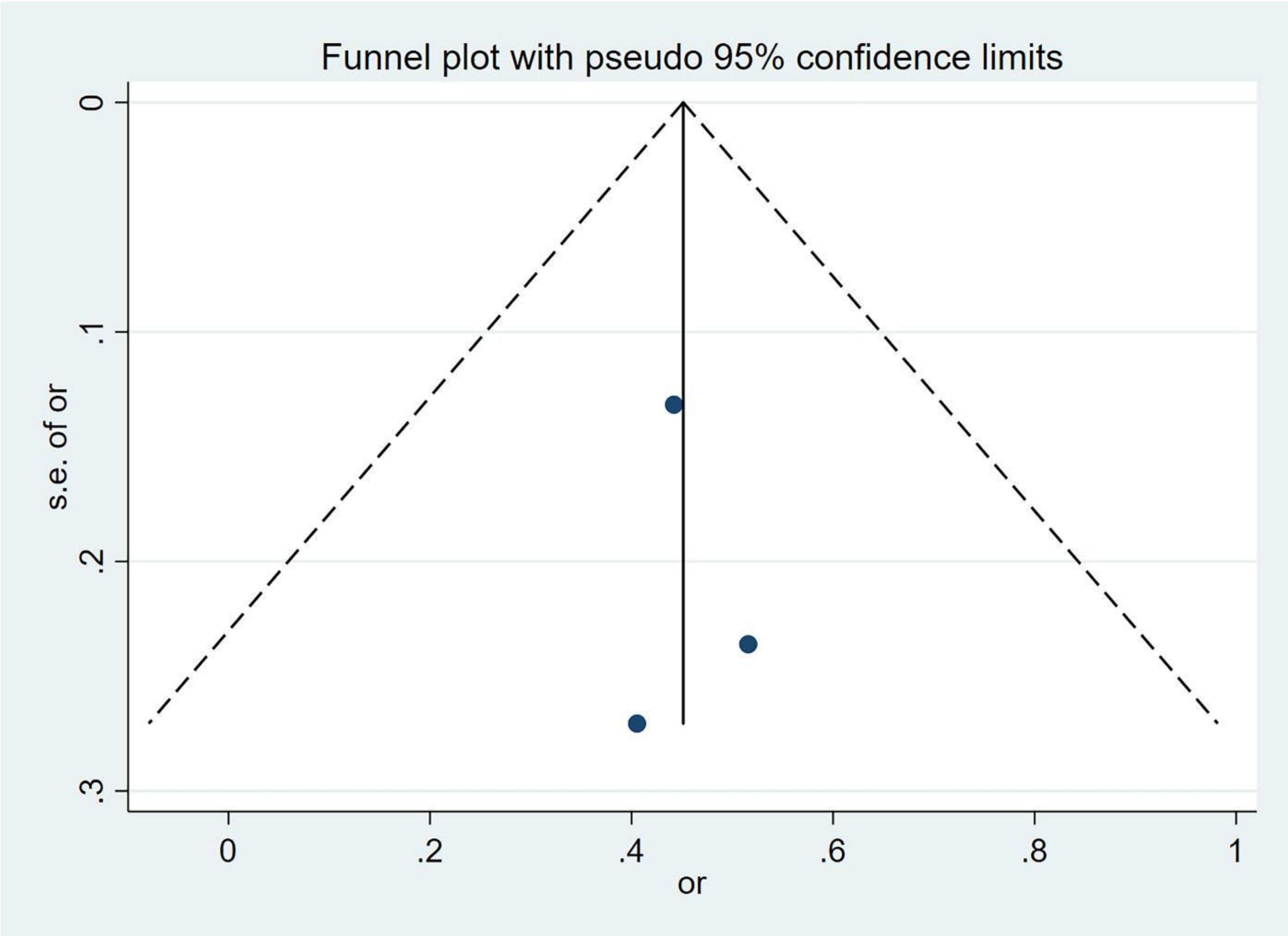

Figure S46 Funnel plot of history of seizures during treatment (Single factor)

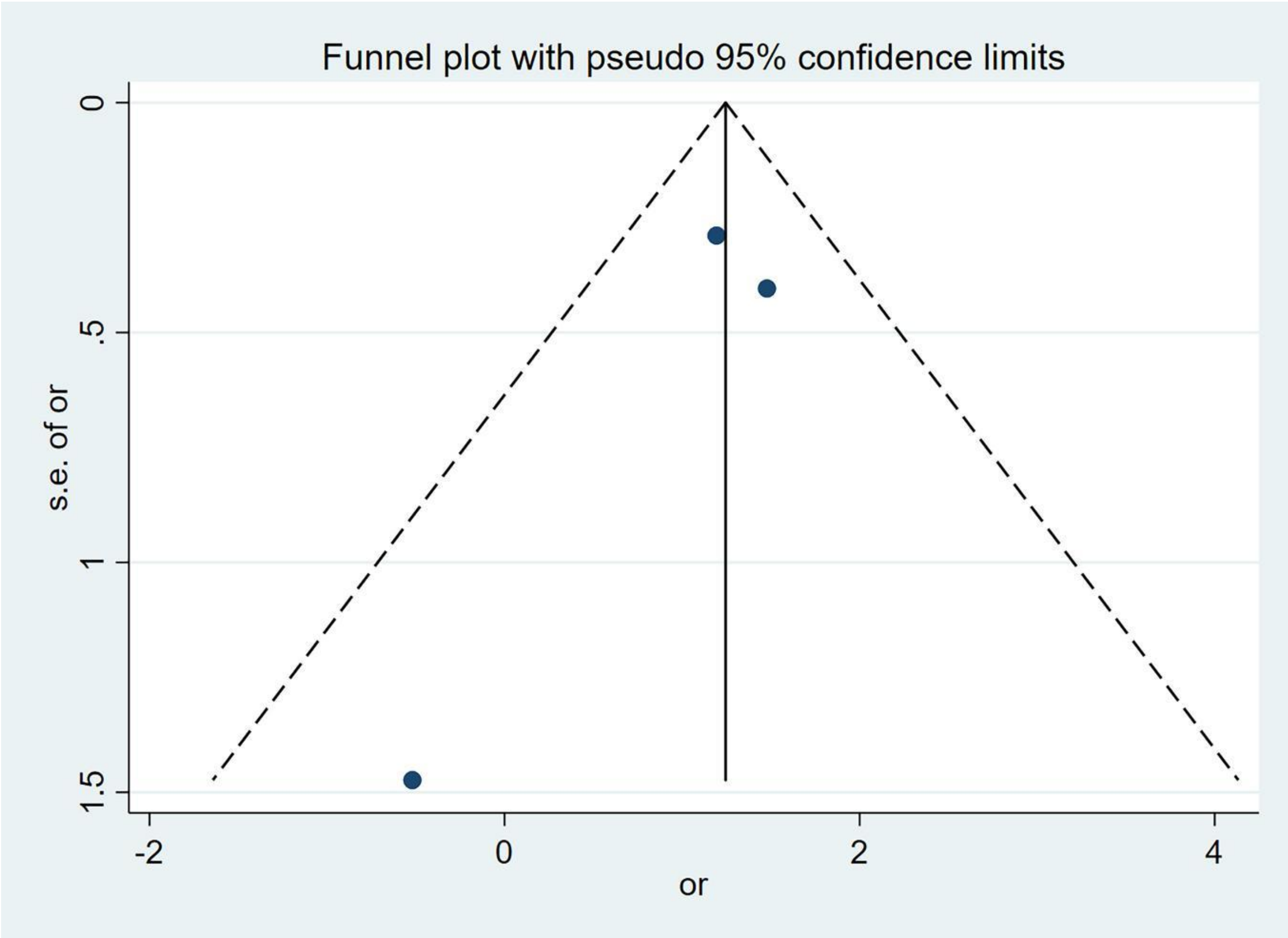

Figure S47 Funnel plot of symptomatic etiology (Multi-factor)

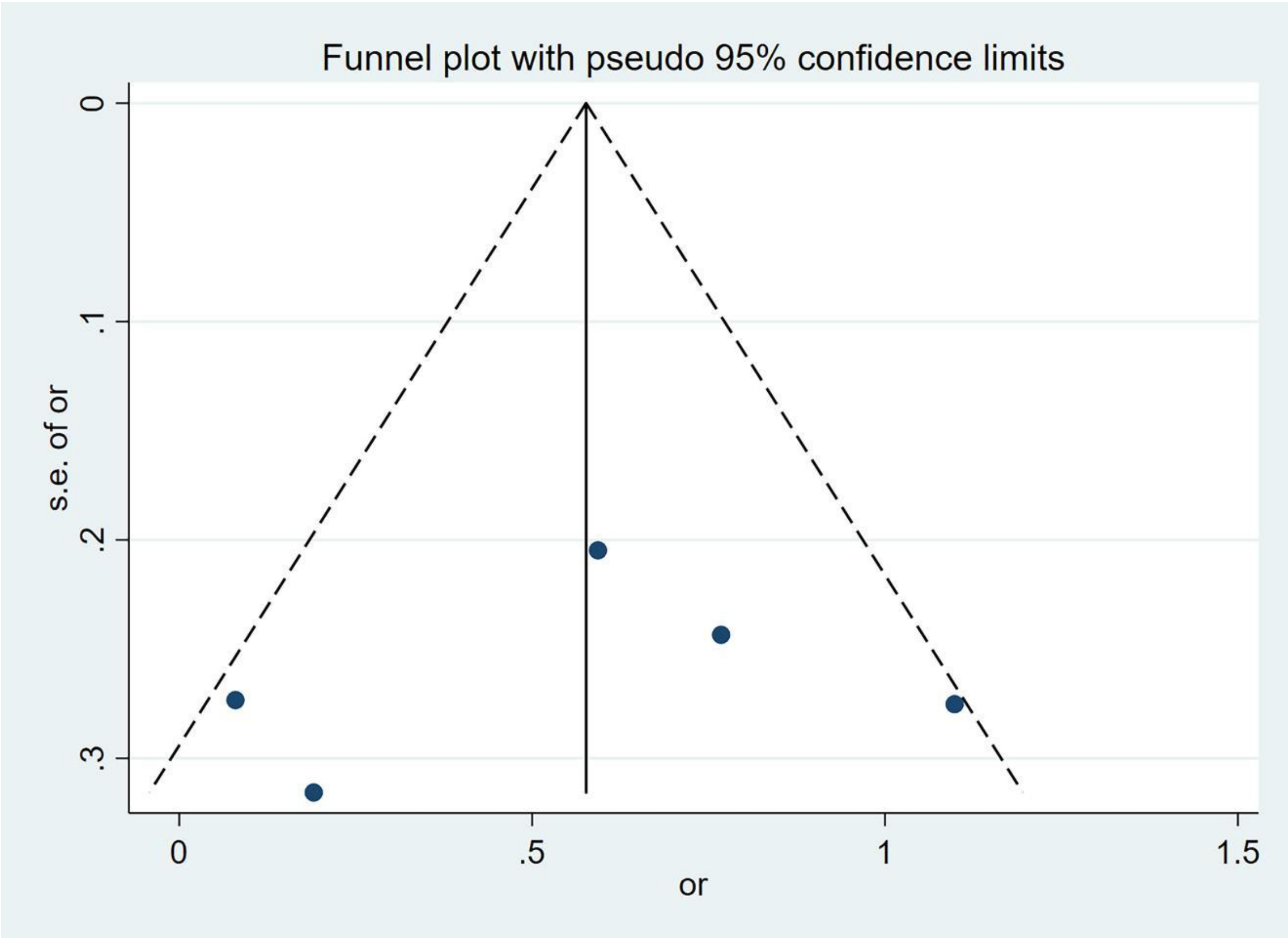

Figure S48 Funnel plot of symptomatic etiology (Single factor)

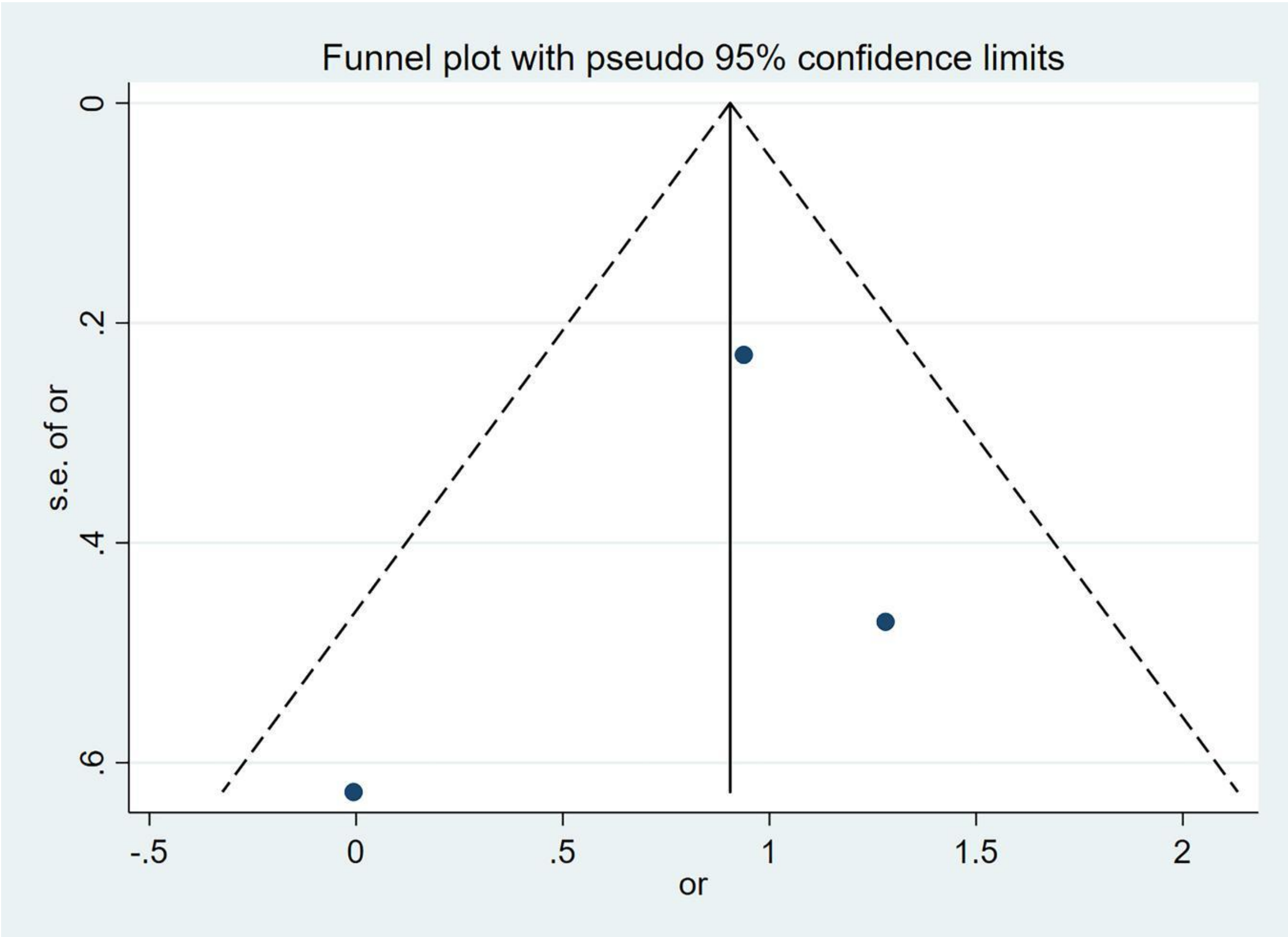

Figure S49 Funnel plot of history of neonatal seizures (Single factor)

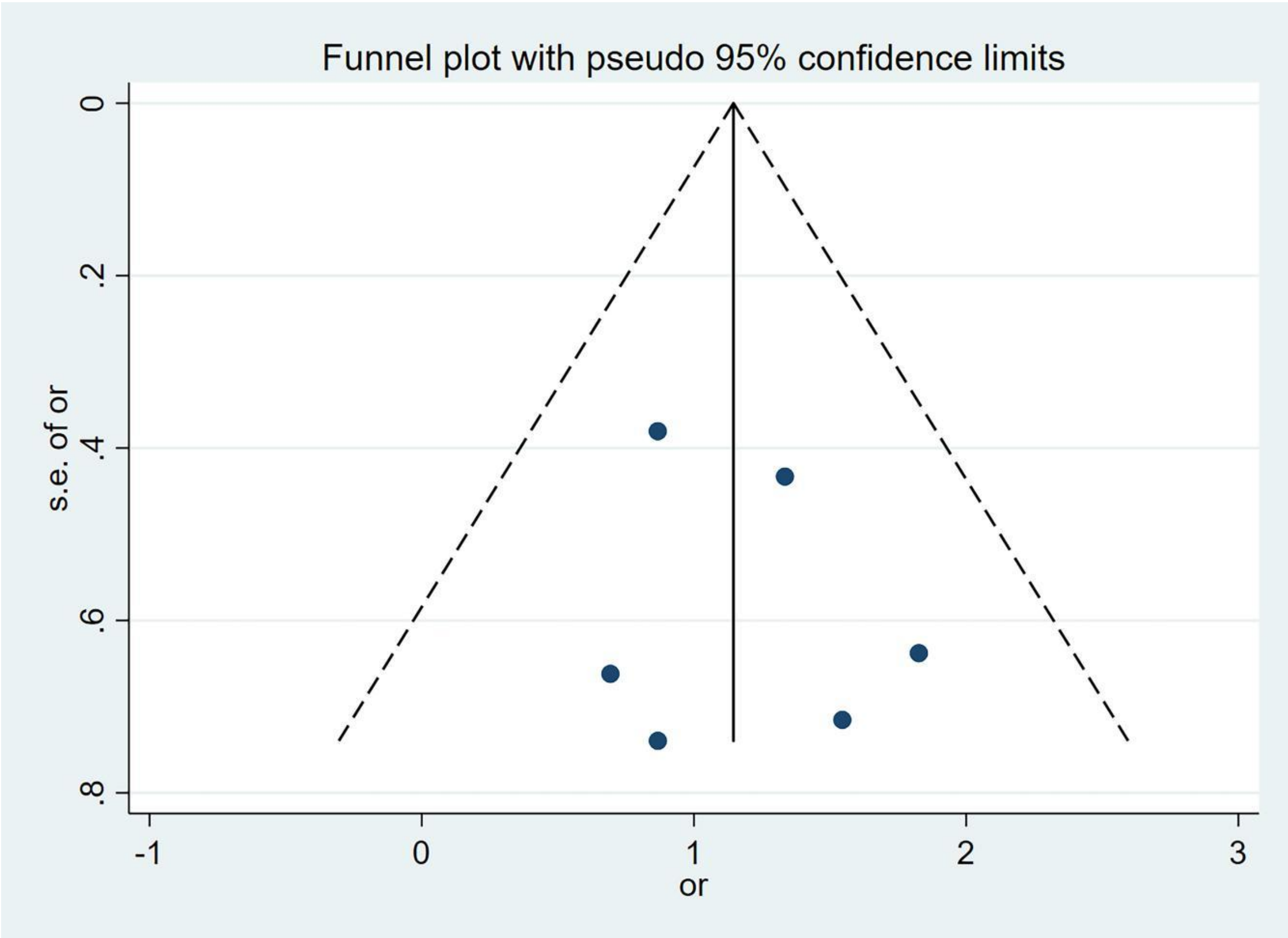

Figure S50 Funnel plot of abnormal EEG after withdrawal (Multi-factor)

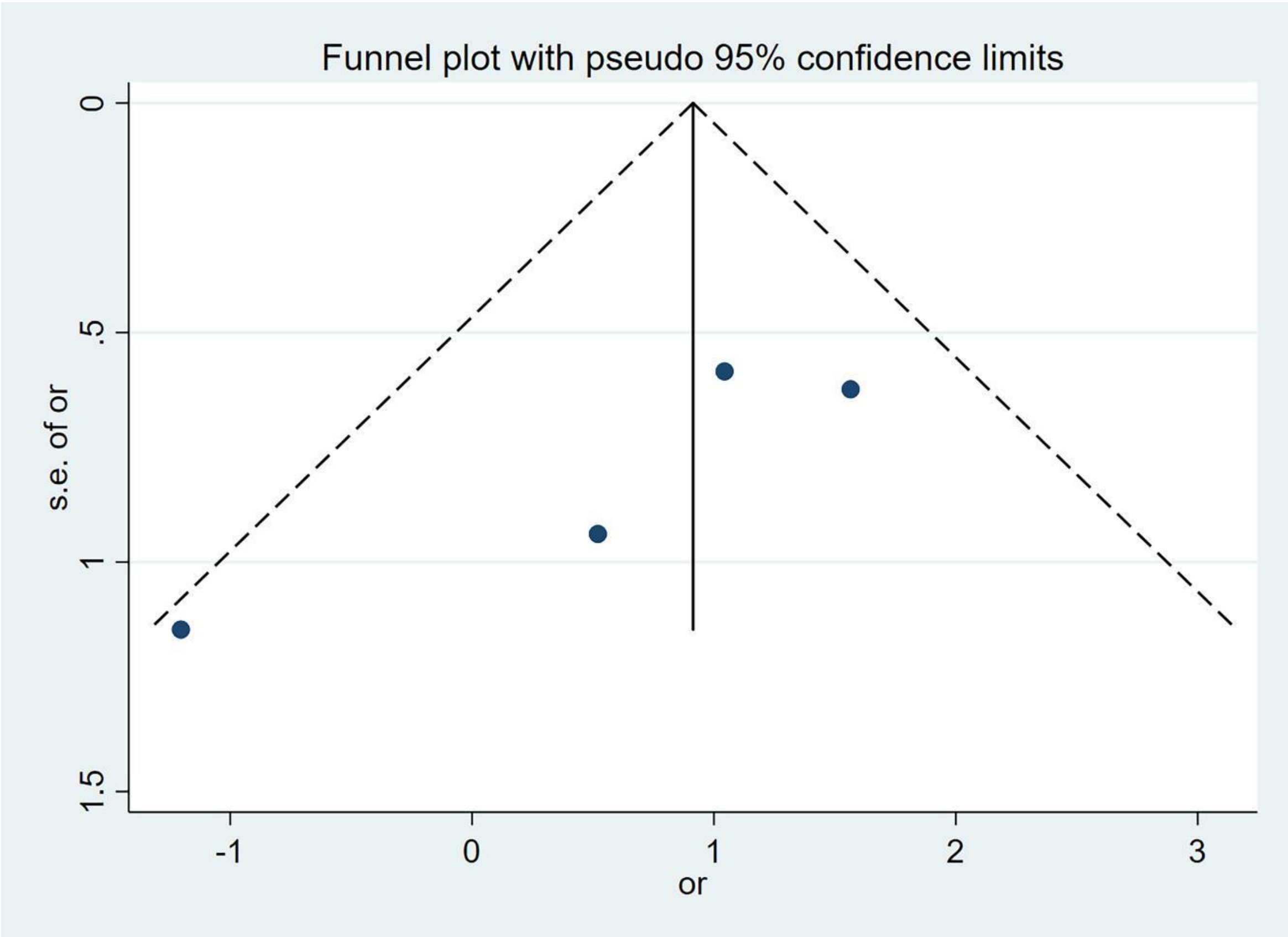

Figure S51 Funnel plot of polytherapy (Multi-factor)

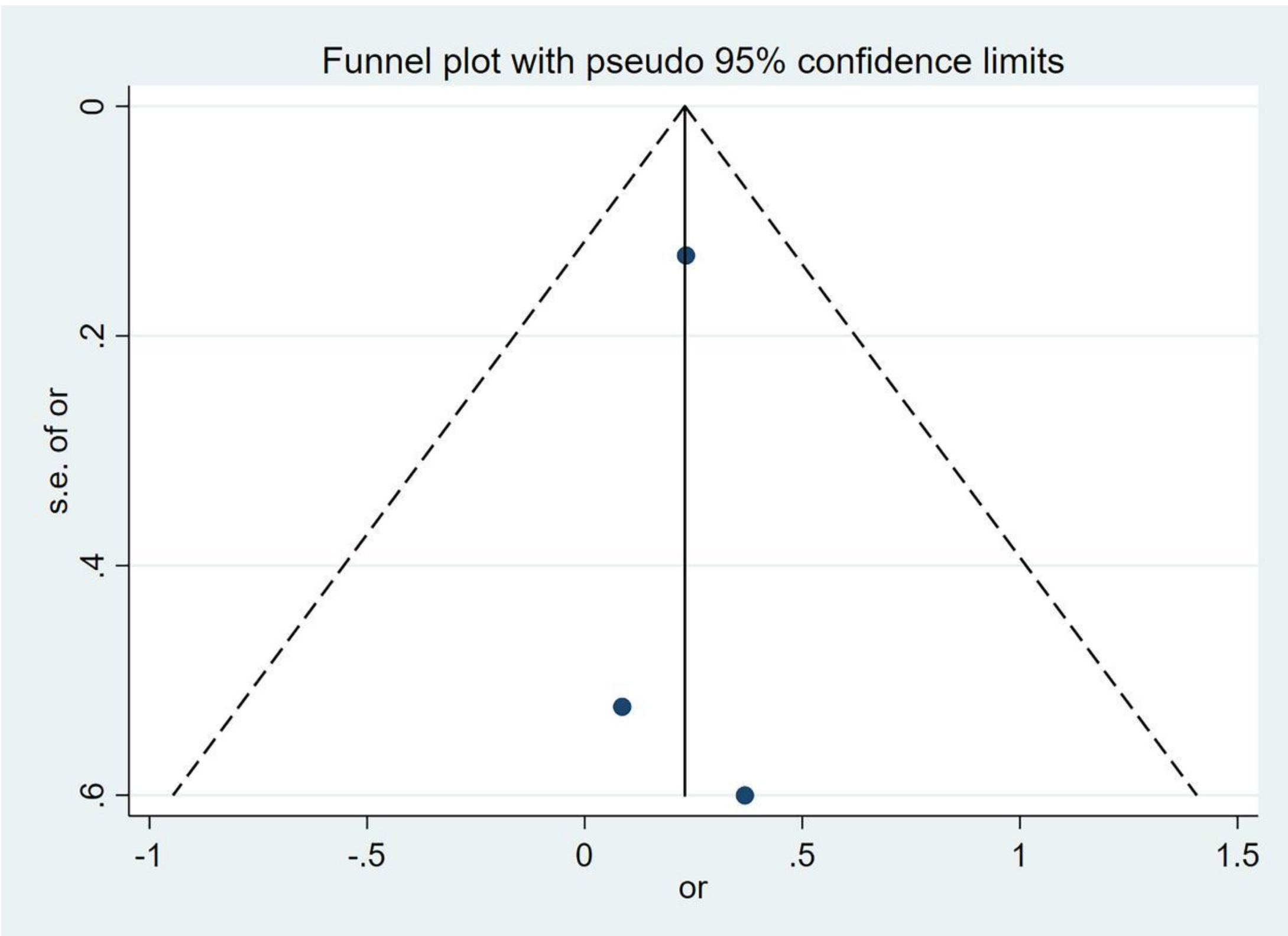

Figure S52 Funnel plot of abnormal EEG before withdrawal (Single factor)

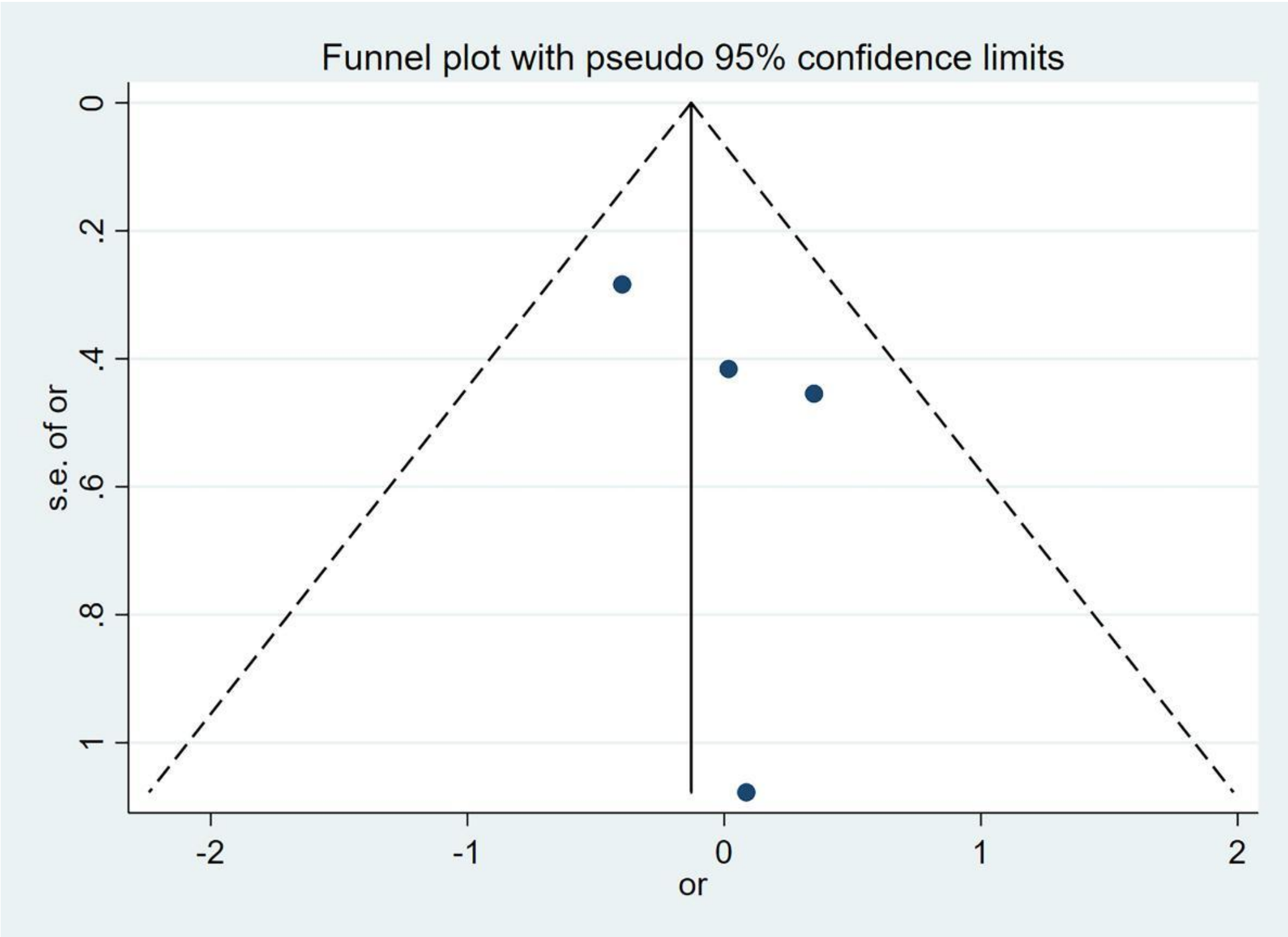

Figure S53 Funnel plot of history of status epilepticus (Single factor)

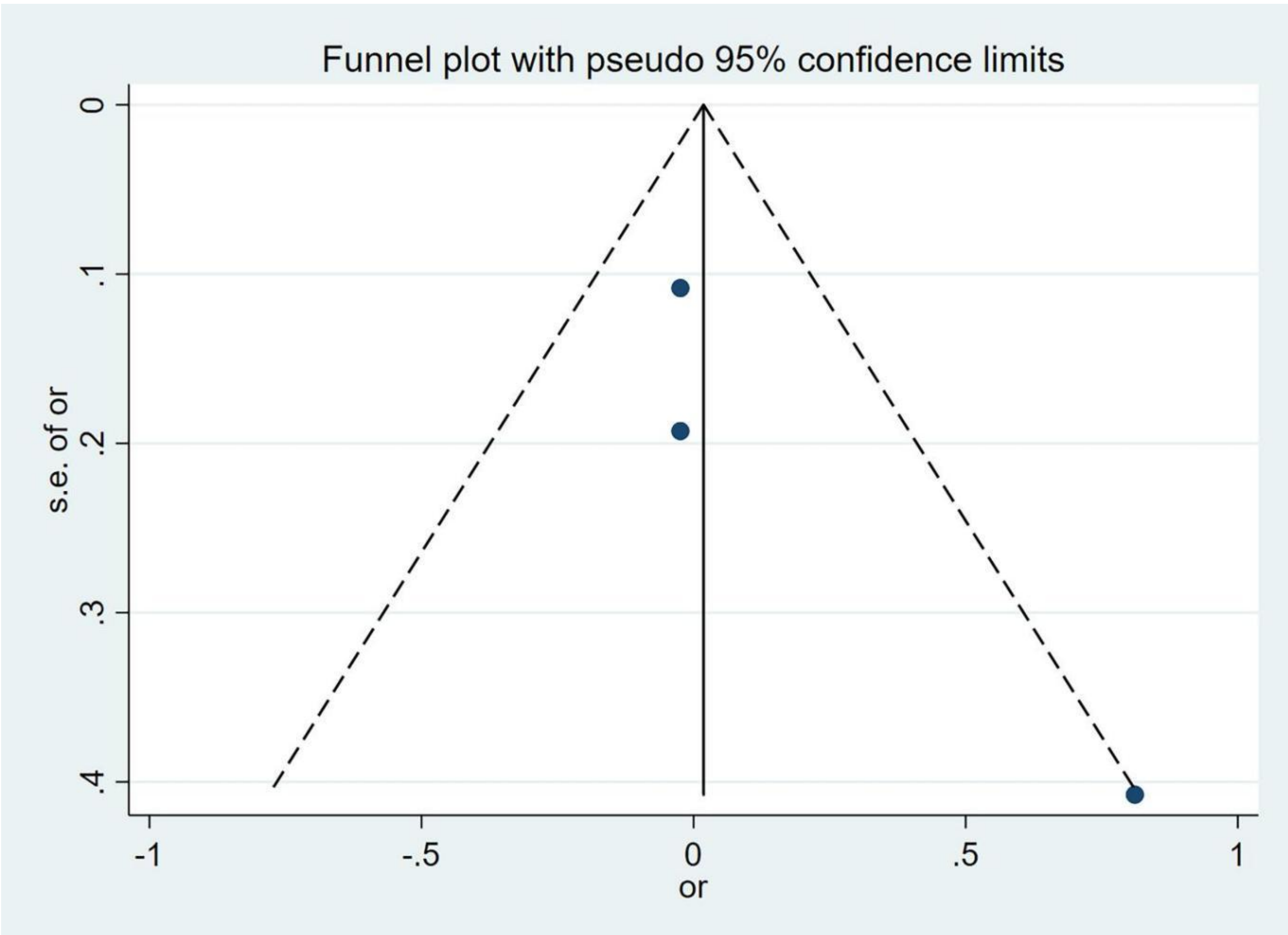

Figure S54 Funnel plot of seizure-free period＜3y (Single factor)

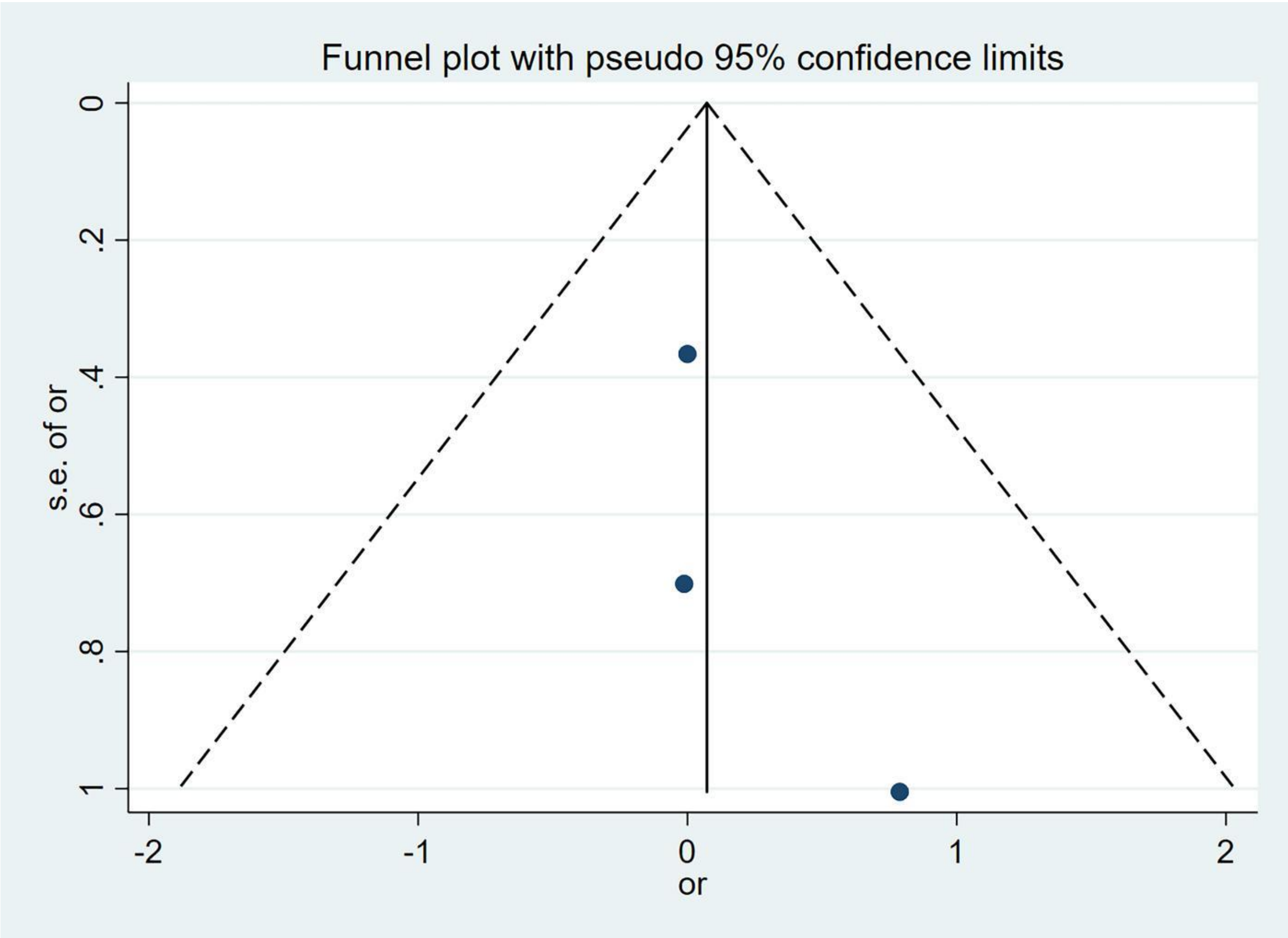

Figure S55 Funnel plot of seizures before treatment≥2 (Multi-factor)

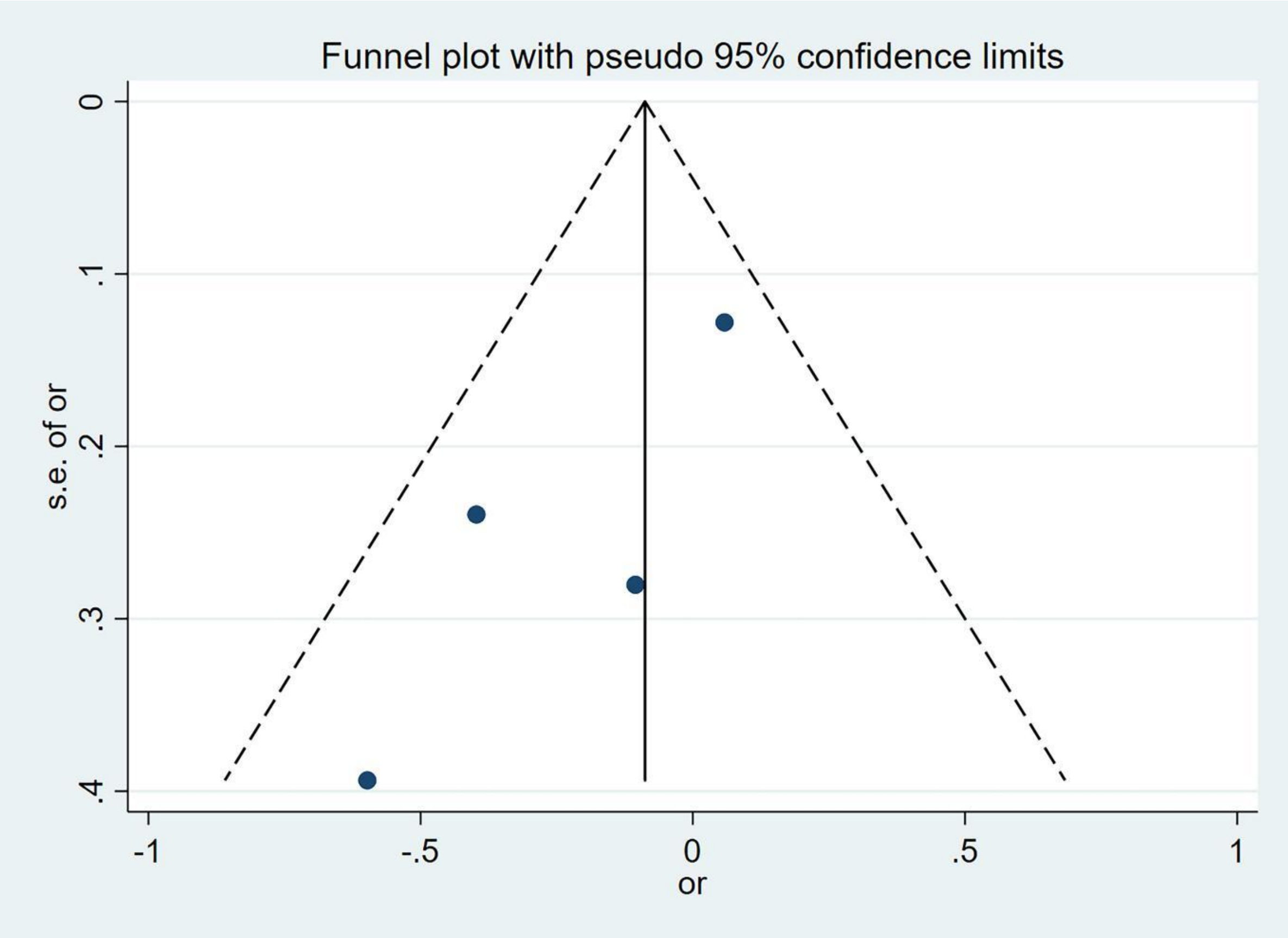

Figure S56 Funnel plot of seizures before treatment≥2 (Single factor)
